# Supplementary material for: Selection for constrained peptides that bind to a single target protein
Source: Nat Commun. 2021 Nov 3;12:6343. doi: 10.1038/s41467-021-26350-4 (PMC8566587; doi:10.1038/s41467-021-26350-4)
Supplement: Supplementary file 1 — Supplementary Information [file 41467_2021_26350_MOESM1_ESM.pdf]

Supplementary Information for:

Selection for constrained peptides that bind to a single target protein

Andrew M. King, Daniel A. Anderson, Emerson Glassey, Thomas H. Segall-Shapiro, Zhengan Zhang, David L. Niquille, Amanda C. Embree, Katelin Pratt, Thomas L. Williams, D. Benjamin Gordon, and Christopher A. Voigt

Supplementary Figures

Supplementary Figure 1
Supplementary Figure 2
Supplementary Figure 3
Supplementary Figure 4
Supplementary Figure 5

Supplementary Tables

Supplementary Table 1: Genetic parts used in this study
Supplementary Table 2: Plasmids used in this study
Supplementary Table 3: Primers used in this study
Supplementary Table 4: Pap2c library analysis

Supplementary Figures

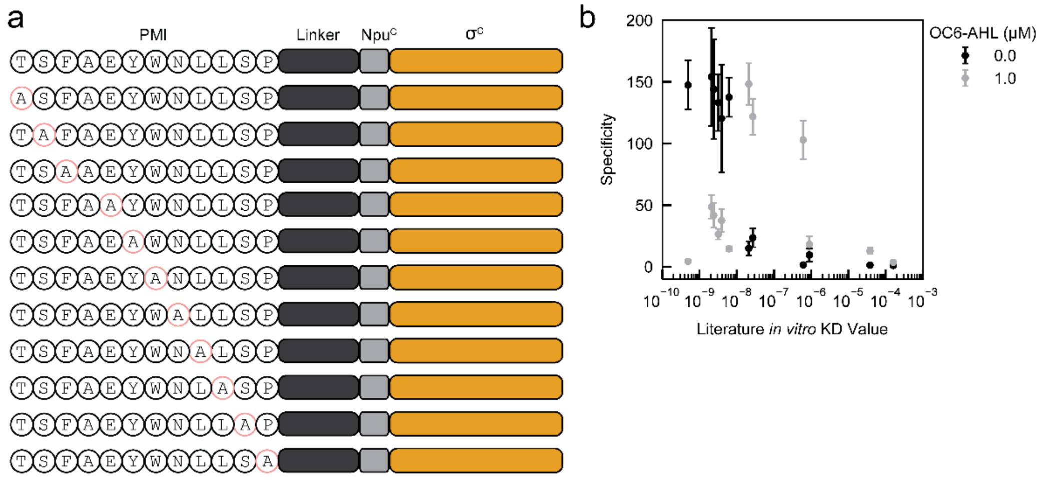

Supplementary Figure 1: Variable induction of split-intein system for protein-protein interactions reflects *in vitro* measurements. (a) Schematic of 12 PMI variants constructed for use in the split-intein assay. "TSFAEYWNLLSP" is the native PMI peptide sequence, alanine scans are represented by black circles. **(b)** Fluorescence output was measured for these 12 PMI variants binding to Mdm2* and plotted against their previously reported *in vitro* K_D values. The specificity is defined as the central measure and error bars in parts X were calculated as $\sigma = |A/B|((\sigma\_A/A)^2+(\sigma\_B/B)^2]^{1/2}$, where A is the mean of the on-target (Mdm2) fluorescence, B is the mean of the off-target (RBD) fluorescence, and σ_A and σ_B are the standard deviations of their measurements. For all measurements, three biological replicates were performed on three different days.

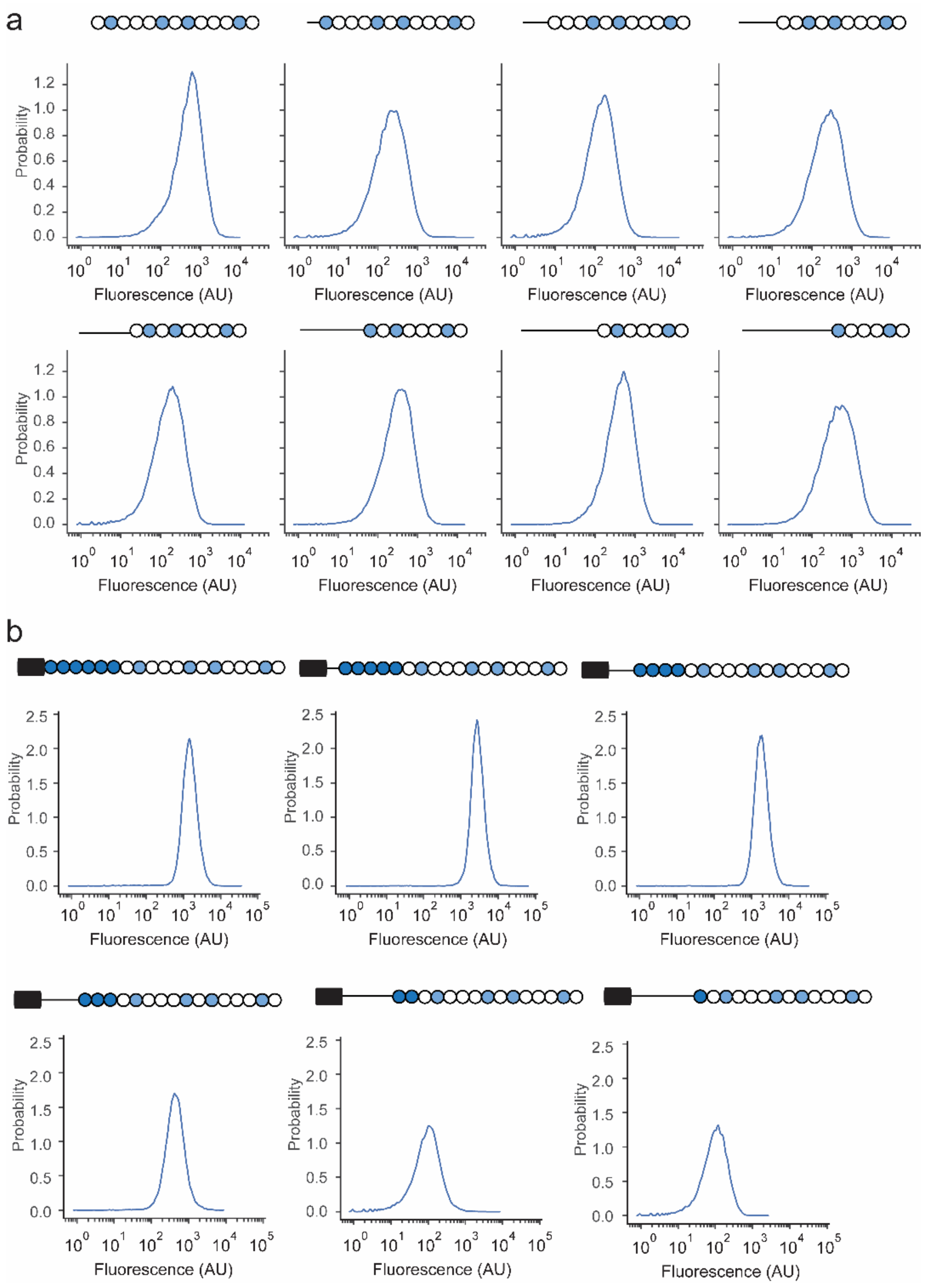

Supplementary Figure 2: Raw output of Pap2c_1 *in vivo* SAR profiling. Cytometry distributions for data found in bar plots from Figure 3. **(a)** Example raw data for core truncations (Figure 3a). **(b)** Example raw data for TEV-leader truncations (Figure 3d). Single replicate data of fluorescence of the sfGFP fused to CAT is shown.

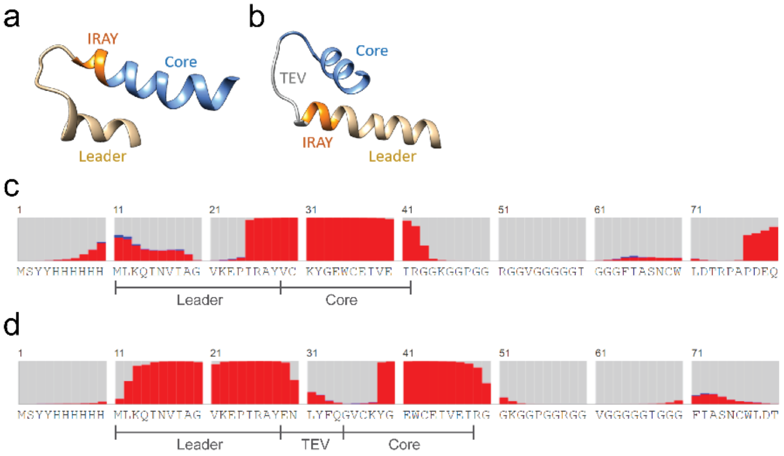

Supplementary Figure 3: Extended structure-activity relationship of Pap2c_1 *in vivo*. (a, b) Predicted secondary structure of parent Pap2c_1 peptide **(a)** and the Pap2c_1 with the Leader-TEV protease site-Core architecture **(b)**. Models in were generated using Robetta with default parameters [1]. **(c)** Secondary structure prediction of the first 80 amino acids of the parent Pap2c_1 peptide. **(d)** Secondary structure prediction of the first 80 amino acids of the parent Pap2c_1 peptide with the Leader-TEV protease site-Core architecture. For **c** and **d**, predictions were generated using the RaptoX property online tool [2].

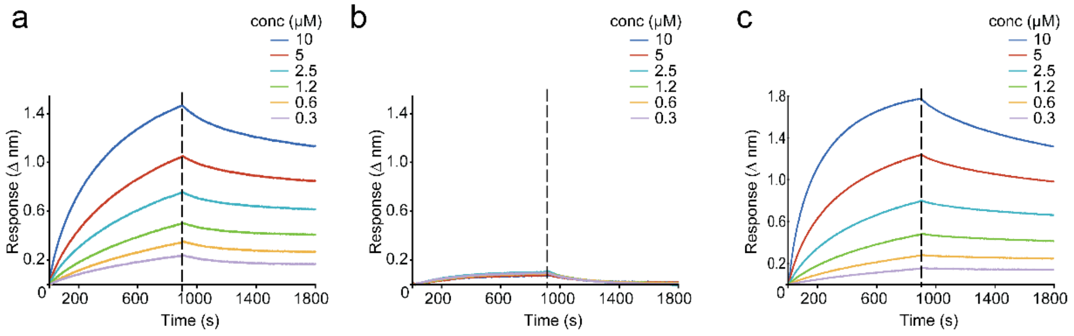

Supplementary Figure 4: Kinetics of peptide:RBD BLI measurements. (a) Binding of purified, modified AMK-1057 to human-derived Spike RBD measured via BLI. **(b)** Binding of purified, unmodified AMK-1057 to human-derived Spike RBD measured via BLI. **(c)** Binding of purified ACE2* to human-derived Spike RBD measured via BLI. For all experiments, vertical dotted lines indicate the dissociation phases of the measurements. Three replicates were run on three different days and a representative trace is shown. Replicates are used to calculate the standard deviations presented in the text

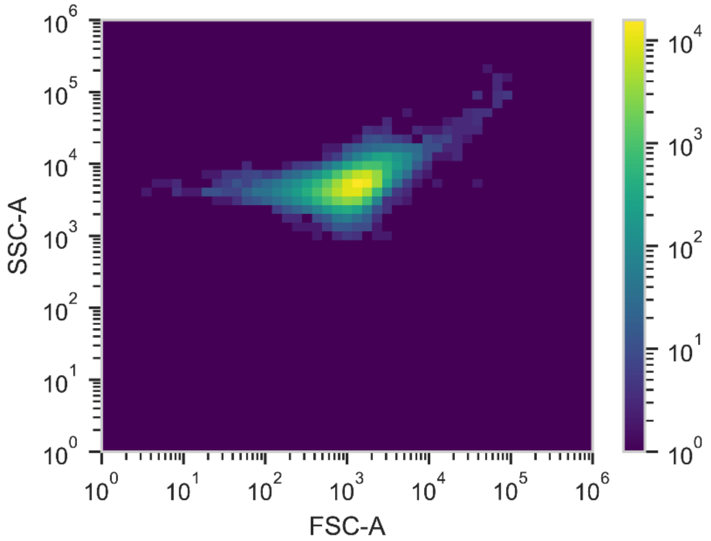

Supplementary Figure 5: Representative forward and side scatter of *E. coli* cells used in this study. Cytometry plot shown is from replicate 3 of pap2c_1 targeting the RBD from Figure 2f. 30,000 events were collected and no gating was performed on the data. Figure generated in Python using Cytoflow version 1.0. FSC-A, Forward scatter; SSC-A, side scatter.

Supplementary Tables

Supplementary Table 1: Genetic Parts

Promoters

Name	Sequence	Source
P_{J23105}	GGCGCGCCTTTACGGCTAGCTCAGTCCTAGGTACTATGCTAGCAAGGT	iGEM
P_{LuxB}	ACCTGTAGGATCGTACAGGTTTACGCAAGAAAATGGTTTGTTACAGTCGAATAAA	[3]
P_{LacI}	GCGGCGCGCCATCGAATGGCGCAAAACCTTTCGCGGTATGGCATGATAGCGCCC	[3]
P_{Tac}	TGTTGACAATTAATCATCGGCTCGTATAATGTGTGGAATTGTGAGCGCTCACAATT	[3]
P_{ECF20_992}	GCGCGGATAAAAATTTCATTTGCCCGCGACGGATTCCCCGCCCATCTATCGTTGAACCCATCAGCTGCGTTCATCAGCGA	[4]
P_{T5LacO}	AATCATAAAAAATTTATTTGCTTTGTGAGCGGATAACAATTATAATAGATTCAATTGTGAGCGGATAACAATT	[3]
P_{CymRC}	AACAAACAGACAATCTGGTCTGTTTGTATTATGGAAAATTTTTCTGTATAATAGATTCAACAAACAGACAATCTGGTCTGTTTGTATTAT	[3]

Ribosome binding sites (RBSs)

Gene	Name	Sequence	Source
sfGFP	B0032	TCACACAGGAAAG	iGEM
npu^N	RL1_6	TCATGGACGAAAG	This work
PheS	B0064	AAAGAGGGGAAA	iGEM
PapB	ME_44	AGAACTAAGGAGGTTAGAGG	RBS Calc
SUMO	PP_1	ACCCAACACCACCAGCAAGCCTAAGGAGGAGAAAT	RBS Calc
CymR	cym2	AATGGAATATTCATAGTAAATACCAAACGGAGATTCTT	[3]
peptide	PI_1	TCCATCTCTATGGCGGATTTT	This work
LuxR	lux1	GGAAGAGAGTCAATTCAGGGTGGTGAAT	[3]

Terminators

Name	Details	Sequence	
B0062		CAGATAAAAAAAATCCTTAGCTTTCGCTAAGGATGATTTCT	iGEM
B0010		CCAGGCATCAAATAAAACGAAAGGCTCAGTCGAAAGACTGGGCCTTTCGTTTTATCTGTTGTTTGTCGGTGAACGCTCTC	iGEM
B0012		TCACACTGGCTCACCTTCGGGTGGGCCTTTCTGCGTTTATA	iGEM
L3S3P21		CCAATTATTGAAGGCCTCCCTAACGGGGGGCCTTTTTTTGTTTCTGGTCTCCC	[5]
IOT		TTGGTAACGAATCAGACAATTGACGGCTCGAGGGAGTAGCATAGGGTTTGCAGAATCCCTGCTTCGTCCATTTGACAGGCACATTATGCATCGATGATAAGCTGTCAAACATGAGCAGATCCTCTACGCCGGACGCATCGTGGCCGGCATCACCGGCGCCACAGGTGCGGTTGCTGGCGCCTATATCGCCGACATCACCGATGGGGAAGATCGGGCTCGCCACTTCGGGCTCATGAGCAAATATTTTATCTG	[3]
L3S2P11		CTCGGTACCAAATTCCAGAAAAGAGACGCTTTCGAGCGTCTTTTTTCGTTTTGGTCC	[5]
L3S3P41	g->c SNP to remove BsaI site. Predicted to not affect the hairpin by Vienna [6]	AAAAAAAAAAAACACCCTAACGGGTGTTTTTTTTTTTTTGGTGTCCC	[5]
AraC	w/ 2 SNPs	TTGGTAACGAATCAGACAATTGACGGCTCGAGGGAGTAGCATAGGGTTTGCAGAATCCCTGCTTCGTCCATTTGACAGGCACATTATGCATCGATGATAAGCTGTCAAACATGAGCA	[3]
B0053	aka His Operon Terminator	TCCGGCAAAAAAGGGCAAGGTGTCACCACCCTGCCCTTTTTCTTTAAAACCGAAAAGATTACTTCGCGTT	[3]

Ribozymes

Name	Details	Sequence	Source
RiboJ		AGCTGTCACCGGATGTGCTTTCCGGTCTGATGAGTCCGTGAGGACGAAACAGCCTCTACAAATAA<br>TTTTGTTTAA	[7]
RiboJ53		AGCGGTCAACGCATGTGCTTTGCGTTCTGATGAGACAGTGATGTCGAAACCGCCTCTACAAATAA<br>TTTTGTTTAA	[7]
ElvJ		AGCCCCATAGGGTGGTGTGTACCACCCCTGATGAGTCCAAAAGGACGAAATGGGGCCTCTACAA<br>ATAATTTTGTTTAA	[7]

Linkers/Tags

Name	Details	Sequence	Source
ATag-2	N-terminal SUMO affinity tag	ATGTCATATTACCACCATCACCATCATCACGGGTCCCTGCAG	
Link-2	N-terminal SUMO linker v2	TGCATGTCATATTACGACTCCATTCCCACAAGCGAGAACTTGTACTTTCAAGGGTGC	
RST_N	Concatenation of: ATag-2, SUMO, and Link-2	ATGTCATATTACCACCATCACCATCATCACGGGTCCCTGCAGGACTCAGAAGTCAATCAAGAAGC<br>TAAGCCAGAGGTCAAGCCAGAAGTCAAGCCTGAGACTCACATCAATTTAAAGGTGTCCGATGGAT<br>CTTCAGAGATCTTCTTCAAGATCAAAAAGACCACTCCTTTAAGAAGGCTGATGGAAGCGTTCGCTA<br>AAAGACAGGGTAAGGAAATGGACTCCTTAAGATTCTTGTACGACGGTATTAGAATTCAAGCTGATC<br>AGGCCCCTGAAGATTTGGACATGGAGGATAACGATATTATTGAGGCTCACCGCGAACAGATTGGA<br>GGTTGCATGTCATATTACGACTCCATTCCCACAAGCGAGAACTTGTACTTTCAAGGGTGC	
Npu^C-Link	Peptide-Npu^C-σ^C linker	GGAGGTAAGGGAGGACCTGGAGGTCGGGGAGGTGTTGGAGGTGGTGGAGGAATTGGAGGT	This work
Npu^N-Link	σ^N-Npu^N-Bait linker	GGAGGATCTGGTGGAAGTGGTGGTTCTGGAGGT	This work

Genes

Name	Details	Sequence	Source
SUMO	sequence from pE-SUMO	GACTCAGAAGTCAATCAAGAAGCTAAGCCAGAGGTCAAGCCAGAAGTCAAGCCTGAGACTCACAT<br>CAATTTAAAGGTGTCCGATGGATCTTCAGAGATCTTCTTCAAGATCAAAAAGACCACTCCTTTAAG<br>AAGGCTGATGGAAGCGTTCGCTAAAAGACAGGGTAAGGAAATGGACTCCTTAAGATTCTTGTACG<br>ACGGTATTAGAATTCAAGCTGATCAGGCCCCTGAAGATTTGGACATGGAGGATAACGATATTATTG<br>AGGCTCACCGCGAACAGATTGGAGGT	Life-Sensors
lacI		ATGAAACCAGTAACGTTATACGATGTCGCAGAGTATGCCGGTGTCTCTTATCAGACCGTTTCCCG<br>CGTGGTGAACCAGGCCAGCCACGTTTCTGCGAAAACGCGGGAAAAAGTGGAAGCGGCGATGGC<br>GGAGCTGAATTACATTCCCAACCGCGTGGCACAACAACTGGCGGGCAAACAGTCGTTGCTTATTG<br>GCGTTGCCACCTCCAGTCTGGCCCTGCACGCGCCGTCGCAAATTGTCGCGGCGATTAAATCTCG<br>CGCCGATCAACTGGGTGCCAGCGTGGTGGTGTCGATGGTAGAACGAAGCGGCGTCGAAGCCTG<br>TAAAGCGGCGGTGCACAATCTTCTCGCGCAACGCGTCAGTGGGCTGATCATTAACTATCCGCTG<br>GATGACCAGGATGCCATTGCTGTGGAAGCTGCCTGCACTAATGTTCCGGCGTTATTTCTTGATGT<br>CTCTGACCAGACACCCATCAACAGTATTATTTTCTCCCATGAGGACGGTACGCGACTGGGCGTGG<br>AGCATCTGGTCGCATTGGGTCACCAGCAAATCGCGCTGTTAGCGGGCCCATTAAGTTCTGTCTCG<br>GCGCGTCTGCGTCTGGCTGGCTGGCATAAATATCTCACTCGCAATCAAATTCAGCCGATAGCGGA<br>ACGGGAAGGCGACTGGAGTGCCATGTCCGGTTTTCAACAAACCATGCAAATGCTGAATGAGGGC<br>ATCGTTCCCACTGCGATGCTGGTTGCCAACGATCAGATGGCGCTGGGCGCAATGCGCGCCATTA<br>CCGAGTCCGGGCTGCGCGTTGGTGCGGATATCTCGGTAGTGGGATACGACGATACCGAAGATAG<br>CTCATGTTATATCCCGCCGTTAACCACCATCAAACAGGATTTTCGCCTGCTGGGGCAAACCAGCG<br>TGGACCGCTTGCTGCAACTCTCTCAGGGCCAGGCGGTGAAGGGCAATCAGCTGTTGCCAGTCTC<br>ACTGGTGAAAAGAAAAACCACCCTGGCGCCCAATACGCAAACCGCCTCTCCCCGCGCGTTGGCC<br>GATTCATTAATGCAGCTGGCACGACAGGTTTCCCGACTGGAAAGCGGGCAG	[3]
luxR		ATGAAAAACATAAATGCCGACGACACATACAGAATAATTAATAAAATTAAAGCTTGTAGAAGCAATA<br>ATGATATTAATCAATGCTTATCTGATATGACTAAAATGGTACATTGTGAATATTATTTACTCGCGAT<br>CATTTATCCTCATTCTATGGTTAAATCTGATATTTCAATCCTAGATAATTACCCTAAAAAATGGAGG<br>CAATATTATGATGACGCTAATTTAATAAAATATGATCCTATAGTAGATTATTCTAACTCCAATCATTC<br>ACCAATTAATTGGAATATATTTGAAAACAATGCTGTAAATAAAAAATCTCCAAATGTAATTAAAGAA<br>GCGAAAACATCAGGTCTTATCACTGGGTTTAGTTTCCCTATTCATACGGCTAACAATGGCTTCGGA<br>ATGCTTAGTTTTGCACATTCAGAAAAAGACAACTATATAGATAGTTTATTTTTACATGCGTGTATGA<br>ACATACCATTAATTGTTCCTTCTCTAGTTGATAATTATCGAAAAATAAATATAGCAAATAATAAATCA<br>AACAACGATTTAACCAAAAGAGAAAAAGAATGTTTAGCGTGGGCATGCGAAGGAAAAAGCTCTTG<br>GGATATTTCAAAAATATTAGGTTGCAGTGAGCGTACTGTCACTTTCCATTTAACCAATGCGCAAAT<br>GAAACTCAATACAACAAACCGCTGCCAAAGTATTTCTAAAGCAATTTTAACAGGAGCAATTGATTG<br>CCCATACTTTAAAAATTGATAA	[3]
cymR		ATGAGCCCGAAACGTCGTACCCAGGCAGAACGTGCAATGGAAACCCAGGGTAAACTGATTGCAG<br>CAGCACTGGGTGTTCTGCGTGAAAAAGGTTATGCAGGTTTTCGTATTGCAGATGTTCCGGGTGCA<br>GCCGGTGTTAGCCGTGGTGCACAGAGCCATCATTTTCCGACCAAACTGGAACTGCTGCTGGCAA<br>CCTTTGAATGGCTGTATGAGCAGATTACCGAACGTAGCCGTGCACGTCTGGCAAAACTGAAACCG<br>GAAGATGATGTTATTCAGCAGATGCTGGATGATGCAGCAGATTTTTTTCTGGATGATGATTTTAGC<br>ATCGGCCTGGATCTGATTGTTGCAGCAGATCGTGATCCGGCACTGCGTGAAGGTATTCTGCGTAC<br>CGTTGAACGTAATCGTTTTGTTGTTGAAGATATGTGGCTGGGTGTGCTGGTGAGCCGTGGTCTGA<br>GCCGTGATGATGCCGAAGATATTCTGTGGCTGATTTTTAACAGCGTTCGTGGTCTGACAGTTCGT<br>AGCCTGTGGCAGAAAGATAAAGAACGTTTTGAACGTGTGCGTAATAGCACCCTGGAAATTGCACG<br>TGAACGTTATGCAAAATTCAAACGT	[3]
sfGFP		ATGAGCAAAGGAGAAGAACTTTTCACTGGAGTTGTCCCAATTCTTGTTGAATTAGATGGTGATGTT<br>AATGGGCACAAATTTTCTGTCCGTGGAGAGGGTGAAGGTGATGCTACAAACGGAAAACTCACCCT	[3]

		TAAATTTATTTGCACTACTGGAAAACTACCTGTTCCGTGGCCAACACTTGTCACTACTCTGACCTAT<br>GGTGTTCAATGCTTTTCCCGTTATCCGGATCACATGAAACGGCATGACTTTTTCAAGAGTGCCATG<br>CCCGAAGGTTATGTACAGGAACGCACTATATCTTTCAAAGATGACGGGACCTACAAGACGCGTGC<br>TGAAGTCAAGTTTGAAGGTGATACCCTTGTTAATCGTATCGAGTTAAAGGGTATTGATTTTAAAGA<br>AGATGGAAACATTCTTGGACACAAACTCGAGTACAACTTTAACTCACACAATGTATACATCACGGC<br>AGACAAACAAAAGAATGGAATCAAAGCTAACTTCAAAATTCGCCACAACGTTGAAGATGGTTCCGT<br>TCAACTAGCAGACCATTATCAACAAAATACTCCAATTGGCGATGGCCCTGTCCTTTTACCAGACAA<br>CCATTACCTGTCGACACAATCTGTCCTTTCGAAAGATCCCAACGAAAAGCGTGACCACATGGTCC<br>TTCTTGAGTTTGTAACTGCTGCTGGGATTACACATGGCATGGATGAGCTCTACAAA	
ace2α1	ACE2 residues 19-56	GTCAACGATCGAAGAACAGGCTAAAACGTTCCTGGATAAGTTCAATCATGAGGCGGAGGACCTGT<br>TCTACCAAAGCAGCTTGGCCTCTTGGAACTACAACACGAACATTACGGAG	IDT; adapted from [8]
npu^C	Npu intein C-terminal fragment	GGTTTTATCGCTTCCAACTGCTGG	IDT; adapted from [9]
σ^C	ecf20_992 sigma factor C-terminal fragment	CTGGATACCCGTCCGGCACCGGATGAACAGCTGGAAGCAAGCGCACAGAGCCGTCGTATGGCA<br>CAGGCACTGGATCAGCTGCCGGATCGTCAGCGTGAAGCAATTGTTCTGCAGTATTATCAAGAACT<br>GAGCAATACCGAAGCAGCAGCACTGATGCAAATTAGCGTTGAAGCCCTGGAAAGCCTGCTGAGC<br>CGTGCACGTCGTAATCTGCGTAGCCATCTGGCCGAAGCACCGGGTGCAGATCTGAGCGGTCGTC<br>GCAAACCG	[4]
npu^N	Npu intein N-terminal fragment	ATGAATGAAACCGATCCTGATCTGGAACTGCTGAAACGTATTGGTAATAATGATGCACAGGCCGT<br>TAAAGAAATGGTTACCCGTAAACTGCCTCGTCTGCTGGCACTGGCAAGTCGCCTGCTGGGTGATG<br>CAGATGAAGCACGTGATATTGCACAAGAAAGTTTTCTGCGCATTTGGAAACAGGCAGCAAGCTGG<br>CGTAGCGAACAGGCACGTTTTGATACCTGGCTGCATCGTGTTGCACTGAATCTGTGTTATGATCG<br>TCTGCGTCGTCGTAAAGAACATGTGCCGGTTGATAGCGAACATGCCTGTGAAGCA	IDT; adapted from [9]
σ^N	ecf20_992 sigma factor N-terminal fragment	TGCCTGAGCTACGAAACCGAAATCCTGACCGTTGAATATGGTCTGCTGCCGATCGGCAAAATCGT<br>AGAAAAGCGTATCGAATGTACGGTTTACTCTGTCGATAACAACGGTAACATCTACACCCAGCCGG<br>TAGCGCAGTGGCACGACCGTGGCGAACAAGAAGTGTTCGAGTACTGCCTGGAGGATGGCTCTCT<br>GATCCGCGCTACTAAAGACCACAAATTTATGACCGTGGACGGTCAAATGCTGCCGATCGATGAAA<br>TCTTTGAGCGCGAACTGGACCTGATGCGCGTGGACAACCTGCCGAACATCAAAATTGCTACCCG<br>CAAGTATCTGGGTAAGCAGAACGTCTATGACATTGGTGTGGAGCGCGACCACAATTTCGCTCTGA<br>AAAAC	[4]
pmi	P53 peptide derivative	ATGACCAGCTTTGCGGAATATTGGAACCTGCTGAGCCCG	IDT; adapted from [10]
mdm2*	Mdm2 residues 2-128 (translational fusion)	AGCCAGATCCCGGCGTCCGAACAAGAAACCCTGGTACGTCCGAAACCGCTGCTTCTGAAACTGC<br>TGAAGAGCGTGGGGGCGCAGAAAGATACCTACACCATGAAAGAAGTTCTGTTCTACCTGGGCCA<br>GTATATCATGACCAAGCGCCTGTATGACGAAAAACAGCAACACATTGTTTACTGCTCTAACGACCT<br>GCTGGGTGACCTGTTTGGTGTTCCTTCTTTCTCCGTTAAAGAACACCGCAAAATCTATACCATGAT<br>CTACCGTAACCTGGTGGTAGTTAACCAGCAAGAATCTAGCGATTCCGGTACTAGCGTCAGCGAGA<br>AC	IDT; adapted from [11]
pheS	His6-pheS	ATGAAGATTGAAGAAGGCAAACTGGTCAGCggcgaaaacctgtatttCcagttcttGttCgttcaagagttggtggcgagtg<br>cccatTCACATCTCGCAGAACTGGTTGCCAGTGCGAAGGCGGCCATTAGCCAGGCGTCAGATGTTG<br>CCGCGTTAGATAATGTGCGCGTCGAATATTTGGGTAAAAAAGGGCACTTAACCCTTCAGATGACG<br>ACCCTGCGTGAGCTGCCGCCAGAAGAGCGTCCGGCAGCTGGTGCGGTTATCAACGAAGCGAAA<br>GAGCAGGTTCAGCAGGCGCTGAATGCGCGTAAAGCGGAACTGGAAAGCGCTGCACTGAATGCG<br>CGTCTGGCGGCGGAAACGATTGATGTCTCTCTGCCAGGTCGTCGCATTGAAAACGGCGGTCTGC<br>ATCCGGTTACCCGTACCATCGACCGTATCGAAAGTTTCTTCGGTGAGCTTGGCTTTACCGTGGCA<br>ACCGGGCCGGAAATCGAGGATGATTATCATAACTTCGATGCTCTGAACATTCCTGGTCACCACCC<br>GGCGCGCGCTGACCACGACACTTTCTGGTTTGACACCACCCGCCTGCTGCGTACCCAGACCTCT<br>GGCGTACAGATCCGCACCATGAAAGCCCAGCAGCCACCGATTCGTATCATCGCGCCTGGCCGTG<br>TTTATCGTAACGACTACGACCAGACTCACACGCCGATGTTCCATCAGATGGAAGGTCTGATTGTT<br>GATACCAACATCAGCTTTACCAACCTGAAAGGCACGCTGCACGACTTCCTGCGTAACTTCTTTGA<br>GGAAGATTTGCAGATTCGCTTCCGTCCTTCCTACTTCCCGTTTGCCGAACCTTCTGCAGAAGTGG<br>ACGTCATGGGTAAAAACGGTAAATGGCTGGAAGTGCTGGGCTGCGGGATGGTGCATCCGAACGT<br>GTTGCGTAACGTTGGCATCGACCCGGAAGTTTACTCTGGTTTCGGCTTCGGGATGGGGATGGAG<br>CGTCTGACTATGTTGCGTTACGGCGTCACCGACCTGCGTTCATTCTTCGAAAACGATCTGCGTTT<br>CCTCAAACAGTTTAAATAA	[3]
cat	Chloramphenicol acetyltransferase (translational fusion)	GAGAAAAAAATCACTGGATATACCACCGTTGATATATCCCAATGGCATCGTAAAGAACATTTTGAG<br>GCATTTCAGTCAGTTGCTCAATGTACCTATAACCAGACCGTTCAGCTGGATATTACGGCCTTTTTA<br>AAGACCGTAAAGAAAAATAAGCACAAGTTTTATCCGGCCTTTATTCACATTCTTGCCCGCCTGATG<br>AATGCTCATCCGGAATTTCGTATGGCAATGAAAGACGGTGAGCTGGTGATATGGGATAGTGTTCA<br>CCCTTGTTACACCGTTTTCCATGAGCAAACTGAAACGTTTTCATCGCTCTGGAGTGAATACCACGA<br>CGATTTCCGGCAGTTTCTACACATATATTCGCAAGATGTGGCGTGTTACGGTGAAAACCTGGCCT<br>ATTTCCCTAAAGGGTTTATTGAGAATATGTTTTTCGTCTCAGCCAATCCCTGGGTGAGTTTCACCA<br>GTTTTGATTTAAACGTGGCCAATATGGACAACTTCTTCGCCCCCGTTTTCACCATGGGCAAATATT<br>ATACGCAAGGCGACAAGGTGCTGATGCCGCTGGCGATTCAGGTTCATCATGCCGTTTGTGATGG<br>CTTCCATGTCGGCAGAATGCTTAATGAATTACAACAGTACTGCGATGAGTGGCAGGGCGGGGCG<br>TAA	[3]
papB	RiPP modifying enzyme	ATGGCAAACCTGATCCAGGACCGCGAGGACGAACTGATTCATTTCCATCCGTACAAACTGTTCGA<br>GGTGGATTCAAAAACCTTCTTCTATAACGTAGTCACCAACGCGATTTTTGAAATTGATAGCCTGAT<br>AATCGACATTCTTCACTCAAAAGGTAAAAATGAGGAGCACGTTGTGAAAGATTTGGCTGAACGCTA<br>TGAGCTGTCTCAGGTTCGCGAAGCGATCCAGAACATGAAAGAGGCATACATTATAGCAACCGATG<br>CTAACATCTCCGACGTAGAGAAGATGGGTATCTTAGATAACTCGCAGCGCGTTTTTAAACTGTCTA<br>GCCTGACGCTCTTTATGGTGCAGGAATGCAACCTGCGGTGTACGTATTGTTACGGCGAAGAAGG<br>AGAATACAACCAGAAAGGTAAAATGACGTCCGAAATCGCCCGGAGCGCAGTGGATTTTCTGATTC<br>AACAGAGTGGTGAAATCGAACAGTTGAACATCACATTCTTTGGAGGCGAACCGCTGCTCAACTTT<br>CCATTAATACAAGAAACCGTGCAGTATGTGCACGAACAGAGCGAGATCCATAACAAGAAATTTAG<br>CTTTTCCATCACCACCAATGGCACGCTCATTACCCCCAAAATCAAAAACTTCTTCTATAAACACCA<br>CTTTGCAGTCCAGACTTCTATCGATGGTGATGAAAAGACGCACAATTTCAATCGCTTCTTCAAAGG<br>AGGCCAGGGCTCTTATGATCTGCTGTTAAAGCGGACGGAAGAAATGCGCAATGACCGTAAAATTG<br>GTGCACGTGGAACCGTGACCCCTGCCGAGCTGGACCTCTCAAAATCATTTGACCACTTAGTTAAA	

		CTCGGCTTTCGCAAAATCTACTTATCACCCGCTTTATATAGTCTCTCTGACGATCACTACGACACC<br>CTGAGCAAAGAGATGGTCAAACTTGTTGAACAATTCCGTGAGCTGCTGGAGCGTGAAGATTACGT<br>CACCGCGAAGAAAATGTCTAATGTTCTGGGTATGTTATCGAAGATTCACTCCGGTGGCCCGCGCA<br>TTCATTTTTGCGGTGCCGGCACTAATGCTGCCGCTGTCGATGTCCGCGGCAACCTTTTCCCGTGT<br>CATCGTTTCGTGGGTGAAGATGAATGTTCAATCGGTAACCTGTTCGACGAGGACCCGCTGTCAAA<br>ACAGTACAACTTTATAGAGAATTCTACAGTACGCAACCGTACTACGTGTTCGAAATGCTGGGCGAA<br>GAATCTGTGCGGCGGTGGTTGTCACCAAGAAAATTTCGCCGAGAATGGTAATGTGAACCAGCCA<br>GTGGGCAAATTATGCAAAGTGACCAAAAACTTCATCAACGCGACCATCAATCTGTACTTGCAACTT<br>ACTCAAGAACAACGCAGCATTCTGTTCGGC	
pap2c_1	Selection hit peptide sequence	ATGCTGAAGCAGATTAACGTTATTGCCGGAGTTAAAGAACCCATACGCGCGTACGTGTGTAAATA<br>TGGTGAATGGTGTGAGATCGTCGAAATCCGA	This work
amk1057	Fusion peptide for expression and purification	ATGCTGAAGCAGATTAACGTTATTGCCGGAGTTAAAGAACCCATACGCGCGTACGAAAACCTGTA<br>CTTTCAGGGTGTGTGTAAATATGGTGAATGGTGTGAGATCGTCGAAATC	This work

Plasmid origins

Name	Details	Sequence	Source[a]
pSC101	var2 – maintains at p15A-level copy number	AGTAAGACGGGTAAGCCTGTTGATGATACCGCTGCCTTACTGGGTGCATTAGCCAGTCTGAATGA<br>CCTGTCACGGGATAATCCGAAGTGGTCAGACTGGAAAATCAGAGGGCAGGAACTGCTGAACAGC<br>AAAAAGTCAGATAGCACCACATAGCAGACCCGCCATAAAACGCCCTGAGAAGCCCGTGACGGGC<br>TTTTCTTGTATTATGGGTAGTTTCCTTGCATGAATCCATAAAAGGCGCCTGTAGTGCCATTTACCC<br>CCATTCACTGCCAGAGCCGTGAGCGCAGCGAACTGAATGTCACGAAAAAGACAGCGACTCAGGT<br>GCCTGATGGTCGGAGACAAAAGGAATATTCAGCGATTTGCCCGAGCTTGCGAGGGTGCTACTTAA<br>GCCTTTAGGGTTTTAAGGTCTGTTTTGTAGAGGAGCAAACAGCGTTTGCGACATCCTTTTGTAATA<br>CTGCGGAACTGACTAAAGTAGTGAGTTATACACAGGGCTGGGATCTATTCTTTTTATCTTTTTTTAT<br>TCTTTCTTTATTCTATAAATTATAACCACTTGAATATAAACAAAAAAAACACACAAAGGTCTAGCGG<br>AATTTACAGAGGGTCTAGCAGAATTTACAAGTTTTCCAGCAAAGGTCTAGCAGAATTTACAGATAC<br>CCACAACTCAAAGGAAAAGGACTAGTAATTATCATTGACTAGCCCATCTCAATTGGTATAGTGATT<br>AAAATCACCTAGACCAATTGAGATGTATGTCTGAATTAGTTGTTTTCAAAGCAAATGAACTAGCGAT<br>TAGTCGCTATGACTTAACGGAGCATGAAACCAAGCTAATTTTATGCTGTGTGGCACTACTCAACCC<br>CACGATTGAAAACCCTACAAGGAAAGAACGGACGGTATCGTTCACTTATAACCAATACGCTCAGA<br>TGATGAACATCAGTAGGGAAAATGCTTATGGTGTATTAGCTAAAGCAACCAGAGAGCTGATGACG<br>AGAACTGTGGAAATCAGGAATCCTTTGGTTAAAGGCTTTTGGATTTTCCAGTGGACAAACTATGCC<br>AAGTTCTCAAGCGAAAAATTAGAATTAGTTTTTAGTGAAGAGATATTGCCTTATCTTTTCCAGTTAA<br>AAAAATTCATAAAATATAATCTGGAACATGTTAAGTCTTTTGAAAACAAATACTCTATGAGGATTTAT<br>GAGTGGTTATTAAAAGAACTAACACAAAAGAAAACTCACAAGGCAAATATAGAGATTAGCCTTGAT<br>GAATTTAAGTTCATGTTAATGCTTGAAAATAACTACCATGAGTTTAAAAGGCTTAACCAATGGGTTT<br>TGAAACCAATAAGTAAAGATTTAAACACTTACAGCAATATGAAATTGGTGGTTGATAAGCGAGGCC<br>GCCCGACTGATACGTTGATTTTCCAAGTTGAACTAGATAGACAAATGGATCTCGTAACCGAACTTG<br>AGAACAACCAGATAAAAATGAATGGTGACAAAATACCAACAACCATTACATCAGATTCCTACCTAC<br>ATAACGGACTAAGAAAAACACTACACGATGCTTTAACTGCAAAAATTCAGCTCACCAGTTTTGAGG<br>CAAAATTTTTGAGTGACATGCAAAGTAAGTATGATCTCAATGGTTCGTTCTCATGGCTCACGCAAA<br>AACAACGAACCACACTAGAGAACATACTGGCTAAATACGGAAGGATCTGAGGTTCTTATGGCTCT<br>TGTATCTATCAGTGAAGCATCAAGACTAACAAACAAAAGTAGAACAACTGTTCACCGTTACATATC<br>AAAGGGAAAACTGTCCATATGCACAGATGAAAACGGTGTAAAAAAGATAGATACATCAGAGCTTTT<br>ACGAGTTTTTGGTGCATTCAAAGCTGTTCACCATGAACAGATCGACAATGTAACAGATGAACAGCA<br>TGTAACACCTAATAGAACAGGTGAAACCAGTAAAACAAAGCAACTAGAACATGAAATTGAACACCT<br>GAGACAACTTGTTACAGCTCAACAGTCACACATAGACAGCCTGAAACAGGCGATGCTGCTTATCG<br>AATCAAAGCTGCCGACAACACGGGAGCCAGTGACGCCTCCCGTGGGGAAAAAATCATGGCAATT<br>CTGGAAGAAATAGCGCTTTCAGCCGGCAAACCGGCTGAAGCCGGATCTGCGATTCTGATAACAAA<br>CTAGCAACACCAGAACAGCCCGTTTGCGGGCAGCAAAACCCGTAC	[12]
ColE1	var2 – maintains at p15A-level copy number	TCATGACCAAAATCCCTTAACGTGAGTTTTCGTTCCACTGAGCGTCAGACCCCGTAGAAAAGATCA<br>AAGGATCTTCTTGAGATCCTTTTTTTCTGCGCGTAATCTGCTGCTTGCAAACAAAAAAACCACCGC<br>TACCAACGGTGGTTTGTTTGCCGGATCAAGAGCTACCAACTCTTTTTCCGAAGGTAACTGGCTTCA<br>GCAGAGCGCAGATACCAAATACTGTCCTTCTAGTGTAGCCGTAGTTAGGCCACCACTTCAAGAAC<br>TCTGTAGCACCGCCTACATACCTCGCTCTGCTAATCCTGTTACCAGTGGCTGCTGCCAGTGGCGA<br>TAAGTCGTGTCTTACCGGGTTGGACTCAAGACGATAGTTACCGGATAAGGCGCAGCGGTCGGGC<br>TGAACGGGGGGTTCGTGCACACAGCCCAGCTTGGAGCGAACGACCTACACCGAACTGAGATACC<br>TACAGCGTGAGCTATGAGAAAGCGCCACGCTTCCCGAAGGGAGAAAGGCGGACAGGTATCCGGT<br>AAGCGGCAGGGTCGGAACAGGAGAGCGCACGAGGGAGCTTCCAGGGGGAAACGCCTGGTATCT<br>TTATAGTCCTGTCGGGTTTCGCCACCTCTGACTTGAGCGTCGATTTTTGTGATGCTCGTCAGGGG<br>GGCGGAGCCTATGGAAAAACGCCAGCAACGCGGCCTTTTTACGGTTCCTGGCCTTTTGCTGGCC<br>TTTTGCTCACATGTTCTTTCCTGCGTTATCCCCTGATTCTGTGGATAACCGT	
p15A		TTAATAAGATGATCTTCTTGAGATCGTTTTGGTCTGCGCGTAATCTCTTGCTCTGAAAACGAAAAAA<br>CCGCCTTGCAGGGCGGTTTTTCGAAGGTTCTCTGAGCTACCAACTCTTTGAACCGAGGTAACTGG<br>CTTGGAGGAGCGCAGTCACCAAAACTTGTCCTTTCAGTTTAGCCTTAACCGGCGCATGACTTCAA<br>GACTAACTCCTCTAAATCAATTACCAGTGGCTGCTGCCAGTGGTGCTTTTGCATGTCTTTCCGGGT<br>TGGACTCAAGACGATAGTTACCGGATAAGGCGCAGCGGTCGGACTGAACGGGGGGTTCGTGCAT<br>ACAGTCCAGCTTGGAGCGAACTGCCTACCCGGAACTGAGTGTCAGGCGTGGAATGAGACAAACG<br>CGGCCATAACAGCGGAATGACACCGGTAAACCGAAAGGCAGGAACAGGAGAGCGCACGAGGGA<br>GCCGCCAGGGGAAACGCCTGGTATCTTTATAGTCCTGTCGGGTTTCGCCACCACTGATTTGAGC<br>GTCAGATTTCGTGATGCTTGTCAGGGGGGCGGAGCCTATGGAAAAACGGCTTTGCCGCGGCCCT<br>CTCACTTCCCTGTTAAGTATCTTCCTGGCATCTTCCAGGAAATCTCCGCCCCGTTCGTAAGCCATT<br>TCCGCTCGCCGCAGTCGAACGACCGAGCGTAGCGAGTCAGTGAGCGAGGAAGCGGAATATATC<br>CTGTATCACATATTCTGCTGACGCACCGGTGCAGCCTTTTTTCTCCTGCCACATGAAGCACTTCAC<br>TGACACCCTCATCAGTGCCAACATAGTAAGCCAGTATACACTCCGCTA	Unknown

Supplementary Table 2: Plasmid Sequences

Name	Description	Sequence
pTHSSe_44	Peptide cloning plasmid	AGTAAGACGGGTAAGCCTGTTGATGATACCGCTGCCTTACTGGGTGCATTAGCCAGTCTGAATGACCTGTCACGGGATAATCCG<br>AAGTGGTCAGACTGGAAAATCAGAGGGCAGGAACTGCTGAACAGCAAAAAGTCAGATAGCACCACATAGCAGACCCGCCATAAA<br>ACGCCCTGAGAAGCCCGTGACGGGCTTTTCTTGTATTATGGGTAGTTTCCTTGCATGAATCCATAAAAGGCGCCTGTAGTGCCAT<br>TTACCCCCATTCACTGCCAGAGCCGTGAGCGCAGCGAACTGAATGTCACGAAAAAGACAGCGACTCAGGTGCCTGATGGTCGG<br>AGACAAAAGGAATATTCAGCGATTTGCCCGAGCTTGCGAGGGTGCTACTTAAGCCTTTAGGGTTTTAAGGTCTGTTTTGTAGAGG<br>AGCAAACAGCGTTTGCGACATCCTTTTGTAATACTGCGGAACTGACTAAAGTAGTGAGTTATACACAGGGCTGGGATCTATTCTT<br>TTTATCTTTTTTTATTCTTTCTTTATTCTATAAATTATAACCACTTGAATATAAACAAAAAAAACACACAAAGGTCTAGCGGAATTTAC<br>AGAGGGTCTAGCAGAATTTACAAGTTTTCCAGCAAAGGTCTAGCAGAATTTACAGATACCCACAACTCAAAGGAAAAGGACTAGT<br>AATTATCATTGACTAGCCCATCTCAATTGGTATAGTGATTAAAATCACCTAGACCAATTGAGATGTATGTCTGAATTAGTTGTTTTC<br>AAAGCAAATGAACTAGCGATTAGTCGCTATGACTTAACGGAGCATGAAACCAAGCTAATTTTATGCTGTGTGGCACTACTCAACC<br>CCACGATTGAAAACCCTACAAGGAAAGAACGGACGGTATCGTTCACTTATAACCAATACGCTCAGATGATGAACATCAGTAGGGA<br>AAATGCTTATGGTGTATTAGCTAAAGCAACCAGAGAGCTGATGACGAGAACTGTGGAAATCAGGAATCCTTTGGTTAAAGGCTTT<br>TGGATTTTCCAGTGGACAAACTATGCCAAGTTCTCAAGCGAAAAATTAGAATTAGTTTTTAGTGAAGAGATATTGCCTTATCTTTTC<br>CAGTTAAAAAAATTCATAAAATATAATCTGGAACATGTTAAGTCTTTTGAAAACAAATACTCTATGAGGATTTATGAGTGGTTATTAA<br>AAGAACTAACACAAAAGAAAACTCACAAGGCAAATATAGAGATTAGCCTTGATGAATTTAAGTTCATGTTAATGCTTGAAAATAACT<br>ACCATGAGTTTAAAAGGCTTAACCAATGGGTTTTGAAACCAATAAGTAAAGATTTAAACACTTACAGCAATATGAAATTGGTGGTT<br>GATAAGCGAGGCCGCCCGACTGATACGTTGATTTTCCAAGTTGAACTAGATAGACAAATGGATCTCGTAACCGAACTTGAGAACA<br>ACCAGATAAAAATGAATGGTGACAAAATACCAACAACCATTACATCAGATTCCTACCTACATAACGGACTAAGAAAAACACTACAC<br>GATGCTTTAACTGCAAAAATTCAGCTCACCAGTTTTGAGGCAAAATTTTTGAGTGACATGCAAAGTAAGTATGATCTCAATGGTTC<br>GTTCTCATGGCTCACGCAAAAACAACGAACCACACTAGAGAACATACTGGCTAAATACGGAAGGATCTGAGGTTCTTATGGCTCT<br>TGTATCTATCAGTGAAGCATCAAGACTAACAAACAAAAGTAGAACAACTGTTCACCGTTACATATCAAAGGGAAAACTGTCCATAT<br>GCACAGATGAAAACGGTGTAAAAAAGATAGATACATCAGAGCTTTTACGAGTTTTTGGTGCATTCAAAGCTGTTCACCATGAACA<br>GATCGACAATGTAACAGATGAACAGCATGTAACACCTAATAGAACAGGTGAAACCAGTAAAACAAAGCAACTAGAACATGAAATT<br>GAACACCTGAGACAACTTGTTACAGCTCAACAGTCACACATAGACAGCCTGAAACAGGCGATGCTGCTTATCGAATCAAAGCTGC<br>CGACAACACGGGAGCCAGTGACGCCTCCCGTGGGGAAAAAATCATGGCAATTCTGGAAGAAATAGCGCTTTCAGCCGGCAAAC<br>CGGCTGAAGCCGGATCTGCGATTCTGATAACAAACTAGCAACACCAGAACAGCCCGTTTGCGGGCAGCAAAACCCGTACCGATT<br>ATCAAAAAGGATCTTCACCTAGATCCTTTTAAATTAAAAATGAAGTTTTAAATCAATCTAAAGTATATATGAGTAAACTTGGTCTGAC<br>AGTTACCAATGCTTAATCAGTGAGGCACCTATCTCAGCGATCTGTCTATTTCGTTCATCCATAGTTGCCTGACTCCCCGTCGTGTA<br>GATAACTACGATACGGGAGGGCTTACCATCTGGCCCCAGTGCTGCAATGATACCGCGAGAACCACGCTCACCGGCTCCAGATTT<br>ATCAGCAATAAACCAGCCAGCCGGAAGGGCCGAGCGCAGAAGTGGTCCTGCAACTTTATCCGCCTCCATCCAGTCTATTAATTG<br>TTGCCGGGAAGCTAGAGTAAGTAGTTCGCCAGTTAATAGTTTGCGCAACGTTGTTGCCATTGCTACAGGCATCGTGGTGTCACG<br>CTCGTCGTTTGGTATGGCTTCATTCAGCTCCGGTTCCCAACGATCAAGGCGAGTTACATGATCCCCCATGTTGTGCAAAAAAGCG<br>GTTAGCTCCTTCGGTCCTCCGATCGTTGTCAGAAGTAAGTTGGCCGCAGTGTTATCACTCATGGTTATGGCAGCACTGCATAATT<br>CTCTTACTGTCATGCCATCCGTAAGATGCTTTTCTGTGACTGGTGAGTACTCAACCAAGTCATTCTGAGAATAGTGTATGCGGCG<br>ACCGAGTTGCTCTTGCCCGGCGTCAATACGGGATAATACCGCGCCACATAGCAGAACTTTAAAAGTGCTCATCATTGGAAAACGT<br>TCTTCGGGGCGAAAACTCTCAAGGATCTTACCGCTGTTGAGATCCAGTTCGATATAACCCACTCGTGCACCCAACTGATCTTCAG<br>CATCTTTTACTTTCACCAGCGTTTCTGGGTGAGCAAAAACAGGAAGGCAAAATGCCGCAAAAAAGGGAATAAGGGCGACACGGA<br>AATGTTGAATACTCATACTCTTCCTTTTTCAATATTATTGAAGCATTTATCAGGGTTATTGTCTCATGAGCGGATACATATTTGAATG<br>TATTTAGAAAAATAAACAAATAGGGGTTCCGCGCACATTTCCCCGAAAAGTGCCACCTGACGTCTAAGAAACCATTATTATCATGA<br>CATTAACCTATAAAAATAGGCGTATCACGAGGCAGAATTTCAGATAAAAAAAATCCTTAGCTTTCGCTAAGGATGATTTCTGGAAT<br>TCGCGGCCGCTTCTAGAGGGAGGTGTCTTCGGCGCGCCTTTACGGCTAGCTCAGTCCTAGGTACTATGCTAGCAAGGTAGCTG<br>TCACCGGATGTGCTTTCCGGTCTGATGAGTCCGTGAGGACGAAACAGCCTCTACAAATAATTTTGTTTAATCACACAGGAAAGTA<br>CTAGATGAGCAAAGGAGAAGAACTTTTCACTGGAGTTGTCCCAATTCTTGTTGAATTAGATGGTGATGTTAATGGGCACAAATTTT<br>CTGTCCGTGGAGAGGGTGAAGGTGATGCTACAAACGGAAAACTCACCCTTAAATTTATTTGCACTACTGGAAAACTACCTGTTCC<br>GTGGCCAACACTTGTCACTACTCTGACCTATGGTGTTCAATGCTTTTCCCGTTATCCGGATCACATGAAACGGCATGACTTTTTCA<br>AGAGTGCCATGCCCGAAGGTTATGTACAGGAACGCACTATATCTTTCAAAGATGACGGGACCTACAAGACGCGTGCTGAAGTCA<br>AGTTTGAAGGTGATACCCTTGTTAATCGTATCGAGTTAAAGGGTATTGATTTTAAAGAAGATGGAAACATTCTTGGACACAAACTC<br>GAGTACAACTTTAACTCACACAATGTATACATCACGGCAGACAAACAAAAGAATGGAATCAAAGCTAACTTCAAAATTCGCCACAA<br>CGTTGAAGATGGTTCCGTTCAACTAGCAGACCATTATCAACAAAATACTCCAATTGGCGATGGCCCTGTCCTTTTACCAGACAAC<br>CATTACCTGTCGACACAATCTGTCCTTTCGAAAGATCCCAACGAAAAGCGTGACCACATGGTCCTTCTTGAGTTTGTAACTGCTG<br>CTGGGATTACACATGGCATGGATGAGCTCTACAAATAATACTAGAGCCAGGCATCAAATAAAACGAAAGGCTCAGTCGAAAGACT<br>GGGCCTTTCGTTTTATCTGTTGTTTGTCGGTGAACGCTCTCTACTAGAGTCACACTGGCTCACCTTCGGGTGGGCCTTTCTGCGT<br>TTATAGAAGACATCGCTTACTAGTAGCGGCCGCTGCAGTCCGGCAAAAAAGGGCAAGGTGTCACCACCCTGCCCTTTTTCTTTAA<br>AACCGAAAAGATTACTTCGCGTTATGCAGGCTTCCTCGCTCACTGACTCGCTGCGCTCGGTCGTTCGGCTGCGGCGAGCGGTAT<br>CAGCTCACTCAAAGGCGGTAATGAC
pTHSS-1458	Bait cloning plasmid	TCATGACCAAAATCCCTTAACGTGAGTTTTCGTTCCACTGAGCGTCAGACCCCGTAGAAAAGATCAAAGGATCTTCTTGAGATCC<br>TTTTTTTCTGCGCGTAATCTGCTGCTTGCAAACAAAAAAACCACCGCTACCAACGGTGGTTTGTTTGCCGGATCAAGAGCTACCA<br>ACTCTTTTTCCGAAGGTAACTGGCTTCAGCAGAGCGCAGATACCAAATACTGTCCTTCTAGTGTAGCCGTAGTTAGGCCACCACT<br>TCAAGAACTCTGTAGCACCGCCTACATACCTCGCTCTGCTAATCCTGTTACCAGTGGCTGCTGCCAGTGGCGATAAGTCGTGTCT<br>TACCGGGTTGGACTCAAGACGATAGTTACCGGATAAGGCGCAGCGGTCGGGCTGAACGGGGGGTTCGTGCACACAGCCCAGC<br>TTGGAGCGAACGACCTACACCGAACTGAGATACCTACAGCGTGAGCTATGAGAAAGCGCCACGCTTCCCGAAGGGAGAAAGGC<br>GGACAGGTATCCGGTAAGCGGCAGGGTCGGAACAGGAGAGCGCACGAGGGAGCTTCCAGGGGGAAACGCCTGGTATCTTTAT<br>AGTCCTGTCGGGTTTCGCCACCTCTGACTTGAGCGTCGATTTTTGTGATGCTCGTCAGGGGGGCGGAGCCTATGGAAAAACGCC<br>AGCAACGCGGCCTTTTTACGGTTCCTGGCCTTTTGCTGGCCTTTTGCTCACATGTTCTTTCCTGCGTTATCCCCTGATTCTGTGG<br>ATAACCGTCGATTATCAAAAAGGATCTTCACCTAGATCCTTTTAAATTAAAAATGAAGTTTTAAATCAATCTAAAGTATATATGAGTA<br>AACTTGGTCTGACAGTTATCAGAAGAACTCGTCAAGAAGGCGATAGAAGGCGATGCGCTGCGAATCGGGAGCGGCGATACCGT<br>AAAGCACGAGGAAGCGGTCAGCCCATTCGCCGCCAAGCTCTTCAGCAATATCACGGGTAGCCAACGCTATGTCCTGATAGCGG

		TCCGCCACACCCAGCCGGCCACAGTCGATGAATCCAGAAAAGCGGCCATTTTCCACCATGATATTCGGCAAGCAGGCATCGCCA<br>TGGGTCACGACGAGATCCTCGCCGTCGGGCATGCGCGCCTTGAGCCTGGCGAACAGTTCGGCTGGCGCGAGCCCCTGATGCT<br>CTTCGTCCAGATCATCCTGATCGACAAGACCGGCTTCCATCCGAGTACGTGCTCGCTCGATGCGATGTTTCGCTTGGTGGTCGA<br>ATGGGCAGGTAGCCGGATCAAGCGTATGCAGCCGCCGCATTGCATCAGCCATGATGGATACTTTCTCGGCAGGAGCAAGGTGA<br>GATGACAGGAGATCCTGCCCCGGCACTTCGCCCAATAGCAGCCAGTCCCTTCCCGCTTCAGTGACAACGTCGAGCACAGCTGC<br>GCAAGGAACGCCCGTCGTGGCCAGCCACGATAGCCGCGCTGCCTCGTCCTGCAGTTCATTCAGGGCACCGGACAGGTCGGTC<br>TTGACAAAAAGAACCGGGCGCCCCTGCGCTGACAGCCGGAACACGGCGGCATCAGAGCAGCCGATTGTCTGTTGTGCCCAGTC<br>ATAGCCGAATAGCCTCTCCACCCAAGCGGCCGGAGAACCTGCGTGCAATCCATCTTGTTCAATCATGCGAAACGATCCTCAATAT<br>TATTGAAGCATTTATCAGGGTTATTGTCTCATGAGCGGATACATATTTGAATGTATTTAGAAAAATAAACAAATAGGGGTTCCGCG<br>CACATTTCCCCGAAAAGTGCCACCTGACGTCTAAGAAACCATTATTATCATGACATTAACCTATAAAAATAGGCGTATCACGAGGC<br>AGAATTTCAGATAAAAAAAATCCTTAGCTTTCGCTAAGGATGATTTCTGGAATTCGCGGCCGCTTCTAGAGGGAGGTGTCTTCGG<br>CGCGCCTTTACGGCTAGCTCAGTCCTAGGTACTATGCTAGCAAGGTAGCTGTCACCGGATGTGCTTTCCGGTCTGATGAGTCCG<br>TGAGGACGAAACAGCCTCTACAAATAATTTTGTTTAATCACACAGGAAAGTACTAGATGAGCAAAGGAGAAGAACTTTTCACTGG<br>AGTTGTCCCAATTCTTGTTGAATTAGATGGTGATGTTAATGGGCACAAATTTTCTGTCCGTGGAGAGGGTGAAGGTGATGCTACA<br>AACGGAAAACTCACCCTTAAATTTATTTGCACTACTGGAAAACTACCTGTTCCGTGGCCAACACTTGTCACTACTCTGACCTATGG<br>TGTTCAATGCTTTTCCCGTTATCCGGATCACATGAAACGGCATGACTTTTTCAAGAGTGCCATGCCCGAAGGTTATGTACAGGAA<br>CGCACTATATCTTTCAAAGATGACGGGACCTACAAGACGCGTGCTGAAGTCAAGTTTGAAGGTGATACCCTTGTTAATCGTATCG<br>AGTTAAAGGGTATTGATTTTAAAGAAGATGGAAACATTCTTGGACACAAACTCGAGTACAACTTTAACTCACACAATGTATACATC<br>ACGGCAGACAAACAAAAGAATGGAATCAAAGCTAACTTCAAAATTCGCCACAACGTTGAAGATGGTTCCGTTCAACTAGCAGACC<br>ATTATCAACAAAATACTCCAATTGGCGATGGCCCTGTCCTTTTACCAGACAACCATTACCTGTCGACACAATCTGTCCTTTCGAAA<br>GATCCCAACGAAAAGCGTGACCACATGGTCCTTCTTGAGTTTGTAACTGCTGCTGGGATTACACATGGCATGGATGAGCTCTACA<br>AATAATACTAGAGCCAGGCATCAAATAAAACGAAAGGCTCAGTCGAAAGACTGGGCCTTTCGTTTTATCTGTTGTTTGTCGGTGA<br>ACGCTCTCTACTAGAGTCACACTGGCTCACCTTCGGGTGGGCCTTTCTGCGTTTATAGAAGACATCGCTTACTAGTAGCGGCCG<br>CTGCAGTCCGGCAAAAAAGGGCAAGGTGTCACCACCCTGCCCTTTTTCTTTAAAACCGAAAAGATTACTTCGCGTTATGCAGGCT<br>TCCTCGCTCACTGACTCGCTGCGCTCGGTCGTTCGGCTGCGGCGAGCGGTATCAGCTCACTCAAAGGCGGTAAT
pTHSS-1282	Pσ_sfGFP reporter	GATCCTTCCGTATTTAGCCAGTATGTTCTCTAGTGTGGTTCGTTGTTTTTGCGTGAGCCATGAGAACGAACCATTGAGATCATGCT<br>TACTTTGCATGTCACTCAAAAATTTTGCCTCAAAACTGGTGAGCTGAATTTTTGCAGTTAAAGCATCGTGTAGTGTTTTTCTTAGTC<br>CGTTACGTAGGTAGGAATCTGATGTAATGGTTGTTGGTATTTTGTCACCATTCATTTTTATCTGGTTGTTCTCAAGTTCGGTTACGA<br>GATCCATTTGTCTATCTAGTTCAACTTGGAAAATCAACGTATCAGTCGGGCGGCCTCGCTTATCAACCACCAATTTCATATTGCTG<br>TAAGTGTTTAAATCTTTACTTATTGGTTTCAAAACCCATTGGTTAAGCCTTTTAAACTCATGGTAGTTATTTTCAAGCATTAACATGA<br>ACTTAAATTCATCAAGGCTAATCTCTATATTTGCCTTGTGAGTTTTCTTTTGTGTTAGTTCTTTTAATAACCACTCATAAATCCTCAT<br>AGAGTATTTGTTTTCAAAAGACTTAACATGTTCCAGATTATATTTTATGAATTTTTTTAACTGGAAAAGATAAGGCAATATCTCTTCA<br>CTAAAAACTAATTCTAATTTTTCGCTTGAGAACTTGGCATAGTTTGTCCACTGGAAAATCTCAAAGCCTTTAACCAAAGGATTCCTG<br>ATTTCCACAGTTCTCGTCATCAGCTCTCTGGTTGCTTTAGCTAATACACCATAAGCATTTTCCCTACTGATGTTCATCATCTGAGC<br>GTATTGGTTATAAGTGAACGATACCGTCCGTTCTTTCCTTGTAGGGTTTTCAATCGTGGGGTTGAGTAGTGCCACACAGCATAAA<br>ATTAGCTTGGTTTCATGCTCCGTTAAGTCATAGCGACTAATCGCTAGTTCATTTGCTTTGAAAACAACTAATTCAGACATACATCTC<br>AATTGGTCTAGGTGATTTTAATCACTATACCAATTGAGATGGGCTAGTCAATGATAATTACTAGTCCTTTTCCTTTGAGTTGTGGGT<br>ATCTGTAAATTCTGCTAGACCTTTGCTGGAAAACTTGTAAATTCTGCTAGACCCTCTGTAAATTCCGCTAGACCTTTGTGTGTTTTT<br>TTTGTTTATATTCAAGTGGTTATAATTTATAGAATAAAGAAAGAATAAAAAAAGATAAAAAGAATAGATCCCAGCCCTGTGTATAAC<br>TCACTACTTTAGTCAGTTCCGCAGTATTACAAAAGGATGTCGCAAACGCTGTTTGCTCCTCTACAAAACAGACCTTAAAACCCTAA<br>AGGCTTAAGTAGCACCCTCGCAAGCTCGGGCAAATCGCTGAATATTCCTTTTGTCTCCGACCATCAGGCACCTGAGTCGCTGTC<br>TTTTTCGTGACATTCAGTTCGCTGCGCTCACGGCTCTGGCAGTGAATGGGGGTAAATGGCACTACAGGCGCCTTTTATGGATTCA<br>TGCAAGGAAACTACCCATAATACAAGAAAAGCCCGTCACGGGCTTCTCAGGGCGTTTTATGGCGGGTCTGCTATGTGGTGCTAT<br>CTGACTTTTTGCTGTTCAGCAGTTCCTGCCCTCTGATTTTCCAGTCTGACCACTTCGGATTATCCCGTGACAGGTCATTCAGACTG<br>GCTAATGCACCCAGTAAGGCAGCGGTATCATCAACAGGCTTACCCGTCTTACTGTCCCTAGTGCTTGGATTCTCACCAATAAAAA<br>ACGCCCGGCGGCAACCGAGCGTTCTGAACAAATCCAGATGGAGTTCTGAGGTCATTACTGGATCTATCAACAGGAGTCCAAGCG<br>AGCTCTCGAACCCCAGAGTCCCGCTTACCAATGCTTAATCAGTGAGGCACCTATCTCAGCGATCTGTCTATTTCGTTCATCCATA<br>GTTGCCTGACTCCCCGTCGTGTAGATAACTACGATACGGGAGGGCTTACCATCTGGCCCCAGTGCTGCAATGATACCGCGAGAC<br>CCACGCTCACCGGCTCCAGATTTATCAGCAATAAACCAGCCAGCCGGAAGGGCCGAGCGCAGAAGTGGTCCTGCAACTTTATC<br>CGCCTCCATCCAGTCTATTAATTGTTGCCGGGAAGCTAGAGTAAGTAGTTCGCCAGTTAATAGTTTGCGCAACGTTGTTGCCATT<br>GCTACAGGCATCGTGGTGTCACGCTCGTCGTTTGGTATGGCTTCATTCAGCTCCGGTTCCCAACGATCAAGGCGAGTTACATGA<br>TCCCCCATGTTGTGCAAAAAAGCGGTTAGCTCCTTCGGTCCTCCGATCGTTGTCAGAAGTAAGTTGGCCGCAGTGTTATCACTCA<br>TGGTTATGGCAGCACTGCATAATTCTCTTACTGTCATGCCATCCGTAAGATGCTTTTCTGTGACTGGTGAGTACTCAACCAAGTCA<br>TTCTGAGAATAGTGTATGCGGCGACCGAGTTGCTCTTGCCCGGCGTCAATACGGGATAATACCGCGCCACATAGCAGAACTTTA<br>AAAGTGCTCATCATTGGAAAACGTTCTTCGGGGCGAAAACTCTCAAGGATCTTACCGCTGTTGAGATCCAGTTCGATGTAACCCA<br>CTCGTGCACCCAACTGATCTTCAGCATCTTTTACTTTCACCAGCGTTTCTGGGTGAGCAAAAACAGGAAGGCAAAATGCCGCAAA<br>AAAGGGAATAAGGGCGACACGGAAATGTTGAATACTCATACTCTTCCTTTTTCAATATTATTGAAGCATTTATCGCGAAACGATCC<br>TCATCCTGTCTCTTGATCAGATCTTGATCCCCTGCGCCATCAGATCCTTGGCGGCAAGAAAGCCATCCAGTTTACTTTGCAGGGC<br>TTCCCAACCTTACCAGAGGGCGCCCCAGCTGGCAATTCCGACGTCTAAGAAACCATTATTATCATGACATTAACCTATAAAAATA<br>GGCGTATCACGAGGCCCTTTCGTCTTCACCTCGAGTGCCACCTGACGTCTAAGAAACCATTATTATCATGACATTAACCTATAAAA<br>ATAGGCGTATCACGAGGCAGAATTTCAGATAAAAAAAATCCTTAGCTTTCGCTAAGGATGATTTCTGGAATTCGCGGCCGCTTCT<br>AGAGGCGCGGATAAAAATTTCATTTGCCCGCGACGGATTCCCCGCCCATCTATCGTTGAACCCATCAGCTGCGTTCATCAGCGA<br>AGCTGTCACCGGATGTGCTTTCCGGTCTGATGAGTCCGTGAGGACGAAACAGCCTCTACAAATAATTTTGTTTAATCACACAGGA<br>AAGTACTAGATGAGCAAAGGAGAAGAACTTTTCACTGGAGTTGTCCCAATTCTTGTTGAATTAGATGGTGATGTTAATGGGCACA<br>AATTTTCTGTCCGTGGAGAGGGTGAAGGTGATGCTACAAACGGAAAACTCACCCTTAAATTTATTTGCACTACTGGAAAACTACCT<br>GTTCCGTGGCCAACACTTGTCACTACTCTGACCTATGGTGTTCAATGCTTTTCCCGTTATCCGGATCACATGAAACGGCATGACT<br>TTTTCAAGAGTGCCATGCCCGAAGGTTATGTACAGGAACGCACTATATCTTTCAAAGATGACGGGACCTACAAGACGCGTGCTGA<br>AGTCAAGTTTGAAGGTGATACCCTTGTTAATCGTATCGAGTTAAAGGGTATTGATTTTAAAGAAGATGGAAACATTCTTGGACACA<br>AACTCGAGTACAACTTTAACTCACACAATGTATACATCACGGCAGACAAACAAAAGAATGGAATCAAAGCTAACTTCAAAATTCGC<br>CACAACGTTGAAGATGGTTCCGTTCAACTAGCAGACCATTATCAACAAAATACTCCAATTGGCGATGGCCCTGTCCTTTTACCAG<br>ACAACCATTACCTGTCGACACAATCTGTCCTTTCGAAAGATCCCAACGAAAAGCGTGACCACATGGTCCTTCTTGAGTTTGTAACT<br>GCTGCTGGGATTACACATGGCATGGATGAGCTCTACAAATAATACTAGAGCCAGGCATCAAATAAAACGAAAGGCTCAGTCGAA<br>AGACTGGGCCTTTCGTTTTATCTGTTGTTTGTCGGTGAACGCTCTCTACTAGAGTCACACTGGCTCACCTTCGGGTGGGCCTTTC<br>TGCGTTTATATACTAGTAGCGGCCGCTGCAGTCCGGCAAAAAAGGGCAAGGTGTCACCACCCTGCCCTTTTTCTTTAAAACCGAA<br>AAGATTACTTCGCGTTATGCAGGCTTCCTCGCTCACTGACTCGCTGCGCTCGGTCGTTCGGCTGCGGCGAGCGGTATCAGCTCA<br>CTCAAAGGCGGTAATCTGTTGTTTGTCGGTGAACGCTCTCCTGAGTAGGACAAATCCGCCGCCCTAGACCTAGGGTACGGGTTT<br>TGCTGCCCGCAAACGGGCTGTTCTGGTGTTGCTAGTTTGTTATCAGAATCGCAGATCCGGCTTCAGGTTTGCCGGCTGAAAGCG<br>CTATTTCTTCCAGAATTGCCATGATTTTTTCCCCACGGGAGGCGTCACTGGCTCCCGTGTTGTCGGCAGCTTTGATTCGATAAGC<br>AGCATCGCCTGTTTCAGGCTGTCTATGTGTGACTGTTGAGCTGTAACAAGTTGTCTCAGGTGTTCAATTTCATGTTCTAGTTGCTT<br>TGTTTTACTGGTTTCACCTGTTCTATTAGGTGTTACATGCTGTTCATCTGTTACATTGTCGATCTGTTCATGGTGAACAGCTTTAAA<br>TGCACCAAAAACTCGTAAAAGCTCTGATGTATCTATCTTTTTTACACCGTTTTCATCTGTGCATATGGACAGTTTTCCCTTTGATAT<br>CTAACGGTGAACAGTTGTTCTACTTTTGTTTGTTAGTCTTGATGCTTCACTGATAGATACAAGAGCCATAAGAACCTCA
pDAA-558	Alternative library cloning plasmid	AGTAAGACGGGTAAGCCTGTTGATGATACCGCTGCCTTACTGGGTGCATTAGCCAGTCTGAATGACCTGTCACGGGATAATCCG<br>AAGTGGTCAGACTGGAAAATCAGAGGGCAGGAACTGCTGAACAGCAAAAAGTCAGATAGCACCACATAGCAGACCCGCCATAAA<br>ACGCCCTGAGAAGCCCGTGACGGGCTTTTCTTGTATTATGGGTAGTTTCCTTGCATGAATCCATAAAAGGCGCCTGTAGTGCCAT<br>TTACCCCCATTCACTGCCAGAGCCGTGAGCGCAGCGAACTGAATGTCACGAAAAAGACAGCGACTCAGGTGCCTGATGGTCGG<br>AGACAAAAGGAATATTCAGCGATTTGCCCGAGCTTGCGAGGGTGCTACTTAAGCCTTTAGGGTTTTAAGGTCTGTTTTGTAGAGG<br>AGCAAACAGCGTTTGCGACATCCTTTTGTAATACTGCGGAACTGACTAAAGTAGTGAGTTATACACAGGGCTGGGATCTATTCTT<br>TTTATCTTTTTTTATTCTTTCTTTATTCTATAAATTATAACCACTTGAATATAAACAAAAAAAACACACAAAGGTCTAGCGGAATTTAC<br>AGAGGGTCTAGCAGAATTTACAAGTTTTCCAGCAAAGGTCTAGCAGAATTTACAGATACCCACAACTCAAAGGAAAAGGACTAGT<br>AATTATCATTGACTAGCCCATCTCAATTGGTATAGTGATTAAAATCACCTAGACCAATTGAGATGTATGTCTGAATTAGTTGTTTTC

		AAAGCAAATGAACTAGCGATTAGTCGCTATGACTTAACGGAGCATGAAACCAAGCTAATTTTATGCTGTGTGGCACTACTCAACC<br>CCACGATTGAAAACCCTACAAGGAAAGAACGGACGGTATCGTTCACTTATAACCAATACGCTCAGATGATGAACATCAGTAGGGA<br>AAATGCTTATGGTGTATTAGCTAAAGCAACCAGAGAGCTGATGACGAGAACTGTGGAAATCAGGAATCCTTTGGTTAAAGGCTTT<br>TGGATTTTCCAGTGGACAAACTATGCCAAGTTCTCAAGCGAAAAATTAGAATTAGTTTTTAGTGAAGAGATATTGCCTTATCTTTTC<br>CAGTTAAAAAAATTCATAAAATATAATCTGGAACATGTTAAGTCTTTTGAAAACAAATACTCTATGAGGATTTATGAGTGGTTATTAA<br>AAGAACTAACACAAAAGAAAACTCACAAGGCAAATATAGAGATTAGCCTTGATGAATTTAAGTTCATGTTAATGCTTGAAAATAACT<br>ACCATGAGTTTAAAAGGCTTAACCAATGGGTTTTGAAACCAATAAGTAAAGATTTAAACACTTACAGCAATATGAAATTGGTGGTT<br>GATAAGCGAGGCCGCCCGACTGATACGTTGATTTTCCAAGTTGAACTAGATAGACAAATGGATCTCGTAACCGAACTTGAGAACA<br>ACCAGATAAAAATGAATGGTGACAAAATACCAACAACCATTACATCAGATTCCTACCTACATAACGGACTAAGAAAAACACTACAC<br>GATGCTTTAACTGCAAAAATTCAGCTCACCAGTTTTGAGGCAAAATTTTTGAGTGACATGCAAAGTAAGTATGATCTCAATGGTTC<br>GTTCTCATGGCTCACGCAAAAACAACGAACCACACTAGAGAACATACTGGCTAAATACGGAAGGATCTGAGGTTCTTATGGCTCT<br>TGTATCTATCAGTGAAGCATCAAGACTAACAAACAAAAGTAGAACAACTGTTCACCGTTACATATCAAAGGGAAAACTGTCCATAT<br>GCACAGATGAAAACGGTGTAAAAAAGATAGATACATCAGAGCTTTTACGAGTTTTTGGTGCATTCAAAGCTGTTCACCATGAACA<br>GATCGACAATGTAACAGATGAACAGCATGTAACACCTAATAGAACAGGTGAAACCAGTAAAACAAAGCAACTAGAACATGAAATT<br>GAACACCTGAGACAACTTGTTACAGCTCAACAGTCACACATAGACAGCCTGAAACAGGCGATGCTGCTTATCGAATCAAAGCTGC<br>CGACAACACGGGAGCCAGTGACGCCTCCCGTGGGGAAAAAATCATGGCAATTCTGGAAGAAATAGCGCTTTCAGCCGGCAAAC<br>CGGCTGAAGCCGGATCTGCGATTCTGATAACAAACTAGCAACACCAGAACAGCCCGTTTGCGGGCAGCAAAACCCGTACCGATT<br>ATCAAAAAGGATCTTCACCTAGATCCTTTTAAATTAAAAATGAAGTTTTAAATCAATCTAAAGTATATATGAGTAAACTTGGTCTGAC<br>AGTTACCAATGCTTAATCAGTGAGGCACCTATCTCAGCGATCTGTCTATTTCGTTCATCCATAGTTGCCTGACTCCCCGTCGTGTA<br>GATAACTACGATACGGGAGGGCTTACCATCTGGCCCCAGTGCTGCAATGATACCGCGAGAACCACGCTCACCGGCTCCAGATTT<br>ATCAGCAATAAACCAGCCAGCCGGAAGGGCCGAGCGCAGAAGTGGTCCTGCAACTTTATCCGCCTCCATCCAGTCTATTAATTG<br>TTGCCGGGAAGCTAGAGTAAGTAGTTCGCCAGTTAATAGTTTGCGCAACGTTGTTGCCATTGCTACAGGCATCGTGGTGTCACG<br>CTCGTCGTTTGGTATGGCTTCATTCAGCTCCGGTTCCCAACGATCAAGGCGAGTTACATGATCCCCCATGTTGTGCAAAAAAGCG<br>GTTAGCTCCTTCGGTCCTCCGATCGTTGTCAGAAGTAAGTTGGCCGCAGTGTTATCACTCATGGTTATGGCAGCACTGCATAATT<br>CTCTTACTGTCATGCCATCCGTAAGATGCTTTTCTGTGACTGGTGAGTACTCAACCAAGTCATTCTGAGAATAGTGTATGCGGCG<br>ACCGAGTTGCTCTTGCCCGGCGTCAATACGGGATAATACCGCGCCACATAGCAGAACTTTAAAAGTGCTCATCATTGGAAAACGT<br>TCTTCGGGGCGAAAACTCTCAAGGATCTTACCGCTGTTGAGATCCAGTTCGATATAACCCACTCGTGCACCCAACTGATCTTCAG<br>CATCTTTTACTTTCACCAGCGTTTCTGGGTGAGCAAAAACAGGAAGGCAAAATGCCGCAAAAAAGGGAATAAGGGCGACACGGA<br>AATGTTGAATACTCATACTCTTCCTTTTTCAATATTATTGAAGCATTTATCAGGGTTATTGTCTCATGAGCGGATACATATTTGAATG<br>TATTTAGAAAAATAAACAAATAGGGGTTCCGCGCACATTTCCCCGAAAAGTGCCACCTGACGTCTAAGAAACCATTATTATCATGA<br>CATTAACCTATAAAAATAGGCGTATCACGAGGCAGAATTTCAGATAAAAAAAATCCTTAGCTTTCGCTAAGGATGATTTCTGGAAT<br>TCGCGGCCGCTTCTAGAGGGAGCCAATTATTGAAGGCCTCCCTAACGGGGGGCCTTTTTTTGTTTCTGGTCTCCCGCTTAACGA<br>TCGTTGGCTGACCTGTAGGATCGTACAGGTTTACGCAAGAAAATGGTTTGTTACAGTCGAATAAAAGCTGTCACCGGATGTGCTT<br>TCCGGTCTGATGAGTCCGTGAGGACGAAACAGCCTCTACAAATAATTTTGTTTAATACTTGAGACGGGCGCGCCTTTACGGCTAG<br>CTCAGTCCTAGGTACTATGCTAGCAAGGTAGCTGTCACCGGATGTGCTTTCCGGTCTGATGAGTCCGTGAGGACGAAACAGCCT<br>CTACAAATAATTTTGTTTAATCACACAGGAAAGTACTAGATGAGCAAAGGAGAAGAACTTTTCACTGGAGTTGTCCCAATTCTTGT<br>TGAATTAGATGGTGATGTTAATGGGCACAAATTTTCTGTCCGTGGAGAGGGTGAAGGTGATGCTACAAACGGAAAACTCACCCTT<br>AAATTTATTTGCACTACTGGAAAACTACCTGTTCCGTGGCCAACACTTGTCACTACTCTGACCTATGGTGTTCAATGCTTTTCCCG<br>TTATCCGGATCACATGAAACGGCATGACTTTTTCAAGAGTGCCATGCCCGAAGGTTATGTACAGGAACGCACTATATCTTTCAAA<br>GATGACGGGACCTACAAGACGCGTGCTGAAGTCAAGTTTGAAGGTGATACCCTTGTTAATCGTATCGAGTTAAAGGGTATTGATT<br>TTAAAGAAGATGGAAACATTCTTGGACACAAACTCGAGTACAACTTTAACTCACACAATGTATACATCACGGCAGACAAACAAAAG<br>AATGGAATCAAAGCTAACTTCAAAATTCGCCACAACGTTGAAGATGGTTCCGTTCAACTAGCAGACCATTATCAACAAAATACTCC<br>AATTGGCGATGGCCCTGTCCTTTTACCAGACAACCATTACCTGTCGACACAATCTGTCCTTTCGAAAGATCCCAACGAAAAGCGT<br>GACCACATGGTCCTTCTTGAGTTTGTAACTGCTGCTGGGATTACACATGGCATGGATGAGCTCTACAAATAATACTAGAGCCAGG<br>CATCAAATAAAACGAAAGGCTCAGTCGAAAGACTGGGCCTTTCGTTTTATCTGTTGTTTGTCGGTGAACGCTCTCTACTAGAGTC<br>ACACTGGCTCACCTTCGGGTGGGCCTTTCTGCGTTTATACGTCTCACGAGGAGGTAAGGGAGGACCTGGAGGTCGGGGAGGTG<br>TTGGAGGTGGTGGAGGAATTGGAGGTGGTTTTATCGCTTCCAACTGCTGGCTGGATACCCGTCCGGCACCGGATGAACAGCTG<br>GAAGCAAGCGCACAGAGCCGTCGTATGGCACAGGCACTGGATCAGCTGCCGGATCGTCAGCGTGAAGCAATTGTTCTGCAGTA<br>TTATCAAGAACTGAGCAATACCGAAGCAGCAGCACTGATGCAAATTAGCGTTGAAGCCCTGGAAAGCCTGCTGAGCCGTGCACG<br>TCGTAATCTGCGTAGCCATCTGGCCGAAGCACCGGGTGCAGATCTGAGCGGTCGTCGCAAACCGTAAAGGTGATACTTTCAGCC<br>AAAAAACTTAAGACCGCCGGTCTTGTCCACTACCTTGCAGTAATGCGGTGGACAGGATCGGCGGTTTTCTTTTCTCTTCTCAAAG<br>ACCGTCCCGCTTACTAGTAGCGGCCGCTGCAGTCCGGCAAAAAAGGGCAAGGTGTCACCACCCTGCCCTTTTTCTTTAAAACCG<br>AAAAGATTACTTCGCGTTATGCAGGCTTCCTCGCTCACTGACTCGCTGCGCTCGGTCGTTCGGCTGCGGCGAGCGGTATCAGCT<br>CACTCAAAGGCGGTAATGAC
pAMK-267	P_{Lux}_*sfGFP*-Npu^{C}-σ^{C} cloning backbone	AGTAAGACGGGTAAGCCTGTTGATGATACCGCTGCCTTACTGGGTGCATTAGCCAGTCTGAATGACCTGTCACGGGATAATCCG<br>AAGTGGTCAGACTGGAAAATCAGAGGGCAGGAACTGCTGAACAGCAAAAAGTCAGATAGCACCACATAGCAGACCCGCCATAAA<br>ACGCCCTGAGAAGCCCGTGACGGGCTTTTCTTGTATTATGGGTAGTTTCCTTGCATGAATCCATAAAAGGCGCCTGTAGTGCCAT<br>TTACCCCCATTCACTGCCAGAGCCGTGAGCGCAGCGAACTGAATGTCACGAAAAAGACAGCGACTCAGGTGCCTGATGGTCGG<br>AGACAAAAGGAATATTCAGCGATTTGCCCGAGCTTGCGAGGGTGCTACTTAAGCCTTTAGGGTTTTAAGGTCTGTTTTGTAGAGG<br>AGCAAACAGCGTTTGCGACATCCTTTTGTAATACTGCGGAACTGACTAAAGTAGTGAGTTATACACAGGGCTGGGATCTATTCTT<br>TTTATCTTTTTTTATTCTTTCTTTATTCTATAAATTATAACCACTTGAATATAAACAAAAAAAACACACAAAGGTCTAGCGGAATTTAC<br>AGAGGGTCTAGCAGAATTTACAAGTTTTCCAGCAAAGGTCTAGCAGAATTTACAGATACCCACAACTCAAAGGAAAAGGACTAGT<br>AATTATCATTGACTAGCCCATCTCAATTGGTATAGTGATTAAAATCACCTAGACCAATTGAGATGTATGTCTGAATTAGTTGTTTTC<br>AAAGCAAATGAACTAGCGATTAGTCGCTATGACTTAACGGAGCATGAAACCAAGCTAATTTTATGCTGTGTGGCACTACTCAACC<br>CCACGATTGAAAACCCTACAAGGAAAGAACGGACGGTATCGTTCACTTATAACCAATACGCTCAGATGATGAACATCAGTAGGGA<br>AAATGCTTATGGTGTATTAGCTAAAGCAACCAGAGAGCTGATGACGAGAACTGTGGAAATCAGGAATCCTTTGGTTAAAGGCTTT<br>TGGATTTTCCAGTGGACAAACTATGCCAAGTTCTCAAGCGAAAAATTAGAATTAGTTTTTAGTGAAGAGATATTGCCTTATCTTTTC<br>CAGTTAAAAAAATTCATAAAATATAATCTGGAACATGTTAAGTCTTTTGAAAACAAATACTCTATGAGGATTTATGAGTGGTTATTAA<br>AAGAACTAACACAAAAGAAAACTCACAAGGCAAATATAGAGATTAGCCTTGATGAATTTAAGTTCATGTTAATGCTTGAAAATAACT<br>ACCATGAGTTTAAAAGGCTTAACCAATGGGTTTTGAAACCAATAAGTAAAGATTTAAACACTTACAGCAATATGAAATTGGTGGTT<br>GATAAGCGAGGCCGCCCGACTGATACGTTGATTTTCCAAGTTGAACTAGATAGACAAATGGATCTCGTAACCGAACTTGAGAACA<br>ACCAGATAAAAATGAATGGTGACAAAATACCAACAACCATTACATCAGATTCCTACCTACATAACGGACTAAGAAAAACACTACAC<br>GATGCTTTAACTGCAAAAATTCAGCTCACCAGTTTTGAGGCAAAATTTTTGAGTGACATGCAAAGTAAGTATGATCTCAATGGTTC<br>GTTCTCATGGCTCACGCAAAAACAACGAACCACACTAGAGAACATACTGGCTAAATACGGAAGGATCTGAGGTTCTTATGGCTCT<br>TGTATCTATCAGTGAAGCATCAAGACTAACAAACAAAAGTAGAACAACTGTTCACCGTTACATATCAAAGGGAAAACTGTCCATAT<br>GCACAGATGAAAACGGTGTAAAAAAGATAGATACATCAGAGCTTTTACGAGTTTTTGGTGCATTCAAAGCTGTTCACCATGAACA<br>GATCGACAATGTAACAGATGAACAGCATGTAACACCTAATAGAACAGGTGAAACCAGTAAAACAAAGCAACTAGAACATGAAATT<br>GAACACCTGAGACAACTTGTTACAGCTCAACAGTCACACATAGACAGCCTGAAACAGGCGATGCTGCTTATCGAATCAAAGCTGC<br>CGACAACACGGGAGCCAGTGACGCCTCCCGTGGGGAAAAAATCATGGCAATTCTGGAAGAAATAGCGCTTTCAGCCGGCAAAC<br>CGGCTGAAGCCGGATCTGCGATTCTGATAACAAACTAGCAACACCAGAACAGCCCGTTTGCGGGCAGCAAAACCCGTACCGATT<br>ATCAAAAAGGATCTTCACCTAGATCCTTTTAAATTAAAAATGAAGTTTTAAATCAATCTAAAGTATATATGAGTAAACTTGGTCTGAC<br>AGTTACCAATGCTTAATCAGTGAGGCACCTATCTCAGCGATCTGTCTATTTCGTTCATCCATAGTTGCCTGACTCCCCGTCGTGTA<br>GATAACTACGATACGGGAGGGCTTACCATCTGGCCCCAGTGCTGCAATGATACCGCGAGAACCACGCTCACCGGCTCCAGATTT<br>ATCAGCAATAAACCAGCCAGCCGGAAGGGCCGAGCGCAGAAGTGGTCCTGCAACTTTATCCGCCTCCATCCAGTCTATTAATTG<br>TTGCCGGGAAGCTAGAGTAAGTAGTTCGCCAGTTAATAGTTTGCGCAACGTTGTTGCCATTGCTACAGGCATCGTGGTGTCACG<br>CTCGTCGTTTGGTATGGCTTCATTCAGCTCCGGTTCCCAACGATCAAGGCGAGTTACATGATCCCCCATGTTGTGCAAAAAAGCG<br>GTTAGCTCCTTCGGTCCTCCGATCGTTGTCAGAAGTAAGTTGGCCGCAGTGTTATCACTCATGGTTATGGCAGCACTGCATAATT<br>CTCTTACTGTCATGCCATCCGTAAGATGCTTTTCTGTGACTGGTGAGTACTCAACCAAGTCATTCTGAGAATAGTGTATGCGGCG<br>ACCGAGTTGCTCTTGCCCGGCGTCAATACGGGATAATACCGCGCCACATAGCAGAACTTTAAAAGTGCTCATCATTGGAAAACGT<br>TCTTCGGGGCGAAAACTCTCAAGGATCTTACCGCTGTTGAGATCCAGTTCGATATAACCCACTCGTGCACCCAACTGATCTTCAG<br>CATCTTTTACTTTCACCAGCGTTTCTGGGTGAGCAAAAACAGGAAGGCAAAATGCCGCAAAAAAGGGAATAAGGGCGACACGGA<br>AATGTTGAATACTCATACTCTTCCTTTTTCAATATTATTGAAGCATTTATCAGGGTTATTGTCTCATGAGCGGATACATATTTGAATG

		TATTTAGAAAAATAAACAAATAGGGGTTCCGCGCACATTTCCCCGAAAAGTGCCACCTGACGTCTAAGAAGAATTCGCGGCCGCT<br>TCTAGAGGGAGCCAATTATTGAAGGCCTCCCTAACGGGGGGCCTTTTTTTGTTTCTGGTCTCCCGCTTAACGATCGTTGGCTGAC<br>CTGTAGGATCGTACAGGTTTACGCAAGAAAATGGTTTGTTACAGTCGAATAAAAGCTGTCACCGGATGTGCTTTCCGGTCTGATG<br>AGTCCGTGAGGACGAAACAGCCTCTACAAATAATTTTGTTTAATCCATCTCTATGGCGGATTTTATGTCATATTACCACCATCACC<br>ATCATCACATGGGAAGAGCTAGCAAAGGAGAAGAACTTTTCACTGGAGTTGTCCCAATTCTTGTTGAATTAGATGGTGATGTTAAT<br>GGGCACAAATTTTCTGTCCGTGGAGAGGGTGAAGGTGATGCTACAAACGGAAAACTCACCCTTAAATTTATTTGCACTACTGGAA<br>AACTACCTGTTCCGTGGCCAACACTTGTCACTACTCTGACCTATGGTGTTCAATGCTTTTCCCGTTATCCGGATCACATGAAACG<br>GCATGACTTTTTCAAGAGTGCCATGCCCGAAGGTTATGTACAGGAACGCACTATATCTTTCAAAGATGACGGGACCTACAAGACG<br>CGTGCTGAAGTCAAGTTTGAAGGTGATACCCTTGTTAATCGTATCGAGTTAAAGGGTATTGATTTTAAAGAAGATGGAAACATTCT<br>TGGACACAAACTCGAGTACAACTTTAACTCACACAATGTATACATCACGGCAGACAAACAAAAGAATGGAATCAAAGCTAACTTCA<br>AAATTCGCCACAACGTTGAAGATGGTTCCGTTCAACTAGCAGACCATTATCAACAAAATACTCCAATTGGCGATGGCCCTGTCCT<br>TTTACCAGACAACCATTACCTGTCGACACAATCTGTCCTTTCGAAAGATCCCAACGAAAAGCGTGACCACATGGTCCTTCTTGAG<br>TTTGTAACTGCTGCTGGGATTACACATGGCATGGATGAGCTCTACAAAGGCTCTTCACGAGGAGGTAAGGGAGGACCTGGAGGT<br>CGGGGAGGTGTTGGAGGTGGTGGAGGAATTGGAGGTGGTTTTATCGCTTCCAACTGCTGGCTGGATACCCGTCCGGCACCGGA<br>TGAACAGCTGGAAGCAAGCGCACAGAGCCGTCGTATGGCACAGGCACTGGATCAGCTGCCGGATCGTCAGCGTGAAGCAATTG<br>TTCTGCAGTATTATCAAGAACTGAGCAATACCGAAGCAGCAGCACTGATGCAAATTAGCGTTGAAGCCCTGGAAAGCCTGCTGA<br>GCCGTGCACGTCGTAATCTGCGTAGCCATCTGGCCGAAGCACCGGGTGCAGATCTGAGCGGTCGTCGCAAACCGTAAAGGTGA<br>TACTTTCAGCCAAAAAACTTAAGACCGCCGGTCTTGTCCACTACCTTGCAGTAATGCGGTGGACAGGATCGGCGGTTTTCTTTTC<br>TCTTCTCAAAGACCGTCCAATGGCGGCGCGCCATCGAATGGCGCAAAACCTTTCGCGGTATGGCATGATAGCGCCCGGAAGAG<br>AGTCAATTCAGGGTGGTGAATATGAAAAACATAAATGCCGACGACACATACAGAATAATTAATAAAATTAAAGCTTGTAGAAGCAA<br>TAATGATATTAATCAATGCTTATCTGATATGACTAAAATGGTACATTGTGAATATTATTTACTCGCGATCATTTATCCTCATTCTATG<br>GTTAAATCTGATATTTCAATCCTAGATAATTACCCTAAAAAATGGAGGCAATATTATGATGACGCTAATTTAATAAAATATGATCCTA<br>TAGTAGATTATTCTAACTCCAATCATTCACCAATTAATTGGAATATATTTGAAAACAATGCTGTAAATAAAAAATCTCCAAATGTAAT<br>TAAAGAAGCGAAAACATCAGGTCTTATCACTGGGTTTAGTTTCCCTATTCATACGGCTAACAATGGCTTCGGAATGCTTAGTTTTG<br>CACATTCAGAAAAAGACAACTATATAGATAGTTTATTTTTACATGCGTGTATGAACATACCATTAATTGTTCCTTCTCTAGTTGATAA<br>TTATCGAAAAATAAATATAGCAAATAATAAATCAAACAACGATTTAACCAAAAGAGAAAAAGAATGTTTAGCGTGGGCATGCGAAG<br>GAAAAAGCTCTTGGGATATTTCAAAAATATTAGGTTGCAGTGAGCGTACTGTCACTTTCCATTTAACCAATGCGCAAATGAAACTC<br>AATACAACAAACCGCTGCCAAAGTATTTCTAAAGCAATTTTAACAGGAGCAATTGATTGCCCATACTTTAAAAATTGATAAGGATC<br>CTAATTGGTAACGAATCAGACAATTGACGGCTCGAGGGAGTAGCATAGGGTTTGCAGAATCCCTGCTTCGTCCATTTGACAGGC<br>ACATTATGCATCGATGATAAGCTGTCAAACATGAGCAGATCCTCTACGCCGGACGCATCGTGGCCGGCATCACCGGCGCCACAG<br>GTGCGGTTGCTGGCGCCTATATCGCCGACATCACCGATGGGGAAGATCGGGCTCGCCACTTCGGGCTCATGAGCAAATATTTTA<br>TCTGGCTCACTCAAAGGCGGTAATGAC
pAMK-925	P_{Tac}_σ^{N}-Npu^{N} bait sensor	TCATGACCAAAATCCCTTAACGTGAGTTTTCGTTCCACTGAGCGTCAGACCCCGTAGAAAAGATCAAAGGATCTTCTTGAGATCC<br>TTTTTTTCTGCGCGTAATCTGCTGCTTGCAAACAAAAAAACCACCGCTACCAACGGTGGTTTGTTTGCCGGATCAAGAGCTACCA<br>ACTCTTTTTCCGAAGGTAACTGGCTTCAGCAGAGCGCAGATACCAAATACTGTCCTTCTAGTGTAGCCGTAGTTAGGCCACCACT<br>TCAAGAACTCTGTAGCACCGCCTACATACCTCGCTCTGCTAATCCTGTTACCAGTGGCTGCTGCCAGTGGCGATAAGTCGTGTCT<br>TACCGGGTTGGACTCAAGACGATAGTTACCGGATAAGGCGCAGCGGTCGGGCTGAACGGGGGGTTCGTGCACACAGCCCAGC<br>TTGGAGCGAACGACCTACACCGAACTGAGATACCTACAGCGTGAGCTATGAGAAAGCGCCACGCTTCCCGAAGGGAGAAAGGC<br>GGACAGGTATCCGGTAAGCGGCAGGGTCGGAACAGGAGAGCGCACGAGGGAGCTTCCAGGGGGAAACGCCTGGTATCTTTAT<br>AGTCCTGTCGGGTTTCGCCACCTCTGACTTGAGCGTCGATTTTTGTGATGCTCGTCAGGGGGGCGGAGCCTATGGAAAAACGCC<br>AGCAACGCGGCCTTTTTACGGTTCCTGGCCTTTTGCTGGCCTTTTGCTCACATGTTCTTTCCTGCGTTATCCCCTGATTCTGTGG<br>ATAACCGTCGATTATCAAAAAGGATCTTCACCTAGATCCTTTTAAATTAAAAATGAAGTTTTAAATCAATCTAAAGTATATATGAGTA<br>AACTTGGTCTGACAGTTATCAGAAGAACTCGTCAAGAAGGCGATAGAAGGCGATGCGCTGCGAATCGGGAGCGGCGATACCGT<br>AAAGCACGAGGAAGCGGTCAGCCCATTCGCCGCCAAGCTCTTCAGCAATATCACGGGTAGCCAACGCTATGTCCTGATAGCGG<br>TCCGCCACACCCAGCCGGCCACAGTCGATGAATCCAGAAAAGCGGCCATTTTCCACCATGATATTCGGCAAGCAGGCATCGCCA<br>TGGGTCACGACGAGATCCTCGCCGTCGGGCATGCGCGCCTTGAGCCTGGCGAACAGTTCGGCTGGCGCGAGCCCCTGATGCT<br>CTTCGTCCAGATCATCCTGATCGACAAGACCGGCTTCCATCCGAGTACGTGCTCGCTCGATGCGATGTTTCGCTTGGTGGTCGA<br>ATGGGCAGGTAGCCGGATCAAGCGTATGCAGCCGCCGCATTGCATCAGCCATGATGGATACTTTCTCGGCAGGAGCAAGGTGA<br>GATGACAGGAGATCCTGCCCCGGCACTTCGCCCAATAGCAGCCAGTCCCTTCCCGCTTCAGTGACAACGTCGAGCACAGCTGC<br>GCAAGGAACGCCCGTCGTGGCCAGCCACGATAGCCGCGCTGCCTCGTCCTGCAGTTCATTCAGGGCACCGGACAGGTCGGTC<br>TTGACAAAAAGAACCGGGCGCCCCTGCGCTGACAGCCGGAACACGGCGGCATCAGAGCAGCCGATTGTCTGTTGTGCCCAGTC<br>ATAGCCGAATAGCCTCTCCACCCAAGCGGCCGGAGAACCTGCGTGCAATCCATCTTGTTCAATCATGCGAAACGATCCTCAATAT<br>TATTGAAGCATTTATCAGGGTTATTGTCTCATGAGCGGATACATATTTGAATGTATTTAGAAAAATAAACAAATAGGGGTTCCGCG<br>CACATTTCCCCGAAAAGTGCCACCTGACGTCTAAGAAACCATTATTATCATGACATTAACCTATAAAAATAGGCGTATCACGAGGC<br>AGAATTTCAGATAAAAAAAATCCTTAGCTTTCGCTAAGGATGATTTCTGGAATTCGCGGCCGCTTCTAGAGGGAGCCAATTATTGA<br>AGGCCTCCCTAACGGGGGGCCTTTTTTTGTTTCTGGTCTCCCGCTTAACGATCGTTGGCTGTGTTGACAATTAATCATCGGCTCG<br>TATAATGTGTGGAATTGTGAGCGCTCACAATTAGCTGTCACCGGATGTGCTTTCCGGTCTGATGAGTCCGTGAGGACGAAACAG<br>CCTCTACAAATAATTTTGTTTAATACTTCATGGACGAAAGTACTAGATGAATGAAACCGATCCTGATCTGGAACTGCTGAAACGTA<br>TTGGTAATAATGATGCACAGGCCGTTAAAGAAATGGTTACCCGTAAACTGCCTCGTCTGCTGGCACTGGCAAGTCGCCTGCTGG<br>GTGATGCAGATGAAGCACGTGATATTGCACAAGAAAGTTTTCTGCGCATTTGGAAACAGGCAGCAAGCTGGCGTAGCGAACAGG<br>CACGTTTTGATACCTGGCTGCATCGTGTTGCACTGAATCTGTGTTATGATCGTCTGCGTCGTCGTAAAGAACATGTGCCGGTTGA<br>TAGCGAACATGCCTGTGAAGCATGCCTGAGCTACGAAACCGAAATCCTGACCGTTGAATATGGTCTGCTGCCGATCGGCAAAAT<br>CGTAGAAAAGCGTATCGAATGTACGGTTTACTCTGTCGATAACAACGGTAACATCTACACCCAGCCGGTAGCGCAGTGGCACGA<br>CCGTGGCGAACAAGAAGTGTTCGAGTACTGCCTGGAGGATGGCTCTCTGATCCGCGCTACTAAAGACCACAAATTTATGACCGT<br>GGACGGTCAAATGCTGCCGATCGATGAAATCTTTGAGCGCGAACTGGACCTGATGCGCGTGGACAACCTGCCGAACATCAAAAT<br>TGCTACCCGCAAGTATCTGGGTAAGCAGAACGTCTATGACATTGGTGTGGAGCGCGACCACAATTTCGCTCTGAAAAACTAAGC<br>TTAGAGCCAGGCATCAAATAAAACGAAAGGCTCAGTCGAAAGACTGGGCCTTTCGTTTTATCTGTTGTTTGTCGGTGAACGCTCT<br>CTACTAGAGTCACACTGGCTCACCTTCGGGTGGGCCTTTCTGCGTTTATACGCTTACTAGTAGCGGCCGCTGCAGTCCGGCAAA<br>AAAGGGCAAGGTGTCACCACCCTGCCCTTTTTCTTTAAAACCGAAAAGATTACTTCGCGTTATGCAGGCTTCCTCGCTCACTGAC<br>TCGCTGCGCTCGGTCGTTCGGCTGCGGCGAGCGGTATCAGCTCACTCAAAGGCGGTAAT
pTHSS-2132	P_{Tac}_σ^{N}-Npu^{N}-Mdm2 bait sensor	TCATGACCAAAATCCCTTAACGTGAGTTTTCGTTCCACTGAGCGTCAGACCCCGTAGAAAAGATCAAAGGATCTTCTTGAGATCC<br>TTTTTTTCTGCGCGTAATCTGCTGCTTGCAAACAAAAAAACCACCGCTACCAACGGTGGTTTGTTTGCCGGATCAAGAGCTACCA<br>ACTCTTTTTCCGAAGGTAACTGGCTTCAGCAGAGCGCAGATACCAAATACTGTCCTTCTAGTGTAGCCGTAGTTAGGCCACCACT<br>TCAAGAACTCTGTAGCACCGCCTACATACCTCGCTCTGCTAATCCTGTTACCAGTGGCTGCTGCCAGTGGCGATAAGTCGTGTCT<br>TACCGGGTTGGACTCAAGACGATAGTTACCGGATAAGGCGCAGCGGTCGGGCTGAACGGGGGGTTCGTGCACACAGCCCAGC<br>TTGGAGCGAACGACCTACACCGAACTGAGATACCTACAGCGTGAGCTATGAGAAAGCGCCACGCTTCCCGAAGGGAGAAAGGC<br>GGACAGGTATCCGGTAAGCGGCAGGGTCGGAACAGGAGAGCGCACGAGGGAGCTTCCAGGGGGAAACGCCTGGTATCTTTAT<br>AGTCCTGTCGGGTTTCGCCACCTCTGACTTGAGCGTCGATTTTTGTGATGCTCGTCAGGGGGGCGGAGCCTATGGAAAAACGCC<br>AGCAACGCGGCCTTTTTACGGTTCCTGGCCTTTTGCTGGCCTTTTGCTCACATGTTCTTTCCTGCGTTATCCCCTGATTCTGTGG<br>ATAACCGTCGATTATCAAAAAGGATCTTCACCTAGATCCTTTTAAATTAAAAATGAAGTTTTAAATCAATCTAAAGTATATATGAGTA<br>AACTTGGTCTGACAGTTATCAGAAGAACTCGTCAAGAAGGCGATAGAAGGCGATGCGCTGCGAATCGGGAGCGGCGATACCGT<br>AAAGCACGAGGAAGCGGTCAGCCCATTCGCCGCCAAGCTCTTCAGCAATATCACGGGTAGCCAACGCTATGTCCTGATAGCGG<br>TCCGCCACACCCAGCCGGCCACAGTCGATGAATCCAGAAAAGCGGCCATTTTCCACCATGATATTCGGCAAGCAGGCATCGCCA<br>TGGGTCACGACGAGATCCTCGCCGTCGGGCATGCGCGCCTTGAGCCTGGCGAACAGTTCGGCTGGCGCGAGCCCCTGATGCT<br>CTTCGTCCAGATCATCCTGATCGACAAGACCGGCTTCCATCCGAGTACGTGCTCGCTCGATGCGATGTTTCGCTTGGTGGTCGA<br>ATGGGCAGGTAGCCGGATCAAGCGTATGCAGCCGCCGCATTGCATCAGCCATGATGGATACTTTCTCGGCAGGAGCAAGGTGA<br>GATGACAGGAGATCCTGCCCCGGCACTTCGCCCAATAGCAGCCAGTCCCTTCCCGCTTCAGTGACAACGTCGAGCACAGCTGC<br>GCAAGGAACGCCCGTCGTGGCCAGCCACGATAGCCGCGCTGCCTCGTCCTGCAGTTCATTCAGGGCACCGGACAGGTCGGTC<br>TTGACAAAAAGAACCGGGCGCCCCTGCGCTGACAGCCGGAACACGGCGGCATCAGAGCAGCCGATTGTCTGTTGTGCCCAGTC<br>ATAGCCGAATAGCCTCTCCACCCAAGCGGCCGGAGAACCTGCGTGCAATCCATCTTGTTCAATCATGCGAAACGATCCTCAATAT<br>TATTGAAGCATTTATCAGGGTTATTGTCTCATGAGCGGATACATATTTGAATGTATTTAGAAAAATAAACAAATAGGGGTTCCGCG

		CACATTTCCCCGAAAAGTGCCACCTGACGTCTAAGAAACCATTATTATCATGACATTAACCTATAAAAATAGGCGTATCACGAGGC<br>AGAATTTCAGATAAAAAAAATCCTTAGCTTTCGCTAAGGATGATTTCTGGAATTCGCGGCCGCTTCTAGAGGGAGCCAATTATTGA<br>AGGCCTCCCTAACGGGGGGCCTTTTTTTGTTTCTGGTCTCCCGCTTAACGATCGTTGGCTGTGTTGACAATTAATCATCGGCTCG<br>TATAATGTGTGGAATTGTGAGCGCTCACAATTAGCTGTCACCGGATGTGCTTTCCGGTCTGATGAGTCCGTGAGGACGAAACAG<br>CCTCTACAAATAATTTTGTTTAATACTTCATGGACGAAAGTACTAGATGAATGAAACCGATCCTGATCTGGAACTGCTGAAACGTA<br>TTGGTAATAATGATGCACAGGCCGTTAAAGAAATGGTTACCCGTAAACTGCCTCGTCTGCTGGCACTGGCAAGTCGCCTGCTGG<br>GTGATGCAGATGAAGCACGTGATATTGCACAAGAAAGTTTTCTGCGCATTTGGAAACAGGCAGCAAGCTGGCGTAGCGAACAGG<br>CACGTTTTGATACCTGGCTGCATCGTGTTGCACTGAATCTGTGTTATGATCGTCTGCGTCGTCGTAAAGAACATGTGCCGGTTGA<br>TAGCGAACATGCCTGTGAAGCATGCCTGAGCTACGAAACCGAAATCCTGACCGTTGAATATGGTCTGCTGCCGATCGGCAAAAT<br>CGTAGAAAAGCGTATCGAATGTACGGTTTACTCTGTCGATAACAACGGTAACATCTACACCCAGCCGGTAGCGCAGTGGCACGA<br>CCGTGGCGAACAAGAAGTGTTCGAGTACTGCCTGGAGGATGGCTCTCTGATCCGCGCTACTAAAGACCACAAATTTATGACCGT<br>GGACGGTCAAATGCTGCCGATCGATGAAATCTTTGAGCGCGAACTGGACCTGATGCGCGTGGACAACCTGCCGAACATCAAAAT<br>TGCTACCCGCAAGTATCTGGGTAAGCAGAACGTCTATGACATTGGTGTGGAGCGCGACCACAATTTCGCTCTGAAAAACGGAGG<br>ATCTGGTGGAAGTGGTGGTTCTGGAGGTAGCCAGATCCCGGCGTCCGAACAAGAAACCCTGGTACGTCCGAAACCGCTGCTTC<br>TGAAACTGCTGAAGAGCGTGGGGGCGCAGAAAGATACCTACACCATGAAAGAAGTTCTGTTCTACCTGGGCCAGTATATCATGA<br>CCAAGCGCCTGTATGACGAAAAACAGCAACACATTGTTTACTGCTCTAACGACCTGCTGGGTGACCTGTTTGGTGTTCCTTCTTT<br>CTCCGTTAAAGAACACCGCAAAATCTATACCATGATCTACCGTAACCTGGTGGTAGTTAACCAGCAAGAATCTAGCGATTCCGGT<br>ACTAGCGTCAGCGAGAACTAAGCTTAGAGCCAGGCATCAAATAAAACGAAAGGCTCAGTCGAAAGACTGGGCCTTTCGTTTTATC<br>TGTTGTTTGTCGGTGAACGCTCTCTACTAGAGTCACACTGGCTCACCTTCGGGTGGGCCTTTCTGCGTTTATACGCTTACTAGTA<br>GCGGCCGCTGCAGTCCGGCAAAAAAGGGCAAGGTGTCACCACCCTGCCCTTTTTCTTTAAAACCGAAAAGATTACTTCGCGTTA<br>TGCAGGCTTCCTCGCTCACTGACTCGCTGCGCTCGGTCGTTCGGCTGCGGCGAGCGGTATCAGCTCACTCAAAGGCGGTAAT
pAMK-866	P_{J23105}_ σ^N-Npu^N-RBD bait sensor; Pσ_*cat-sfgfp* reporter	TCATGACCAAAATCCCTTAACGTGAGTTTTCGTTCCACTGAGCGTCAGACCCCGTAGAAAAGATCAAAGGATCTTCTTGAGATCC<br>TTTTTTTCTGCGCGTAATCTGCTGCTTGCAAACAAAAAAACCACCGCTACCAACGGTGGTTTGTTTGCCGGATCAAGAGCTACCA<br>ACTCTTTTTCCGAAGGTAACTGGCTTCAGCAGAGCGCAGATACCAAATACTGTCCTTCTAGTGTAGCCGTAGTTAGGCCACCACT<br>TCAAGAACTCTGTAGCACCGCCTACATACCTCGCTCTGCTAATCCTGTTACCAGTGGCTGCTGCCAGTGGCGATAAGTCGTGTCT<br>TACCGGGTTGGACTCAAGACGATAGTTACCGGATAAGGCGCAGCGGTCGGGCTGAACGGGGGGTTCGTGCACACAGCCCAGC<br>TTGGAGCGAACGACCTACACCGAACTGAGATACCTACAGCGTGAGCTATGAGAAAGCGCCACGCTTCCCGAAGGGAGAAAGGC<br>GGACAGGTATCCGGTAAGCGGCAGGGTCGGAACAGGAGAGCGCACGAGGGAGCTTCCAGGGGGAAACGCCTGGTATCTTTAT<br>AGTCCTGTCGGGTTTCGCCACCTCTGACTTGAGCGTCGATTTTTGTGATGCTCGTCAGGGGGGCGGAGCCTATGGAAAAACGCC<br>AGCAACGCGGCCTTTTTACGGTTCCTGGCCTTTTGCTGGCCTTTTGCTCACATGTTCTTTCCTGCGTTATCCCCTGATTCTGTGG<br>ATAACCGTCGATTATCAAAAAGGATCTTCACCTAGATCCTTTTAAATTAAAAATGAAGTTTTAAATCAATCTAAAGTATATATGAGTA<br>AACTTGGTCTGACAGTTATCAGAAGAACTCGTCAAGAAGGCGATAGAAGGCGATGCGCTGCGAATCGGGAGCGGCGATACCGT<br>AAAGCACGAGGAAGCGGTCAGCCCATTCGCCGCCAAGCTCTTCAGCAATATCACGGGTAGCCAACGCTATGTCCTGATAGCGG<br>TCCGCCACACCCAGCCGGCCACAGTCGATGAATCCAGAAAAGCGGCCATTTTCCACCATGATATTCGGCAAGCAGGCATCGCCA<br>TGGGTCACGACGAGATCCTCGCCGTCGGGCATGCGCGCCTTGAGCCTGGCGAACAGTTCGGCTGGCGCGAGCCCCTGATGCT<br>CTTCGTCCAGATCATCCTGATCGACAAGACCGGCTTCCATCCGAGTACGTGCTCGCTCGATGCGATGTTTCGCTTGGTGGTCGA<br>ATGGGCAGGTAGCCGGATCAAGCGTATGCAGCCGCCGCATTGCATCAGCCATGATGGATACTTTCTCGGCAGGAGCAAGGTGA<br>GATGACAGGAGATCCTGCCCCGGCACTTCGCCCAATAGCAGCCAGTCCCTTCCCGCTTCAGTGACAACGTCGAGCACAGCTGC<br>GCAAGGAACGCCCGTCGTGGCCAGCCACGATAGCCGCGCTGCCTCGTCCTGCAGTTCATTCAGGGCACCGGACAGGTCGGTC<br>TTGACAAAAAGAACCGGGCGCCCCTGCGCTGACAGCCGGAACACGGCGGCATCAGAGCAGCCGATTGTCTGTTGTGCCCAGTC<br>ATAGCCGAATAGCCTCTCCACCCAAGCGGCCGGAGAACCTGCGTGCAATCCATCTTGTTCAATCATGCGAAACGATCCTCAATAT<br>TATTGAAGCATTTATCAGGGTTATTGTCTCATGAGCGGATACATATTTGAATGTATTTAGAAAAATAAACAAATAGGGGTTCCGCG<br>CACATTTCCCCGAAAAGTGCCACCTGACGTCTAAGAAACCATTATTATCATGACATTAACCTATAAAAATAGGCGTATCACGAGGC<br>AGAATTTCAGATAAAAAAAATCCTTAGCTTTCGCTAAGGATGATTTCTGGAATTCGCGGCCGCTTCTAGAGGGAGGCGCGGATAA<br>AAATTTCATTTGCCCGCGACGGATTCCCCGCCCATCTATCGTTGAACCCATCAGCTGCGTTCATCAGCGAAGCTGTCACCGGAT<br>GTGCTTTCCGGTCTGATGAGTCCGTGAGGACGAAACAGCCTCTACAAATAATTTTGTTTAATACTTCACACAGGAAAGTACTAGAT<br>GAGCAAAGGAGAAGAACTTTTCACTGGAGTTGTCCCAATTCTTGTTGAATTAGATGGTGATGTTAATGGGCACAAATTTTCTGTCC<br>GTGGAGAGGGTGAAGGTGATGCTACAAACGGAAAACTCACCCTTAAATTTATTTGCACTACTGGAAAACTACCTGTTCCGTGGCC<br>AACACTTGTCACTACTCTGACCTATGGTGTTCAATGCTTTTCCCGTTATCCGGATCACATGAAACGGCATGACTTTTTCAAGAGTG<br>CCATGCCCGAAGGTTATGTACAGGAACGCACTATATCTTTCAAAGATGACGGGACCTACAAGACGCGTGCTGAAGTCAAGTTTG<br>AAGGTGATACCCTTGTTAATCGTATCGAGTTAAAGGGTATTGATTTTAAAGAAGATGGAAACATTCTTGGACACAAACTCGAGTAC<br>AACTTTAACTCACACAATGTATACATCACGGCAGACAAACAAAAGAATGGAATCAAAGCTAACTTCAAAATTCGCCACAACGTTGA<br>AGATGGTTCCGTTCAACTAGCAGACCATTATCAACAAAATACTCCAATTGGCGATGGCCCTGTCCTTTTACCAGACAACCATTACC<br>TGTCGACACAATCTGTCCTTTCGAAAGATCCCAACGAAAAGCGTGACCACATGGTCCTTCTTGAGTTTGTAACTGCTGCTGGGAT<br>TACACATGGCATGGATGAGCTCTACAAAGGAGGTGAGAAAAAAATCACTGGATATACCACCGTTGATATATCCCAATGGCATCGT<br>AAAGAACATTTTGAGGCATTTCAGTCAGTTGCTCAATGTACCTATAACCAGACCGTTCAGCTGGATATTACGGCCTTTTTAAAGAC<br>CGTAAAGAAAAATAAGCACAAGTTTTATCCGGCCTTTATTCACATTCTTGCCCGCCTGATGAATGCTCATCCGGAATTTCGTATGG<br>CAATGAAAGACGGTGAGCTGGTGATATGGGATAGTGTTCACCCTTGTTACACCGTTTTCCATGAGCAAACTGAAACGTTTTCATC<br>GCTCTGGAGTGAATACCACGACGATTTCCGGCAGTTTCTACACATATATTCGCAAGATGTGGCGTGTTACGGTGAAAACCTGGC<br>CTATTTCCCTAAAGGGTTTATTGAGAATATGTTTTTCGTCTCAGCCAATCCCTGGGTGAGTTTCACCAGTTTTGATTTAAACGTGG<br>CCAATATGGACAACTTCTTCGCCCCCGTTTTCACCATGGGCAAATATTATACGCAAGGCGACAAGGTGCTGATGCCGCTGGCGA<br>TTCAGGTTCATCATGCCGTTTGTGATGGCTTCCATGTCGGCAGAATGCTTAATGAATTACAACAGTACTGCGATGAGTGGCAGGG<br>CGGGGCGTAAAATGAAAGAGGGGAAATACTAGATGAAGATTGAAGAAGGCAAACTGGTCAGCGGCGAAAACCTGTATTTCCAGT<br>TCTTGTTCGTTCAAGAGTTGGTGGCGAGTGCCCATTCACATCTCGCAGAACTGGTTGCCAGTGCGAAGGCGGCCATTAGCCAGG<br>CGTCAGATGTTGCCGCGTTAGATAATGTGCGCGTCGAATATTTGGGTAAAAAAGGGCACTTAACCCTTCAGATGACGACCCTGC<br>GTGAGCTGCCGCCAGAAGAGCGTCCGGCAGCTGGTGCGGTTATCAACGAAGCGAAAGAGCAGGTTCAGCAGGCGCTGAATGC<br>GCGTAAAGCGGAACTGGAAAGCGCTGCACTGAATGCGCGTCTGGCGGCGGAAACGATTGATGTCTCTCTGCCAGGTCGTCGCA<br>TTGAAAACGGCGGTCTGCATCCGGTTACCCGTACCATCGACCGTATCGAAAGTTTCTTCGGTGAGCTTGGCTTTACCGTGGCAA<br>CCGGGCCGGAAATCGAGGATGATTATCATAACTTCGATGCTCTGAACATTCCTGGTCACCACCCGGCGCGCGCTGACCACGACA<br>CTTTCTGGTTTGACACCACCCGCCTGCTGCGTACCCAGACCTCTGGCGTACAGATCCGCACCATGAAAGCCCAGCAGCCACCG<br>ATTCGTATCATCGCGCCTGGCCGTGTTTATCGTAACGACTACGACCAGACTCACACGCCGATGTTCCATCAGATGGAAGGTCTG<br>ATTGTTGATACCAACATCAGCTTTACCAACCTGAAAGGCACGCTGCACGACTTCCTGCGTAACTTCTTTGAGGAAGATTTGCAGA<br>TTCGCTTCCGTCCTTCCTACTTCCCGTTTGCCGAACCTTCTGCAGAAGTGGACGTCATGGGTAAAAACGGTAAATGGCTGGAAGT<br>GCTGGGCTGCGGGATGGTGCATCCGAACGTGTTGCGTAACGTTGGCATCGACCCGGAAGTTTACTCTGGTTTCGGCTTCGGGA<br>TGGGGATGGAGCGTCTGACTATGTTGCGTTACGGCGTCACCGACCTGCGTTCATTCTTCGAAAACGATCTGCGTTTCCTCAAAC<br>AGTTTAAATAACATTACTCGCATCCATTCTCAGGCTCTCGGTACCAAATTCCAGAAAAGAGGCCTCCCGAAAGGGGGGCCTTTTT<br>TCGTTTTGGTCCTACTGGCGCGCCTTTACGGCTAGCTCAGTCCTAGGTACTATGCTAGCAAGGTAGACTGTCGCCGGATGTGTA<br>TCCGACCTGACGATGGCCCAAAAGGGCCGAAACAGTCCTCTACAAATAATTTTGTTTAATACTTCATGGACGAAAGTACTAGATG<br>AATGAAACCGATCCTGATCTGGAACTGCTGAAACGTATTGGTAATAATGATGCACAGGCCGTTAAAGAAATGGTTACCCGTAAAC<br>TGCCTCGTCTGCTGGCACTGGCAAGTCGCCTGCTGGGTGATGCAGATGAAGCACGTGATATTGCACAAGAAAGTTTTCTGCGCA<br>TTTGGAAACAGGCAGCAAGCTGGCGTAGCGAACAGGCACGTTTTGATACCTGGCTGCATCGTGTTGCACTGAATCTGTGTTATG<br>ATCGTCTGCGTCGTCGTAAAGAACATGTGCCGGTTGATAGCGAACATGCCTGTGAAGCATGCCTGAGCTACGAAACCGAAATCC<br>TGACCGTTGAATATGGTCTGCTGCCGATCGGCAAAATCGTAGAAAAGCGTATCGAATGTACGGTTTACTCTGTCGATAACAACGG<br>TAACATCTACACCCAGCCGGTAGCGCAGTGGCACGACCGTGGCGAACAAGAAGTGTTCGAGTACTGCCTGGAGGATGGCTCTC<br>TGATCCGCGCTACTAAAGACCACAAATTTATGACCGTGGACGGTCAAATGCTGCCGATCGATGAAATCTTTGAGCGCGAACTGG<br>ACCTGATGCGCGTGGACAACCTGCCGAACATCAAAATTGCTACCCGCAAGTATCTGGGTAAGCAGAACGTCTATGACATTGGTG<br>TGGAGCGCGACCACAATTTCGCTCTGAAAAACGGAGGATCTGGTGGAAGTGGTGGTTCTGGAGGTCGTTTTCCGAATATTACCA<br>ACTTATGCCCGTTTGGTGAGGTGTTCAACGCGACCCGCTTTGCCAGCGTATACGCGTGGAATCGTAAACGTATCTCGAACTGCG<br>TAGCGGATTACTCCGTGCTTTACAACTCAGCTTCCTTCTCCACCTTTAAATGTTATGGTGTTTCACCGACCAAGTTAAACGATCTG<br>TGCTTTACGAACGTCTATGCCGATTCATTTGTGATCAGAGGTGATGAGGTTCGTCAAATTGCGCCTGGACAGACAGGCAAAATTG<br>CAGACTATAACTACAAACTTCCCGACGATTTTACGGGCTGTGTTATTGCGTGGAATTCGAACAACCTGGATAGTAAGGTTGGAGG<br>GAATTATAACTATCTGTACCGCCTGTTTCGTAAATCTAACCTGAAACCTTTCGAACGCGACATATCAACTGAAATCTATCAGGCAG

		GTAGCACTCCCTGTAACGGTGTCGAGGGATTTAACTGCTATTTTCCTCTGCAGAGTTATGGCTTTCAGCCTACGAATGGAGTAGG<br>CTATCAACCGTACCGGGTGGTGGTTCTTAGTTTCGAGCTGCTGCATGCACCAGCCACAGTATGTGGCCCCAAAAAGTCAACGAA<br>TCTTTAACTCGGTACCAAATTCCAGAAAAGAGACGCTTTCGAGCGTCTTTTTTCGTTTTGGTCCCGCTTACTAGTAGCGGCCGCT<br>GCAGTCCGGCAAAAAAGGGCAAGGTGTCACCACCCTGCCCTTTTTCTTTAAAACCGAAAAGATTACTTCGCGTTATGCAGGCTTC<br>CTCGCTCACTGACTCGCTGCGCTCGGTCGTTCGGCTGCGGCGAGCGGTATCAGCTCACTCAAAGGCGGTAAT
pAMK-870	P_{J23105}_ σ^{N}-Npu^{N}-$Mdm2_{17\text{-}124}$ bait sensor; Pσ_*cat-sfgfp* reporter	TCATGACCAAAATCCCTTAACGTGAGTTTTCGTTCCACTGAGCGTCAGACCCCGTAGAAAAGATCAAAGGATCTTCTTGAGATCC<br>TTTTTTTCTGCGCGTAATCTGCTGCTTGCAAACAAAAAAACCACCGCTACCAACGGTGGTTTGTTTGCCGGATCAAGAGCTACCA<br>ACTCTTTTTCCGAAGGTAACTGGCTTCAGCAGAGCGCAGATACCAAATACTGTCCTTCTAGTGTAGCCGTAGTTAGGCCACCACT<br>TCAAGAACTCTGTAGCACCGCCTACATACCTCGCTCTGCTAATCCTGTTACCAGTGGCTGCTGCCAGTGGCGATAAGTCGTGTCT<br>TACCGGGTTGGACTCAAGACGATAGTTACCGGATAAGGCGCAGCGGTCGGGCTGAACGGGGGGTTCGTGCACACAGCCCAGC<br>TTGGAGCGAACGACCTACACCGAACTGAGATACCTACAGCGTGAGCTATGAGAAAGCGCCACGCTTCCCGAAGGGAGAAAGGC<br>GGACAGGTATCCGGTAAGCGGCAGGGTCGGAACAGGAGAGCGCACGAGGGAGCTTCCAGGGGGAAACGCCTGGTATCTTTAT<br>AGTCCTGTCGGGTTTCGCCACCTCTGACTTGAGCGTCGATTTTTGTGATGCTCGTCAGGGGGGCGGAGCCTATGGAAAAACGCC<br>AGCAACGCGGCCTTTTTACGGTTCCTGGCCTTTTGCTGGCCTTTTGCTCACATGTTCTTTCCTGCGTTATCCCCTGATTCTGTGG<br>ATAACCGTCGATTATCAAAAAGGATCTTCACCTAGATCCTTTTAAATTAAAAATGAAGTTTTAAATCAATCTAAAGTATATATGAGTA<br>AACTTGGTCTGACAGTTATCAGAAGAACTCGTCAAGAAGGCGATAGAAGGCGATGCGCTGCGAATCGGGAGCGGCGATACCGT<br>AAAGCACGAGGAAGCGGTCAGCCCATTCGCCGCCAAGCTCTTCAGCAATATCACGGGTAGCCAACGCTATGTCCTGATAGCGG<br>TCCGCCACACCCAGCCGGCCACAGTCGATGAATCCAGAAAAGCGGCCATTTTCCACCATGATATTCGGCAAGCAGGCATCGCCA<br>TGGGTCACGACGAGATCCTCGCCGTCGGGCATGCGCGCCTTGAGCCTGGCGAACAGTTCGGCTGGCGCGAGCCCCTGATGCT<br>CTTCGTCCAGATCATCCTGATCGACAAGACCGGCTTCCATCCGAGTACGTGCTCGCTCGATGCGATGTTTCGCTTGGTGGTCGA<br>ATGGGCAGGTAGCCGGATCAAGCGTATGCAGCCGCCGCATTGCATCAGCCATGATGGATACTTTCTCGGCAGGAGCAAGGTGA<br>GATGACAGGAGATCCTGCCCCGGCACTTCGCCCAATAGCAGCCAGTCCCTTCCCGCTTCAGTGACAACGTCGAGCACAGCTGC<br>GCAAGGAACGCCCGTCGTGGCCAGCCACGATAGCCGCGCTGCCTCGTCCTGCAGTTCATTCAGGGCACCGGACAGGTCGGTC<br>TTGACAAAAAGAACCGGGCGCCCCTGCGCTGACAGCCGGAACACGGCGGCATCAGAGCAGCCGATTGTCTGTTGTGCCCAGTC<br>ATAGCCGAATAGCCTCTCCACCCAAGCGGCCGGAGAACCTGCGTGCAATCCATCTTGTTCAATCATGCGAAACGATCCTCAATAT<br>TATTGAAGCATTTATCAGGGTTATTGTCTCATGAGCGGATACATATTTGAATGTATTTAGAAAAATAAACAAATAGGGGTTCCGCG<br>CACATTTCCCCGAAAAGTGCCACCTGACGTCTAAGAAACCATTATTATCATGACATTAACCTATAAAAATAGGCGTATCACGAGGC<br>AGAATTTCAGATAAAAAAAATCCTTAGCTTTCGCTAAGGATGATTTCTGGAATTCGCGGCCGCTTCTAGAGGGAGGCGCGGATAA<br>AAATTTCATTTGCCCGCGACGGATTCCCCGCCCATCTATCGTTGAACCCATCAGCTGCGTTCATCAGCGAAGCTGTCACCGGAT<br>GTGCTTTCCGGTCTGATGAGTCCGTGAGGACGAAACAGCCTCTACAAATAATTTTGTTTAATACTTCACACAGGAAAGTACTAGAT<br>GAGCAAAGGAGAAGAACTTTTCACTGGAGTTGTCCCAATTCTTGTTGAATTAGATGGTGATGTTAATGGGCACAAATTTTCTGTCC<br>GTGGAGAGGGTGAAGGTGATGCTACAAACGGAAAACTCACCCTTAAATTTATTTGCACTACTGGAAAACTACCTGTTCCGTGGCC<br>AACACTTGTCACTACTCTGACCTATGGTGTTCAATGCTTTTCCCGTTATCCGGATCACATGAAACGGCATGACTTTTTCAAGAGTG<br>CCATGCCCGAAGGTTATGTACAGGAACGCACTATATCTTTCAAAGATGACGGGACCTACAAGACGCGTGCTGAAGTCAAGTTTG<br>AAGGTGATACCCTTGTTAATCGTATCGAGTTAAAGGGTATTGATTTTAAAGAAGATGGAAACATTCTTGGACACAAACTCGAGTAC<br>AACTTTAACTCACACAATGTATACATCACGGCAGACAAACAAAAGAATGGAATCAAAGCTAACTTCAAAATTCGCCACAACGTTGA<br>AGATGGTTCCGTTCAACTAGCAGACCATTATCAACAAAATACTCCAATTGGCGATGGCCCTGTCCTTTTACCAGACAACCATTACC<br>TGTCGACACAATCTGTCCTTTCGAAAGATCCCAACGAAAAGCGTGACCACATGGTCCTTCTTGAGTTTGTAACTGCTGCTGGGAT<br>TACACATGGCATGGATGAGCTCTACAAAGGAGGTGAGAAAAAAATCACTGGATATACCACCGTTGATATATCCCAATGGCATCGT<br>AAAGAACATTTTGAGGCATTTCAGTCAGTTGCTCAATGTACCTATAACCAGACCGTTCAGCTGGATATTACGGCCTTTTTAAAGAC<br>CGTAAAGAAAAATAAGCACAAGTTTTATCCGGCCTTTATTCACATTCTTGCCCGCCTGATGAATGCTCATCCGGAATTTCGTATGG<br>CAATGAAAGACGGTGAGCTGGTGATATGGGATAGTGTTCACCCTTGTTACACCGTTTTCCATGAGCAAACTGAAACGTTTTCATC<br>GCTCTGGAGTGAATACCACGACGATTTCCGGCAGTTTCTACACATATATTCGCAAGATGTGGCGTGTTACGGTGAAAACCTGGC<br>CTATTTCCCTAAAGGGTTTATTGAGAATATGTTTTTCGTCTCAGCCAATCCCTGGGTGAGTTTCACCAGTTTTGATTTAAACGTGG<br>CCAATATGGACAACTTCTTCGCCCCCGTTTTCACCATGGGCAAATATTATACGCAAGGCGACAAGGTGCTGATGCCGCTGGCGA<br>TTCAGGTTCATCATGCCGTTTGTGATGGCTTCCATGTCGGCAGAATGCTTAATGAATTACAACAGTACTGCGATGAGTGGCAGGG<br>CGGGGCGTAAAATGAAAGAGGGGAAATACTAGATGAAGATTGAAGAAGGCAAACTGGTCAGCGGCGAAAACCTGTATTTCCAGT<br>TCTTGTTCGTTCAAGAGTTGGTGGCGAGTGCCCATTCACATCTCGCAGAACTGGTTGCCAGTGCGAAGGCGGCCATTAGCCAGG<br>CGTCAGATGTTGCCGCGTTAGATAATGTGCGCGTCGAATATTTGGGTAAAAAAGGGCACTTAACCCTTCAGATGACGACCCTGC<br>GTGAGCTGCCGCCAGAAGAGCGTCCGGCAGCTGGTGCGGTTATCAACGAAGCGAAAGAGCAGGTTCAGCAGGCGCTGAATGC<br>GCGTAAAGCGGAACTGGAAAGCGCTGCACTGAATGCGCGTCTGGCGGCGGAAACGATTGATGTCTCTCTGCCAGGTCGTCGCA<br>TTGAAAACGGCGGTCTGCATCCGGTTACCCGTACCATCGACCGTATCGAAAGTTTCTTCGGTGAGCTTGGCTTTACCGTGGCAA<br>CCGGGCCGGAAATCGAGGATGATTATCATAACTTCGATGCTCTGAACATTCCTGGTCACCACCCGGCGCGCGCTGACCACGACA<br>CTTTCTGGTTTGACACCACCCGCCTGCTGCGTACCCAGACCTCTGGCGTACAGATCCGCACCATGAAAGCCCAGCAGCCACCG<br>ATTCGTATCATCGCGCCTGGCCGTGTTTATCGTAACGACTACGACCAGACTCACACGCCGATGTTCCATCAGATGGAAGGTCTG<br>ATTGTTGATACCAACATCAGCTTTACCAACCTGAAAGGCACGCTGCACGACTTCCTGCGTAACTTCTTTGAGGAAGATTTGCAGA<br>TTCGCTTCCGTCCTTCCTACTTCCCGTTTGCCGAACCTTCTGCAGAAGTGGACGTCATGGGTAAAAACGGTAAATGGCTGGAAGT<br>GCTGGGCTGCGGGATGGTGCATCCGAACGTGTTGCGTAACGTTGGCATCGACCCGGAAGTTTACTCTGGTTTCGGCTTCGGGA<br>TGGGGATGGAGCGTCTGACTATGTTGCGTTACGGCGTCACCGACCTGCGTTCATTCTTCGAAAACGATCTGCGTTTCCTCAAAC<br>AGTTTAAATAACATTACTCGCATCCATTCTCAGGCTCTCGGTACCAAATTCCAGAAAAGAGGCCTCCCGAAAGGGGGGCCTTTTT<br>TCGTTTTGGTCCTACTGGCGCGCCTTTACGGCTAGCTCAGTCCTAGGTACTATGCTAGCAAGGTAGACTGTCGCCGGATGTGTA<br>TCCGACCTGACGATGGCCCAAAAGGGCCGAAACAGTCCTCTACAAATAATTTTGTTTAATACTTCATGGACGAAAGTACTAGATG<br>AATGAAACCGATCCTGATCTGGAACTGCTGAAACGTATTGGTAATAATGATGCACAGGCCGTTAAAGAAATGGTTACCCGTAAAC<br>TGCCTCGTCTGCTGGCACTGGCAAGTCGCCTGCTGGGTGATGCAGATGAAGCACGTGATATTGCACAAGAAAGTTTTCTGCGCA<br>TTTGGAAACAGGCAGCAAGCTGGCGTAGCGAACAGGCACGTTTTGATACCTGGCTGCATCGTGTTGCACTGAATCTGTGTTATG<br>ATCGTCTGCGTCGTCGTAAAGAACATGTGCCGGTTGATAGCGAACATGCCTGTGAAGCATGCCTGAGCTACGAAACCGAAATCC<br>TGACCGTTGAATATGGTCTGCTGCCGATCGGCAAAATCGTAGAAAAGCGTATCGAATGTACGGTTTACTCTGTCGATAACAACGG<br>TAACATCTACACCCAGCCGGTAGCGCAGTGGCACGACCGTGGCGAACAAGAAGTGTTCGAGTACTGCCTGGAGGATGGCTCTC<br>TGATCCGCGCTACTAAAGACCACAAATTTATGACCGTGGACGGTCAAATGCTGCCGATCGATGAAATCTTTGAGCGCGAACTGG<br>ACCTGATGCGCGTGGACAACCTGCCGAACATCAAAATTGCTACCCGCAAGTATCTGGGTAAGCAGAACGTCTATGACATTGGTG<br>TGGAGCGCGACCACAATTTCGCTCTGAAAAACGGAGGATCTGGTGGAAGTGGTGGTTCTGGAGGTAGCCAGATCCCGGCGTCC<br>GAACAAGAAACCCTGGTACGTCCGAAACCGCTGCTTCTGAAACTGCTGAAGAGCGTGGGGGCGCAGAAAGATACCTACACCAT<br>GAAAGAAGTTCTGTTCTACCTGGGCCAGTATATCATGACCAAGCGCCTGTATGACGAAAAACAGCAACACATTGTTTACTGCTCT<br>AACGACCTGCTGGGTGACCTGTTTGGTGTTCCTTCTTTCTCCGTTAAAGAACACCGCAAAATCTATACCATGATCTACCGTAACCT<br>GGTGGTAGTTAACCAGCAAGAATCTAGCGATTCCGGTACTAGCGTCAGCGAGAACTAACTCGGTACCAAATTCCAGAAAAGAGA<br>CGCTTTCGAGCGTCTTTTTTCGTTTTGGTCCCGCTTACTAGTAGCGGCCGCTGCAGTCCGGCAAAAAAGGGCAAGGTGTCACCA<br>CCCTGCCCTTTTTCTTTAAAACCGAAAAGATTACTTCGCGTTATGCAGGCTTCCTCGCTCACTGACTCGCTGCGCTCGGTCGTTC<br>GGCTGCGGCGAGCGGTATCAGCTCACTCAAAGGCGGTAAT
pEG-06_044	papB modifying expression plasmid	TTAATAAGATGATCTTCTTGAGATCGTTTTGGTCTGCGCGTAATCTCTTGCTCTGAAAACGAAAAAACCGCCTTGCAGGGCGGTTT<br>TTCGAAGGTTCTCTGAGCTACCAACTCTTTGAACCGAGGTAACTGGCTTGGAGGAGCGCAGTCACCAAAACTTGTCCTTTCAGTT<br>TAGCCTTAACCGGCGCATGACTTCAAGACTAACTCCTCTAAATCAATTACCAGTGGCTGCTGCCAGTGGTGCTTTTGCATGTCTT<br>TCCGGGTTGGACTCAAGACGATAGTTACCGGATAAGGCGCAGCGGTCGGACTGAACGGGGGGTTCGTGCATACAGTCCAGCTT<br>GGAGCGAACTGCCTACCCGGAACTGAGTGTCAGGCGTGGAATGAGACAAACGCGGCCATAACAGCGGAATGACACCGGTAAAC<br>CGAAAGGCAGGAACAGGAGAGCGCACGAGGGAGCCGCCAGGGGGAAACGCCTGGTATCTTTATAGTCCTGTCGGGTTTCGCC<br>ACCACTGATTTGAGCGTCAGATTTCGTGATGCTTGTCAGGGGGGCGGAGCCTATGGAAAAACGGCTTTGCCGCGGCCCTCTCA<br>CTTCCCTGTTAAGTATCTTCCTGGCATCTTCCAGGAAATCTCCGCCCCGTTCGTAAGCCATTTCCGCTCGCCGCAGTCGAACGAC<br>CGAGCGTAGCGAGTCAGTGAGCGAGGAAGCGGAATATATCCTGTATCACATATTCTGCTGACGCACCGGTGCAGCCTTTTTTCT<br>CCTGCCACATGAAGCACTTCACTGACACCCTCATCAGTGCCAACATAGTAAGCCAGTATACACTCCGCTACGATTATCAAAAAGG<br>ATCTTCACCTAGATCCTTTTAAATTAAAAATGAAGTTTTAAATCAATCTAAAGTATATATGAGTAAACTTGGTCTGATTATTTGCCGA<br>CTACCTTGGTGATCTCGCCTTTCACGTAGTGGACAAATTCTTCCAACTGATCTGTGCGCGAGGCCAAGCGATCTTCTTCTTGTCC<br>AAGATAAGCCTGTCTAGCTTCAAGTATGACGGGCTGATACTGGGCCGGCAGGCGCTCCATTGCCCAGTCGGCAGCGACATCCT

		TCGGCGCGATTTTGCCGGTTACTGCGCTGTACCAAATGCGGGACAACGTAAGCACTACATTTCGCTCATCGCCAGCCCAGTCGG<br>GCGGCGAGTTCCATAGCGTTAAGGTTTCATTTAGCGCCTCAAATAGATCCTGTTCAGGAACCGGATCAAAGAGTTCCTCCGCCG<br>CTGGACCTACCAAGGCAACGCTATGTTCTCTTGCTTTTGTCAGCAAGATAGCCAGATCAATGTCGATCGTGGCTGGCTCGAAGAT<br>ACCTGCAAGAATGTCATTGCGCTGCCATTCTCCAAATTGCAGTTCGCGCTTAGCTGGATAACGCCACGGAATGATGTCGTCGTG<br>CACAACAATGGTGACTTCTACAGCGCGGAGAATCTCGCTCTCTCCAGGGGAAGCCGAAGTTTCCAAAAGGTCGTTGATCAAAGC<br>TCGCCGCGTTGTTTCATCAAGCCTTACGGTCACCGTAACCAGCAAATCAATATCACTGTGTGGCTTCAGGCCGCCATCCACTGC<br>GGAGCCGTACAAATGTACGGCCAGCAACGTCGGTTCGAGATGGCGCTCGATGACGCCAACTACCTCTGATAGTTGAGTCGATAC<br>TTCGGCGATCACCGCTTCCCTCATTTTAGCTTCCTTAGCTCCTGAAAATCTCGATAACTCAAAAAATACGCCCGGTAGTGATCTTA<br>TTTCATTATGGTGAAAGTTGGAACCTCTTACGTGCCATTTCCCCGAAAAGTGCCACCTGACGTCTAAGAAACCATTATTATCATGA<br>CATTAACCTATAAAAATAGGCGTATCACGAGGCAGAATTTCAGATAAAAAAAATCCTTAGCTTTCGCTAAGGATGATTTCTGGAAT<br>TCGCGGCCGCTTCTAGAGCGCGAAGACTTACGAAAATCCTACGGATGCGGGCAAGCAAGCGCTTAACGATCGTTGGCTGAACA<br>AACAGACAATCTGGTCTGTTTGTATTATGGAAAATTTTTCTGTATAATAGATTCAACAAACAGACAATCTGGTCTGTTTGTATTATC<br>AGCGGTCAACGCATGTGCTTTGCGTTCTGATGAGACAGTGATGTCGAAACCGCCTCTACAAATAATTTTGTTTAAGCTCTTCAAG<br>AACTAAGGAGGTTAGAGGATGGCAAACCTGATCCAGGACCGCGAGGACGAACTGATTCATTTCCATCCGTACAAACTGTTCGAG<br>GTGGATTCAAAAACCTTCTTCTATAACGTAGTCACCAACGCGATTTTTGAAATTGATAGCCTGATAATCGACATTCTTCACTCAAAA<br>GGTAAAAATGAGGAGCACGTTGTGAAAGATTTGGCTGAACGCTATGAGCTGTCTCAGGTTCGCGAAGCGATCCAGAACATGAAA<br>GAGGCATACATTATAGCAACCGATGCTAACATCTCCGACGTAGAGAAGATGGGTATCTTAGATAACTCGCAGCGCGTTTTTAAAC<br>TGTCTAGCCTGACGCTCTTTATGGTGCAGGAATGCAACCTGCGGTGTACGTATTGTTACGGCGAAGAAGGAGAATACAACCAGA<br>AAGGTAAAATGACGTCCGAAATCGCCCGGAGCGCAGTGGATTTTCTGATTCAACAGAGTGGTGAAATCGAACAGTTGAACATCA<br>CATTCTTTGGAGGCGAACCGCTGCTCAACTTTCCATTAATACAAGAAACCGTGCAGTATGTGCACGAACAGAGCGAGATCCATAA<br>CAAGAAATTTAGCTTTTCCATCACCACCAATGGCACGCTCATTACCCCCAAAATCAAAAACTTCTTCTATAAACACCACTTTGCAG<br>TCCAGACTTCTATCGATGGTGATGAAAAGACGCACAATTTCAATCGCTTCTTCAAAGGAGGCCAGGGCTCTTATGATCTGCTGTT<br>AAAGCGGACGGAAGAAATGCGCAATGACCGTAAAATTGGTGCACGTGGAACCGTGACCCCTGCCGAGCTGGACCTCTCAAAAT<br>CATTTGACCACTTAGTTAAACTCGGCTTTCGCAAAATCTACTTATCACCCGCTTTATATAGTCTCTCTGACGATCACTACGACACC<br>CTGAGCAAAGAGATGGTCAAACTTGTTGAACAATTCCGTGAGCTGCTGGAGCGTGAAGATTACGTCACCGCGAAGAAAATGTCT<br>AATGTTCTGGGTATGTTATCGAAGATTCACTCCGGTGGCCCGCGCATTCATTTTTGCGGTGCCGGCACTAATGCTGCCGCTGTC<br>GATGTCCGCGGCAACCTTTTCCCGTGTCATCGTTTCGTGGGTGAAGATGAATGTTCAATCGGTAACCTGTTCGACGAGGACCCG<br>CTGTCAAAACAGTACAACTTTATAGAGAATTCTACAGTACGCAACCGTACTACGTGTTCGAAATGCTGGGCGAAGAATCTGTGCG<br>GCGGTGGTTGTCACCAAGAAAATTTCGCCGAGAATGGTAATGTGAACCAGCCAGTGGGCAAATTATGCAAAGTGACCAAAAACT<br>TCATCAACGCGACCATCAATCTGTACTTGCAACTTACTCAAGAACAACGCAGCATTCTGTTCGGCTAATAATGAAGAGCGGATGA<br>GCTCTACAAATAAGCAGAGGTGGTTGTGTTGCGAAAAAAAAAAAAAAACACCCTAACGGGTGTTTTTTTTTTTTTGGTGTCCCCAC<br>GTGTGGCGCTTTACAAAGTCTTCCTGTACTAGTAGCGGCCGCTGCAGTCCGGCAAAAAAGGGCAAGGTGTCACCACCCTGCCCT<br>TTTTCTTTAAAACCGAAAAGATTACTTCGCGTTATGCAGGCTTCCTCGCTCACTGACTCGCTGCGCTCGGTCGTTCGGCTGCGGC<br>GAGCGGTATCAGCTCACTCAAAGGCGGTAAT
pTHSS-2012	Cloning backbone	TTAATAAGATGATCTTCTTGAGATCGTTTTGGTCTGCGCGTAATCTCTTGCTCTGAAAACGAAAAAACCGCCTTGCAGGGCGGTTT<br>TTCGAAGGTTCTCTGAGCTACCAACTCTTTGAACCGAGGTAACTGGCTTGGAGGAGCGCAGTCACCAAAACTTGTCCTTTCAGTT<br>TAGCCTTAACCGGCGCATGACTTCAAGACTAACTCCTCTAAATCAATTACCAGTGGCTGCTGCCAGTGGTGCTTTTGCATGTCTT<br>TCCGGGTTGGACTCAAGACGATAGTTACCGGATAAGGCGCAGCGGTCGGACTGAACGGGGGGTTCGTGCATACAGTCCAGCTT<br>GGAGCGAACTGCCTACCCGGAACTGAGTGTCAGGCGTGGAATGAGACAAACGCGGCCATAACAGCGGAATGACACCGGTAAAC<br>CGAAAGGCAGGAACAGGAGAGCGCACGAGGGAGCCGCCAGGGGGAAACGCCTGGTATCTTTATAGTCCTGTCGGGTTTCGCC<br>ACCACTGATTTGAGCGTCAGATTTCGTGATGCTTGTCAGGGGGGCGGAGCCTATGGAAAAACGGCTTTGCCGCGGCCCTCTCA<br>CTTCCCTGTTAAGTATCTTCCTGGCATCTTCCAGGAAATCTCCGCCCCGTTCGTAAGCCATTTCCGCTCGCCGCAGTCGAACGAC<br>CGAGCGTAGCGAGTCAGTGAGCGAGGAAGCGGAATATATCCTGTATCACATATTCTGCTGACGCACCGGTGCAGCCTTTTTTCT<br>CCTGCCACATGAAGCACTTCACTGACACCCTCATCAGTGCCAACATAGTAAGCCAGTATACACTCCGCTACGATTATCAAAAAGG<br>ATCTTCACCTAGATCCTTTTAAATTAAAAATGAAGTTTTAAATCAATCTAAAGTATATATGAGTAAACTTGGTCTGACAGTTATTTGC<br>CGACTACCTTGGTGATCTCGCCTTTCACGTAGTGGACAAATTCTTCCAACTGATCTGTGCGCGAGGCCAAGCGATCTTCTTCTTG<br>TCCAAGATAAGCCTGTCTAGCTTCAAGTATGACGGGCTGATACTGGGCCGGCAGGCGCTCCATTGCCCAGTCGGCAGCGACAT<br>CCTTCGGCGCGATTTTGCCGGTTACTGCGCTGTACCAAATGCGGGACAACGTAAGCACTACATTTCGCTCATCGCCAGCCCAGT<br>CGGGCGGCGAGTTCCATAGCGTTAAGGTTTCATTTAGCGCCTCAAATAGATCCTGTTCAGGAACCGGATCAAAGAGTTCCTCCG<br>CCGCTGGACCTACCAAGGCAACGCTATGTTCTCTTGCTTTTGTCAGCAAGATAGCCAGATCAATGTCGATCGTGGCTGGCTCGA<br>AGATACCTGCAAGAATGTCATTGCGCTGCCATTCTCCAAATTGCAGTTCGCGCTTAGCTGGATAACGCCACGGAATGATGTCGTC<br>GTGCACAACAATGGTGACTTCTACAGCGCGGAGAATCTCGCTCTCTACAGGGGAAGCCGAAGTTTCCAAAAGGTCGTTGATCAA<br>AGCTCGCCGCGTTGTTTCATCAAGCCTTACGGTCACCGTAACCAGCAAATCAATATCACTGTGTGGCTTCAGGCCGCCATCCACT<br>GCGGAGCCGTACAAATGTACGGCCAGCAACGTCGGTTCGAGATGGCGCTCGATGACGCCAACTACCTCTGATAGTTGAGTCGA<br>TACTTCGGCGATCACCGCTTCCCTCATAACACCCCTTGTACAATATTATTGAAGCATTTATCAGGGTTATTGTCTCATGAGCGGAT<br>ACATATTTGAATGTATTTAGAAAAATAAACAAATAGGGGTTCCGCGCACATTTCCCCGAAAAGTGCCACCTGACGTCTAAGAAACC<br>ATTATTATCATGACATTAACCTATAAAAATAGGCGTATCACGAGGCAGAATTTCAGATAAAAAAAATCCTTAGCTTTCGCTAAGGAT<br>GATTTCTGGAATTCGCGGCCGCTTCTAGAGGGAGGTGTCTTCGGCGCGCCTTTACGGCTAGCTCAGTCCTAGGTACTATGCTAG<br>CAAGGTAGCTGTCACCGGATGTGCTTTCCGGTCTGATGAGTCCGTGAGGACGAAACAGCCTCTACAAATAATTTTGTTTAATCAC<br>ACAGGAAAGTACTAGATGAGCAAAGGAGAAGAACTTTTCACTGGAGTTGTCCCAATTCTTGTTGAATTAGATGGTGATGTTAATG<br>GGCACAAATTTTCTGTCCGTGGAGAGGGTGAAGGTGATGCTACAAACGGAAAACTCACCCTTAAATTTATTTGCACTACTGGAAA<br>ACTACCTGTTCCGTGGCCAACACTTGTCACTACTCTGACCTATGGTGTTCAATGCTTTTCCCGTTATCCGGATCACATGAAACGG<br>CATGACTTTTTCAAGAGTGCCATGCCCGAAGGTTATGTACAGGAACGCACTATATCTTTCAAAGATGACGGGACCTACAAGACGC<br>GTGCTGAAGTCAAGTTTGAAGGTGATACCCTTGTTAATCGTATCGAGTTAAAGGGTATTGATTTTAAAGAAGATGGAAACATTCTT<br>GGACACAAACTCGAGTACAACTTTAACTCACACAATGTATACATCACGGCAGACAAACAAAAGAATGGAATCAAAGCTAACTTCA<br>AAATTCGCCACAACGTTGAAGATGGTTCCGTTCAACTAGCAGACCATTATCAACAAAATACTCCAATTGGCGATGGCCCTGTCCT<br>TTTACCAGACAACCATTACCTGTCGACACAATCTGTCCTTTCGAAAGATCCCAACGAAAAGCGTGACCACATGGTCCTTCTTGAG<br>TTTGTAACTGCTGCTGGGATTACACATGGCATGGATGAGCTCTACAAATAATACTAGAGCCAGGCATCAAATAAAACGAAAGGCT<br>CAGTCGAAAGACTGGGCCTTTCGTTTTATCTGTTGTTTGTCGGTGAACGCTCTCTACTAGAGTCACACTGGCTCACCTTCGGGTG<br>GGCCTTTCTGCGTTTATAGAAGACATCGCTTACTAGTAGCGGCCGCTGCAGTCCGGCAAAAAAGGGCAAGGTGTCACCACCCTG<br>CCCTTTTTCTTTAAAACCGAAAAGATTACTTCGCGTTATGCAGGCTTCCTCGCTCACTGACTCGCTGCGCTCGGTCGTTCGGCTG<br>CGGCGAGCGGTATCAGCTCACTCAAAGGCGGTAAT
pAMK-926	P_{Lux}_Npu^{C}-σ^{C} peptide sensor	TTAATAAGATGATCTTCTTGAGATCGTTTTGGTCTGCGCGTAATCTCTTGCTCTGAAAACGAAAAAACCGCCTTGCAGGGCGGTTT<br>TTCGAAGGTTCTCTGAGCTACCAACTCTTTGAACCGAGGTAACTGGCTTGGAGGAGCGCAGTCACCAAAACTTGTCCTTTCAGTT<br>TAGCCTTAACCGGCGCATGACTTCAAGACTAACTCCTCTAAATCAATTACCAGTGGCTGCTGCCAGTGGTGCTTTTGCATGTCTT<br>TCCGGGTTGGACTCAAGACGATAGTTACCGGATAAGGCGCAGCGGTCGGACTGAACGGGGGGTTCGTGCATACAGTCCAGCTT<br>GGAGCGAACTGCCTACCCGGAACTGAGTGTCAGGCGTGGAATGAGACAAACGCGGCCATAACAGCGGAATGACACCGGTAAAC<br>CGAAAGGCAGGAACAGGAGAGCGCACGAGGGAGCCGCCAGGGGGAAACGCCTGGTATCTTTATAGTCCTGTCGGGTTTCGCC<br>ACCACTGATTTGAGCGTCAGATTTCGTGATGCTTGTCAGGGGGGCGGAGCCTATGGAAAAACGGCTTTGCCGCGGCCCTCTCA<br>CTTCCCTGTTAAGTATCTTCCTGGCATCTTCCAGGAAATCTCCGCCCCGTTCGTAAGCCATTTCCGCTCGCCGCAGTCGAACGAC<br>CGAGCGTAGCGAGTCAGTGAGCGAGGAAGCGGAATATATCCTGTATCACATATTCTGCTGACGCACCGGTGCAGCCTTTTTTCT<br>CCTGCCACATGAAGCACTTCACTGACACCCTCATCAGTGCCAACATAGTAAGCCAGTATACACTCCGCTACGATTATCAAAAAGG<br>ATCTTCACCTAGATCCTTTTAAATTAAAAATGAAGTTTTAAATCAATCTAAAGTATATATGAGTAAACTTGGTCTGACAGTTATTTGC<br>CGACTACCTTGGTGATCTCGCCTTTCACGTAGTGGACAAATTCTTCCAACTGATCTGTGCGCGAGGCCAAGCGATCTTCTTCTTG<br>TCCAAGATAAGCCTGTCTAGCTTCAAGTATGACGGGCTGATACTGGGCCGGCAGGCGCTCCATTGCCCAGTCGGCAGCGACAT<br>CCTTCGGCGCGATTTTGCCGGTTACTGCGCTGTACCAAATGCGGGACAACGTAAGCACTACATTTCGCTCATCGCCAGCCCAGT<br>CGGGCGGCGAGTTCCATAGCGTTAAGGTTTCATTTAGCGCCTCAAATAGATCCTGTTCAGGAACCGGATCAAAGAGTTCCTCCG<br>CCGCTGGACCTACCAAGGCAACGCTATGTTCTCTTGCTTTTGTCAGCAAGATAGCCAGATCAATGTCGATCGTGGCTGGCTCGA<br>AGATACCTGCAAGAATGTCATTGCGCTGCCATTCTCCAAATTGCAGTTCGCGCTTAGCTGGATAACGCCACGGAATGATGTCGTC<br>GTGCACAACAATGGTGACTTCTACAGCGCGGAGAATCTCGCTCTCTACAGGGGAAGCCGAAGTTTCCAAAAGGTCGTTGATCAA<br>AGCTCGCCGCGTTGTTTCATCAAGCCTTACGGTCACCGTAACCAGCAAATCAATATCACTGTGTGGCTTCAGGCCGCCATCCACT

		GCGGAGCCGTACAAATGTACGGCCAGCAACGTCGGTTCGAGATGGCGCTCGATGACGCCAACTACCTCTGATAGTTGAGTCGA<br>TACTTCGGCGATCACCGCTTCCCTCATAACACCCCTTGTACAATATTATTGAAGCATTTATCAGGGTTATTGTCTCATGAGCGGAT<br>ACATATTTGAATGTATTTAGAAAAATAAACAAATAGGGGTTCCGCGCACATTTCCCCGAAAAGTGCCACCTGACGTCTAAGAAACC<br>ATTATTATCATGACATTAACCTATAAAAATAGGCGTATCACGAGGCAGAATTTCAGATAAAAAAAATCCTTAGCTTTCGCTAAGGAT<br>GATTTCTGGAATTCGCGGCCGCTTCTAGAGGGAGCCAATTATTGAAGGCCTCCCTAACGGGGGGCCTTTTTTTGTTTCTGGTCTC<br>CCGCTTAACGATCGTTGGCTGACCTGTAGGATCGTACAGGTTTACGCAAGAAAATGGTTTGTTACAGTCGAATAAAAGCTGTCAC<br>CGGATGTGCTTTCCGGTCTGATGAGTCCGTGAGGACGAAACAGCCTCTACAAATAATTTTGTTTAAAGGTTCACACAGGAAAGTA<br>CTAGATGGGAGGTGTTGGAGGTGGTGGAGGAATTGGAGGTGGTTTTATCGCTTCCAACTGCTGGCTGGATACCCGTCCGGCAC<br>CGGATGAACAGCTGGAAGCAAGCGCACAGAGCCGTCGTATGGCACAGGCACTGGATCAGCTGCCGGATCGTCAGCGTGAAGC<br>AATTGTTCTGCAGTATTATCAAGAACTGAGCAATACCGAAGCAGCAGCACTGATGCAAATTAGCGTTGAAGCCCTGGAAAGCCTG<br>CTGAGCCGTGCACGTCGTAATCTGCGTAGCCATCTGGCCGAAGCACCGGGTGCAGATCTGAGCGGTCGTCGCAAACCGTAATA<br>GGTCCAGGCATCAAATAAAACGAAAGGCTCAGTCGAAAGACTGGGCCTTTCGTTTTATCTGTTGTTTGTCGGTGAACGCTCTCTA<br>CTAGAGTCACACTGGCTCACCTTCGGGTGGGCCTTTCTGCGTTTATACGCTTACTAGTAGCGGCCGCTGCAGTCCGGCAAAAAA<br>GGGCAAGGTGTCACCACCCTGCCCTTTTTCTTTAAAACCGAAAAGATTACTTCGCGTTATGCAGGCTTCCTCGCTCACTGACTCG<br>CTGCGCTCGGTCGTTCGGCTGCGGCGAGCGGTATCAGCTCACTCAAAGGCGGTAAT
pTHSS-2137	P_{Lux}_PMI-Npu^C-σ^C peptide sensor	TTAATAAGATGATCTTCTTGAGATCGTTTTGGTCTGCGCGTAATCTCTTGCTCTGAAAACGAAAAAACCGCCTTGCAGGGCGGTTT<br>TTCGAAGGTTCTCTGAGCTACCAACTCTTTGAACCGAGGTAACTGGCTTGGAGGAGCGCAGTCACCAAAACTTGTCCTTTCAGTT<br>TAGCCTTAACCGGCGCATGACTTCAAGACTAACTCCTCTAAATCAATTACCAGTGGCTGCTGCCAGTGGTGCTTTTGCATGTCTT<br>TCCGGGTTGGACTCAAGACGATAGTTACCGGATAAGGCGCAGCGGTCGGACTGAACGGGGGGTTCGTGCATACAGTCCAGCTT<br>GGAGCGAACTGCCTACCCGGAACTGAGTGTCAGGCGTGGAATGAGACAAACGCGGCCATAACAGCGGAATGACACCGGTAAAC<br>CGAAAGGCAGGAACAGGAGAGCGCACGAGGGAGCCGCCAGGGGGAAACGCCTGGTATCTTTATAGTCCTGTCGGGTTTCGCC<br>ACCACTGATTTGAGCGTCAGATTTCGTGATGCTTGTCAGGGGGGCGGAGCCTATGGAAAAACGGCTTTGCCGCGGCCCTCTCA<br>CTTCCCTGTTAAGTATCTTCCTGGCATCTTCCAGGAAATCTCCGCCCCGTTCGTAAGCCATTTCCGCTCGCCGCAGTCGAACGAC<br>CGAGCGTAGCGAGTCAGTGAGCGAGGAAGCGGAATATATCCTGTATCACATATTCTGCTGACGCACCGGTGCAGCCTTTTTTCT<br>CCTGCCACATGAAGCACTTCACTGACACCCTCATCAGTGCCAACATAGTAAGCCAGTATACACTCCGCTACGATTATCAAAAAGG<br>ATCTTCACCTAGATCCTTTTAAATTAAAAATGAAGTTTTAAATCAATCTAAAGTATATATGAGTAAACTTGGTCTGACAGTTATTTGC<br>CGACTACCTTGGTGATCTCGCCTTTCACGTAGTGGACAAATTCTTCCAACTGATCTGTGCGCGAGGCCAAGCGATCTTCTTCTTG<br>TCCAAGATAAGCCTGTCTAGCTTCAAGTATGACGGGCTGATACTGGGCCGGCAGGCGCTCCATTGCCCAGTCGGCAGCGACAT<br>CCTTCGGCGCGATTTTGCCGGTTACTGCGCTGTACCAAATGCGGGACAACGTAAGCACTACATTTCGCTCATCGCCAGCCCAGT<br>CGGGCGGCGAGTTCCATAGCGTTAAGGTTTCATTTAGCGCCTCAAATAGATCCTGTTCAGGAACCGGATCAAAGAGTTCCTCCG<br>CCGCTGGACCTACCAAGGCAACGCTATGTTCTCTTGCTTTTGTCAGCAAGATAGCCAGATCAATGTCGATCGTGGCTGGCTCGA<br>AGATACCTGCAAGAATGTCATTGCGCTGCCATTCTCCAAATTGCAGTTCGCGCTTAGCTGGATAACGCCACGGAATGATGTCGTC<br>GTGCACAACAATGGTGACTTCTACAGCGCGGAGAATCTCGCTCTCTACAGGGGAAGCCGAAGTTTCCAAAAGGTCGTTGATCAA<br>AGCTCGCCGCGTTGTTTCATCAAGCCTTACGGTCACCGTAACCAGCAAATCAATATCACTGTGTGGCTTCAGGCCGCCATCCACT<br>GCGGAGCCGTACAAATGTACGGCCAGCAACGTCGGTTCGAGATGGCGCTCGATGACGCCAACTACCTCTGATAGTTGAGTCGA<br>TACTTCGGCGATCACCGCTTCCCTCATAACACCCCTTGTACAATATTATTGAAGCATTTATCAGGGTTATTGTCTCATGAGCGGAT<br>ACATATTTGAATGTATTTAGAAAAATAAACAAATAGGGGTTCCGCGCACATTTCCCCGAAAAGTGCCACCTGACGTCTAAGAAACC<br>ATTATTATCATGACATTAACCTATAAAAATAGGCGTATCACGAGGCAGAATTTCAGATAAAAAAAATCCTTAGCTTTCGCTAAGGAT<br>GATTTCTGGAATTCGCGGCCGCTTCTAGAGGGAGCCAATTATTGAAGGCCTCCCTAACGGGGGGCCTTTTTTTGTTTCTGGTCTC<br>CCGCTTAACGATCGTTGGCTGACCTGTAGGATCGTACAGGTTTACGCAAGAAAATGGTTTGTTACAGTCGAATAAAAGCTGTCAC<br>CGGATGTGCTTTCCGGTCTGATGAGTCCGTGAGGACGAAACAGCCTCTACAAATAATTTTGTTTAATACTTCACACAGGAAAGTA<br>CTAGATGACCAGCTTTGCGGAATATTGGAACCTGCTGAGCCCGGGAGGTGTTGGAGGTGGTGGAGGAATTGGAGGTGGTTTTA<br>TCGCTTCCAACTGCTGGCTGGATACCCGTCCGGCACCGGATGAACAGCTGGAAGCAAGCGCACAGAGCCGTCGTATGGCACAG<br>GCACTGGATCAGCTGCCGGATCGTCAGCGTGAAGCAATTGTTCTGCAGTATTATCAAGAACTGAGCAATACCGAAGCAGCAGCA<br>CTGATGCAAATTAGCGTTGAAGCCCTGGAAAGCCTGCTGAGCCGTGCACGTCGTAATCTGCGTAGCCATCTGGCCGAAGCACC<br>GGGTGCAGATCTGAGCGGTCGTCGCAAACCGTAATAGGTCCAGGCATCAAATAAAACGAAAGGCTCAGTCGAAAGACTGGGCC<br>TTTCGTTTTATCTGTTGTTTGTCGGTGAACGCTCTCTACTAGAGTCACACTGGCTCACCTTCGGGTGGGCCTTTCTGCGTTTATAC<br>GCTTACTAGTAGCGGCCGCTGCAGTCCGGCAAAAAAGGGCAAGGTGTCACCACCCTGCCCTTTTTCTTTAAAACCGAAAAGATT<br>ACTTCGCGTTATGCAGGCTTCCTCGCTCACTGACTCGCTGCGCTCGGTCGTTCGGCTGCGGCGAGCGGTATCAGCTCACTCAAA<br>GGCGGTAAT
pAMK-670	P_{Lux}_PMI-Npu^C-σ^C peptide sensor	AGTAAGACGGGTAAGCCTGTTGATGATACCGCTGCCTTACTGGGTGCATTAGCCAGTCTGAATGACCTGTCACGGGATAATCCG<br>AAGTGGTCAGACTGGAAAATCAGAGGGCAGGAACTGCTGAACAGCAAAAAGTCAGATAGCACCACATAGCAGACCCGCCATAAA<br>ACGCCCTGAGAAGCCCGTGACGGGCTTTTCTTGTATTATGGGTAGTTTCCTTGCATGAATCCATAAAAGGCGCCTGTAGTGCCAT<br>TTACCCCCATTCACTGCCAGAGCCGTGAGCGCAGCGAACTGAATGTCACGAAAAAGACAGCGACTCAGGTGCCTGATGGTCGG<br>AGACAAAAGGAATATTCAGCGATTTGCCCGAGCTTGCGAGGGTGCTACTTAAGCCTTTAGGGTTTTAAGGTCTGTTTTGTAGAGG<br>AGCAAACAGCGTTTGCGACATCCTTTTGTAATACTGCGGAACTGACTAAAGTAGTGAGTTATACACAGGGCTGGGATCTATTCTT<br>TTTATCTTTTTTTATTCTTTCTTTATTCTATAAATTATAACCACTTGAATATAAACAAAAAAAACACACAAAGGTCTAGCGGAATTTAC<br>AGAGGGTCTAGCAGAATTTACAAGTTTTCCAGCAAAGGTCTAGCAGAATTTACAGATACCCACAACTCAAAGGAAAAGGACTAGT<br>AATTATCATTGACTAGCCCATCTCAATTGGTATAGTGATTAAAATCACCTAGACCAATTGAGATGTATGTCTGAATTAGTTGTTTTC<br>AAAGCAAATGAACTAGCGATTAGTCGCTATGACTTAACGGAGCATGAAACCAAGCTAATTTTATGCTGTGTGGCACTACTCAACC<br>CCACGATTGAAAACCCTACAAGGAAAGAACGGACGGTATCGTTCACTTATAACCAATACGCTCAGATGATGAACATCAGTAGGGA<br>AAATGCTTATGGTGTATTAGCTAAAGCAACCAGAGAGCTGATGACGAGAACTGTGGAAATCAGGAATCCTTTGGTTAAAGGCTTT<br>TGGATTTTCCAGTGGACAAACTATGCCAAGTTCTCAAGCGAAAAATTAGAATTAGTTTTTAGTGAAGAGATATTGCCTTATCTTTTC<br>CAGTTAAAAAAATTCATAAAATATAATCTGGAACATGTTAAGTCTTTTGAAAACAAATACTCTATGAGGATTTATGAGTGGTTATTAA<br>AAGAACTAACACAAAAGAAAACTCACAAGGCAAATATAGAGATTAGCCTTGATGAATTTAAGTTCATGTTAATGCTTGAAAATAACT<br>ACCATGAGTTTAAAAGGCTTAACCAATGGGTTTTGAAACCAATAAGTAAAGATTTAAACACTTACAGCAATATGAAATTGGTGGTT<br>GATAAGCGAGGCCGCCCGACTGATACGTTGATTTTCCAAGTTGAACTAGATAGACAAATGGATCTCGTAACCGAACTTGAGAACA<br>ACCAGATAAAAATGAATGGTGACAAAATACCAACAACCATTACATCAGATTCCTACCTACATAACGGACTAAGAAAAACACTACAC<br>GATGCTTTAACTGCAAAAATTCAGCTCACCAGTTTTGAGGCAAAATTTTTGAGTGACATGCAAAGTAAGTATGATCTCAATGGTTC<br>GTTCTCATGGCTCACGCAAAAACAACGAACCACACTAGAGAACATACTGGCTAAATACGGAAGGATCTGAGGTTCTTATGGCTCT<br>TGTATCTATCAGTGAAGCATCAAGACTAACAAACAAAAGTAGAACAACTGTTCACCGTTACATATCAAAGGGAAAACTGTCCATAT<br>GCACAGATGAAAACGGTGTAAAAAAGATAGATACATCAGAGCTTTTACGAGTTTTTGGTGCATTCAAAGCTGTTCACCATGAACA<br>GATCGACAATGTAACAGATGAACAGCATGTAACACCTAATAGAACAGGTGAAACCAGTAAAACAAAGCAACTAGAACATGAAATT<br>GAACACCTGAGACAACTTGTTACAGCTCAACAGTCACACATAGACAGCCTGAAACAGGCGATGCTGCTTATCGAATCAAAGCTGC<br>CGACAACACGGGAGCCAGTGACGCCTCCCGTGGGGAAAAAATCATGGCAATTCTGGAAGAAATAGCGCTTTCAGCCGGCAAAC<br>CGGCTGAAGCCGGATCTGCGATTCTGATAACAAACTAGCAACACCAGAACAGCCCGTTTGCGGGCAGCAAAACCCGTACCGATT<br>ATCAAAAAGGATCTTCACCTAGATCCTTTTAAATTAAAAATGAAGTTTTAAATCAATCTAAAGTATATATGAGTAAACTTGGTCTGAC<br>AGTTACCAATGCTTAATCAGTGAGGCACCTATCTCAGCGATCTGTCTATTTCGTTCATCCATAGTTGCCTGACTCCCCGTCGTGTA<br>GATAACTACGATACGGGAGGGCTTACCATCTGGCCCCAGTGCTGCAATGATACCGCGAGAACCACGCTCACCGGCTCCAGATTT<br>ATCAGCAATAAACCAGCCAGCCGGAAGGGCCGAGCGCAGAAGTGGTCCTGCAACTTTATCCGCCTCCATCCAGTCTATTAATTG<br>TTGCCGGGAAGCTAGAGTAAGTAGTTCGCCAGTTAATAGTTTGCGCAACGTTGTTGCCATTGCTACAGGCATCGTGGTGTCACG<br>CTCGTCGTTTGGTATGGCTTCATTCAGCTCCGGTTCCCAACGATCAAGGCGAGTTACATGATCCCCCATGTTGTGCAAAAAAGCG<br>GTTAGCTCCTTCGGTCCTCCGATCGTTGTCAGAAGTAAGTTGGCCGCAGTGTTATCACTCATGGTTATGGCAGCACTGCATAATT<br>CTCTTACTGTCATGCCATCCGTAAGATGCTTTTCTGTGACTGGTGAGTACTCAACCAAGTCATTCTGAGAATAGTGTATGCGGCG<br>ACCGAGTTGCTCTTGCCCGGCGTCAATACGGGATAATACCGCGCCACATAGCAGAACTTTAAAAGTGCTCATCATTGGAAAACGT<br>TCTTCGGGGCGAAAACTCTCAAGGATCTTACCGCTGTTGAGATCCAGTTCGATATAACCCACTCGTGCACCCAACTGATCTTCAG<br>CATCTTTTACTTTCACCAGCGTTTCTGGGTGAGCAAAAACAGGAAGGCAAAATGCCGCAAAAAAGGGAATAAGGGCGACACGGA<br>AATGTTGAATACTCATACTCTTCCTTTTTCAATATTATTGAAGCATTTATCAGGGTTATTGTCTCATGAGCGGATACATATTTGAATG<br>TATTTAGAAAAATAAACAAATAGGGGTTCCGCGCACATTTCCCCGAAAAGTGCCACCTGACGTCTAAGAAGAATTCGCGGCCGCT<br>TCTAGAGGGAGCCAATTATTGAAGGCCTCCCTAACGGGGGGCCTTTTTTTGTTTCTGGTCTCCCGCTTAACGATCGTTGGCTGAC<br>CTGTAGGATCGTACAGGTTTACGCAAGAAAATGGTTTGTTACAGTCGAATAAAAGCTGTCACCGGATGTGCTTTCCGGTCTGATG

		AGTCCGTGAGGACGAAACAGCCTCTACAAATAATTTTGTTTAATCCATCTCTATGGCGGATTTTATGTCATATTACCACCATCACC<br>ATCATCACATGAGCCAACTGGCGGAGCTTTCTGAGGAGGCTCTGGGAGGGGTCGATGCCTCGTACGCGGTGTTCTGGCCGATC<br>TGTGGCACCAGCTTTGCGGAATATTGGAACCTGCTGAGCCCGCGAGGAGGTAAGGGAGGACCTGGAGGTCGGGGAGGTGTTG<br>GAGGTGGTGGAGGAATTGGAGGTGGTTTTATCGCTTCCAACTGCTGGCTGGATACCCGTCCGGCACCGGATGAACAGCTGGAA<br>GCAAGCGCACAGAGCCGTCGTATGGCACAGGCACTGGATCAGCTGCCGGATCGTCAGCGTGAAGCAATTGTTCTGCAGTATTAT<br>CAAGAACTGAGCAATACCGAAGCAGCAGCACTGATGCAAATTAGCGTTGAAGCCCTGGAAAGCCTGCTGAGCCGTGCACGTCG<br>TAATCTGCGTAGCCATCTGGCCGAAGCACCGGGTGCAGATCTGAGCGGTCGTCGCAAACCGTAAAGGTGATACTTTCAGCCAAA<br>AAACTTAAGACCGCCGGTCTTGTCCACTACCTTGCAGTAATGCGGTGGACAGGATCGGCGGTTTTCTTTTCTCTTCTCAAAGACC<br>GTCCAATGGCGGCGCGCCATCGAATGGCGCAAAACCTTTCGCGGTATGGCATGATAGCGCCCGGAAGAGAGTCAATTCAGGGT<br>GGTGAATATGAAAAACATAAATGCCGACGACACATACAGAATAATTAATAAAATTAAAGCTTGTAGAAGCAATAATGATATTAATCA<br>ATGCTTATCTGATATGACTAAAATGGTACATTGTGAATATTATTTACTCGCGATCATTTATCCTCATTCTATGGTTAAATCTGATATT<br>TCAATCCTAGATAATTACCCTAAAAAATGGAGGCAATATTATGATGACGCTAATTTAATAAAATATGATCCTATAGTAGATTATTCTA<br>ACTCCAATCATTCACCAATTAATTGGAATATATTTGAAAACAATGCTGTAAATAAAAAATCTCCAAATGTAATTAAAGAAGCGAAAA<br>CATCAGGTCTTATCACTGGGTTTAGTTTCCCTATTCATACGGCTAACAATGGCTTCGGAATGCTTAGTTTTGCACATTCAGAAAAA<br>GACAACTATATAGATAGTTTATTTTTACATGCGTGTATGAACATACCATTAATTGTTCCTTCTCTAGTTGATAATTATCGAAAAATAA<br>ATATAGCAAATAATAAATCAAACAACGATTTAACCAAAAGAGAAAAAGAATGTTTAGCGTGGGCATGCGAAGGAAAAAGCTCTTG<br>GGATATTTCAAAAATATTAGGTTGCAGTGAGCGTACTGTCACTTTCCATTTAACCAATGCGCAAATGAAACTCAATACAACAAACC<br>GCTGCCAAAGTATTTCTAAAGCAATTTTAACAGGAGCAATTGATTGCCCATACTTTAAAAATTGATAAGGATCCTAATTGGTAACG<br>AATCAGACAATTGACGGCTCGAGGGAGTAGCATAGGGTTTGCAGAATCCCTGCTTCGTCCATTTGACAGGCACATTATGCATCG<br>ATGATAAGCTGTCAAACATGAGCAGATCCTCTACGCCGGACGCATCGTGGCCGGCATCACCGGCGCCACAGGTGCGGTTGCTG<br>GCGCCTATATCGCCGACATCACCGATGGGGAAGATCGGGCTCGCCACTTCGGGCTCATGAGCAAATATTTTATCTGGCTCACTC<br>AAAGGCGGTAATGAC
pAMK-857	P_{Lux}_ACE2$_{19-56}$-Npu^{C}-σ^{C} peptide sensor	AGTAAGACGGGTAAGCCTGTTGATGATACCGCTGCCTTACTGGGTGCATTAGCCAGTCTGAATGACCTGTCACGGGATAATCCG<br>AAGTGGTCAGACTGGAAAATCAGAGGGCAGGAACTGCTGAACAGCAAAAAGTCAGATAGCACCACATAGCAGACCCGCCATAAA<br>ACGCCCTGAGAAGCCCGTGACGGGCTTTTCTTGTATTATGGGTAGTTTCCTTGCATGAATCCATAAAAGGCGCCTGTAGTGCCAT<br>TTACCCCCATTCACTGCCAGAGCCGTGAGCGCAGCGAACTGAATGTCACGAAAAAGACAGCGACTCAGGTGCCTGATGGTCGG<br>AGACAAAAGGAATATTCAGCGATTTGCCCGAGCTTGCGAGGGTGCTACTTAAGCCTTTAGGGTTTTAAGGTCTGTTTTGTAGAGG<br>AGCAAACAGCGTTTGCGACATCCTTTTGTAATACTGCGGAACTGACTAAAGTAGTGAGTTATACACAGGGCTGGGATCTATTCTT<br>TTTATCTTTTTTTATTCTTTCTTTATTCTATAAATTATAACCACTTGAATATAAACAAAAAAAACACACAAAGGTCTAGCGGAATTTAC<br>AGAGGGTCTAGCAGAATTTACAAGTTTTCCAGCAAAGGTCTAGCAGAATTTACAGATACCCACAACTCAAAGGAAAAGGACTAGT<br>AATTATCATTGACTAGCCCATCTCAATTGGTATAGTGATTAAAATCACCTAGACCAATTGAGATGTATGTCTGAATTAGTTGTTTTC<br>AAAGCAAATGAACTAGCGATTAGTCGCTATGACTTAACGGAGCATGAAACCAAGCTAATTTTATGCTGTGTGGCACTACTCAACC<br>CCACGATTGAAAACCCTACAAGGAAAGAACGGACGGTATCGTTCACTTATAACCAATACGCTCAGATGATGAACATCAGTAGGGA<br>AAATGCTTATGGTGTATTAGCTAAAGCAACCAGAGAGCTGATGACGAGAACTGTGGAAATCAGGAATCCTTTGGTTAAAGGCTTT<br>TGGATTTTCCAGTGGACAAACTATGCCAAGTTCTCAAGCGAAAAATTAGAATTAGTTTTTAGTGAAGAGATATTGCCTTATCTTTTC<br>CAGTTAAAAAAATTCATAAAATATAATCTGGAACATGTTAAGTCTTTTGAAAACAAATACTCTATGAGGATTTATGAGTGGTTATTAA<br>AAGAACTAACACAAAAGAAAACTCACAAGGCAAATATAGAGATTAGCCTTGATGAATTTAAGTTCATGTTAATGCTTGAAAATAACT<br>ACCATGAGTTTAAAAGGCTTAACCAATGGGTTTTGAAACCAATAAGTAAAGATTTAAACACTTACAGCAATATGAAATTGGTGGTT<br>GATAAGCGAGGCCGCCCGACTGATACGTTGATTTTCCAAGTTGAACTAGATAGACAAATGGATCTCGTAACCGAACTTGAGAACA<br>ACCAGATAAAAATGAATGGTGACAAAATACCAACAACCATTACATCAGATTCCTACCTACATAACGGACTAAGAAAAACACTACAC<br>GATGCTTTAACTGCAAAAATTCAGCTCACCAGTTTTGAGGCAAAATTTTTGAGTGACATGCAAAGTAAGTATGATCTCAATGGTTC<br>GTTCTCATGGCTCACGCAAAAACAACGAACCACACTAGAGAACATACTGGCTAAATACGGAAGGATCTGAGGTTCTTATGGCTCT<br>TGTATCTATCAGTGAAGCATCAAGACTAACAAACAAAAGTAGAACAACTGTTCACCGTTACATATCAAAGGGAAAACTGTCCATAT<br>GCACAGATGAAAACGGTGTAAAAAAGATAGATACATCAGAGCTTTTACGAGTTTTTGGTGCATTCAAAGCTGTTCACCATGAACA<br>GATCGACAATGTAACAGATGAACAGCATGTAACACCTAATAGAACAGGTGAAACCAGTAAAACAAAGCAACTAGAACATGAAATT<br>GAACACCTGAGACAACTTGTTACAGCTCAACAGTCACACATAGACAGCCTGAAACAGGCGATGCTGCTTATCGAATCAAAGCTGC<br>CGACAACACGGGAGCCAGTGACGCCTCCCGTGGGGAAAAAATCATGGCAATTCTGGAAGAAATAGCGCTTTCAGCCGGCAAAC<br>CGGCTGAAGCCGGATCTGCGATTCTGATAACAAACTAGCAACACCAGAACAGCCCGTTTGCGGGCAGCAAAACCCGTACCGATT<br>ATCAAAAAGGATCTTCACCTAGATCCTTTTAAATTAAAAATGAAGTTTTAAATCAATCTAAAGTATATATGAGTAAACTTGGTCTGAC<br>AGTTACCAATGCTTAATCAGTGAGGCACCTATCTCAGCGATCTGTCTATTTCGTTCATCCATAGTTGCCTGACTCCCCGTCGTGTA<br>GATAACTACGATACGGGAGGGCTTACCATCTGGCCCCAGTGCTGCAATGATACCGCGAGAACCACGCTCACCGGCTCCAGATTT<br>ATCAGCAATAAACCAGCCAGCCGGAAGGGCCGAGCGCAGAAGTGGTCCTGCAACTTTATCCGCCTCCATCCAGTCTATTAATTG<br>TTGCCGGGAAGCTAGAGTAAGTAGTTCGCCAGTTAATAGTTTGCGCAACGTTGTTGCCATTGCTACAGGCATCGTGGTGTCACG<br>CTCGTCGTTTGGTATGGCTTCATTCAGCTCCGGTTCCCAACGATCAAGGCGAGTTACATGATCCCCCATGTTGTGCAAAAAAGCG<br>GTTAGCTCCTTCGGTCCTCCGATCGTTGTCAGAAGTAAGTTGGCCGCAGTGTTATCACTCATGGTTATGGCAGCACTGCATAATT<br>CTCTTACTGTCATGCCATCCGTAAGATGCTTTTCTGTGACTGGTGAGTACTCAACCAAGTCATTCTGAGAATAGTGTATGCGGCG<br>ACCGAGTTGCTCTTGCCCGGCGTCAATACGGGATAATACCGCGCCACATAGCAGAACTTTAAAAGTGCTCATCATTGGAAAACGT<br>TCTTCGGGGCGAAAACTCTCAAGGATCTTACCGCTGTTGAGATCCAGTTCGATATAACCCACTCGTGCACCCAACTGATCTTCAG<br>CATCTTTTACTTTCACCAGCGTTTCTGGGTGAGCAAAAACAGGAAGGCAAAATGCCGCAAAAAAGGGAATAAGGGCGACACGGA<br>AATGTTGAATACTCATACTCTTCCTTTTTCAATATTATTGAAGCATTTATCAGGGTTATTGTCTCATGAGCGGATACATATTTGAATG<br>TATTTAGAAAAATAAACAAATAGGGGTTCCGCGCACATTTCCCCGAAAAGTGCCACCTGACGTCTAAGAAGAATTCGCGGCCGCT<br>TCTAGAGGGAGCCAATTATTGAAGGCCTCCCTAACGGGGGGCCTTTTTTTGTTTCTGGTCTCCCGCTTAACGATCGTTGGCTGAC<br>CTGTAGGATCGTACAGGTTTACGCAAGAAAATGGTTTGTTACAGTCGAATAAAAGCTGTCACCGGATGTGCTTTCCGGTCTGATG<br>AGTCCGTGAGGACGAAACAGCCTCTACAAATAATTTTGTTTAATCCATCTCTATGGCGGATTTTATGTCATATTACCACCATCACC<br>ATCATCACATGTCAACGATCGAAGAACAGGCTAAAACGTTCCTGGATAAGTTCAATCATGAGGCGGAGGACCTGTTCTACCAAAG<br>CAGCTTGGCCTCTTGGAACTACAACACGAACATTACGGAGCGAGGAGGTAAGGGAGGACCTGGAGGTCGGGGAGGTGTTGGA<br>GGTGGTGGAGGAATTGGAGGTGGTTTTATCGCTTCCAACTGCTGGCTGGATACCCGTCCGGCACCGGATGAACAGCTGGAAGC<br>AAGCGCACAGAGCCGTCGTATGGCACAGGCACTGGATCAGCTGCCGGATCGTCAGCGTGAAGCAATTGTTCTGCAGTATTATCA<br>AGAACTGAGCAATACCGAAGCAGCAGCACTGATGCAAATTAGCGTTGAAGCCCTGGAAAGCCTGCTGAGCCGTGCACGTCGTAA<br>TCTGCGTAGCCATCTGGCCGAAGCACCGGGTGCAGATCTGAGCGGTCGTCGCAAACCGTAAAGGTGATACTTTCAGCCAAAAAA<br>CTTAAGACCGCCGGTCTTGTCCACTACCTTGCAGTAATGCGGTGGACAGGATCGGCGGTTTTCTTTTCTCTTCTCAAAGACCGTC<br>CAATGGCGGCGCGCCATCGAATGGCGCAAAACCTTTCGCGGTATGGCATGATAGCGCCCGGAAGAGAGTCAATTCAGGGTGGT<br>GAATATGAAAAACATAAATGCCGACGACACATACAGAATAATTAATAAAATTAAAGCTTGTAGAAGCAATAATGATATTAATCAATG<br>CTTATCTGATATGACTAAAATGGTACATTGTGAATATTATTTACTCGCGATCATTTATCCTCATTCTATGGTTAAATCTGATATTTCA<br>ATCCTAGATAATTACCCTAAAAAATGGAGGCAATATTATGATGACGCTAATTTAATAAAATATGATCCTATAGTAGATTATTCTAACT<br>CCAATCATTCACCAATTAATTGGAATATATTTGAAAACAATGCTGTAAATAAAAAATCTCCAAATGTAATTAAAGAAGCGAAAACAT<br>CAGGTCTTATCACTGGGTTTAGTTTCCCTATTCATACGGCTAACAATGGCTTCGGAATGCTTAGTTTTGCACATTCAGAAAAAGAC<br>AACTATATAGATAGTTTATTTTTACATGCGTGTATGAACATACCATTAATTGTTCCTTCTCTAGTTGATAATTATCGAAAAATAAATA<br>TAGCAAATAATAAATCAAACAACGATTTAACCAAAAGAGAAAAAGAATGTTTAGCGTGGGCATGCGAAGGAAAAAGCTCTTGGGA<br>TATTTCAAAAATATTAGGTTGCAGTGAGCGTACTGTCACTTTCCATTTAACCAATGCGCAAATGAAACTCAATACAACAAACCGCT<br>GCCAAAGTATTTCTAAAGCAATTTTAACAGGAGCAATTGATTGCCCATACTTTAAAAATTGATAAGGATCCTAATTGGTAACGAATC<br>AGACAATTGACGGCTCGAGGGAGTAGCATAGGGTTTGCAGAATCCCTGCTTCGTCCATTTGACAGGCACATTATGCATCGATGA<br>TAAGCTGTCAAACATGAGCAGATCCTCTACGCCGGACGCATCGTGGCCGGCATCACCGGCGCCACAGGTGCGGTTGCTGGCGC<br>CTATATCGCCGACATCACCGATGGGGAAGATCGGGCTCGCCACTTCGGGCTCATGAGCAAATATTTTATCTGGCTCACTCAAAG<br>GCGGTAATGAC
pAMK-917	P_{Lux}_Hit_sequence-Npu^{C}-σ^{C} peptide sensor	AGTAAGACGGGTAAGCCTGTTGATGATACCGCTGCCTTACTGGGTGCATTAGCCAGTCTGAATGACCTGTCACGGGATAATCCG<br>AAGTGGTCAGACTGGAAAATCAGAGGGCAGGAACTGCTGAACAGCAAAAAGTCAGATAGCACCACATAGCAGACCCGCCATAAA<br>ACGCCCTGAGAAGCCCGTGACGGGCTTTTCTTGTATTATGGGTAGTTTCCTTGCATGAATCCATAAAAGGCGCCTGTAGTGCCAT<br>TTACCCCCATTCACTGCCAGAGCCGTGAGCGCAGCGAACTGAATGTCACGAAAAAGACAGCGACTCAGGTGCCTGATGGTCGG<br>AGACAAAAGGAATATTCAGCGATTTGCCCGAGCTTGCGAGGGTGCTACTTAAGCCTTTAGGGTTTTAAGGTCTGTTTTGTAGAGG<br>AGCAAACAGCGTTTGCGACATCCTTTTGTAATACTGCGGAACTGACTAAAGTAGTGAGTTATACACAGGGCTGGGATCTATTCTT

		TTTATCTTTTTTTATTCTTTCTTTATTCTATAAATTATAACCACTTGAATATAAACAAAAAAAACACACAAAGGTCTAGCGGAATTTAC<br>AGAGGGTCTAGCAGAATTTACAAGTTTTCCAGCAAAGGTCTAGCAGAATTTACAGATACCCACAACTCAAAGGAAAAGGACTAGT<br>AATTATCATTGACTAGCCCATCTCAATTGGTATAGTGATTAAAATCACCTAGACCAATTGAGATGTATGTCTGAATTAGTTGTTTTC<br>AAAGCAAATGAACTAGCGATTAGTCGCTATGACTTAACGGAGCATGAAACCAAGCTAATTTTATGCTGTGTGGCACTACTCAACC<br>CCACGATTGAAAACCCTACAAGGAAAGAACGGACGGTATCGTTCACTTATAACCAATACGCTCAGATGATGAACATCAGTAGGGA<br>AAATGCTTATGGTGTATTAGCTAAAGCAACCAGAGAGCTGATGACGAGAACTGTGGAAATCAGGAATCCTTTGGTTAAAGGCTTT<br>TGGATTTTCCAGTGGACAAACTATGCCAAGTTCTCAAGCGAAAAATTAGAATTAGTTTTTAGTGAAGAGATATTGCCTTATCTTTTC<br>CAGTTAAAAAAATTCATAAAATATAATCTGGAACATGTTAAGTCTTTTGAAAACAAATACTCTATGAGGATTTATGAGTGGTTATTAA<br>AAGAACTAACACAAAAGAAAACTCACAAGGCAAATATAGAGATTAGCCTTGATGAATTTAAGTTCATGTTAATGCTTGAAAATAACT<br>ACCATGAGTTTAAAAGGCTTAACCAATGGGTTTTGAAACCAATAAGTAAAGATTTAAACACTTACAGCAATATGAAATTGGTGGTT<br>GATAAGCGAGGCCGCCCGACTGATACGTTGATTTTCCAAGTTGAACTAGATAGACAAATGGATCTCGTAACCGAACTTGAGAACA<br>ACCAGATAAAAATGAATGGTGACAAAATACCAACAACCATTACATCAGATTCCTACCTACATAACGGACTAAGAAAAACACTACAC<br>GATGCTTTAACTGCAAAAATTCAGCTCACCAGTTTTGAGGCAAAATTTTTGAGTGACATGCAAAGTAAGTATGATCTCAATGGTTC<br>GTTCTCATGGCTCACGCAAAAACAACGAACCACACTAGAGAACATACTGGCTAAATACGGAAGGATCTGAGGTTCTTATGGCTCT<br>TGTATCTATCAGTGAAGCATCAAGACTAACAAACAAAAGTAGAACAACTGTTCACCGTTACATATCAAAGGGAAAACTGTCCATAT<br>GCACAGATGAAAACGGTGTAAAAAAGATAGATACATCAGAGCTTTTACGAGTTTTTGGTGCATTCAAAGCTGTTCACCATGAACA<br>GATCGACAATGTAACAGATGAACAGCATGTAACACCTAATAGAACAGGTGAAACCAGTAAAACAAAGCAACTAGAACATGAAATT<br>GAACACCTGAGACAACTTGTTACAGCTCAACAGTCACACATAGACAGCCTGAAACAGGCGATGCTGCTTATCGAATCAAAGCTGC<br>CGACAACACGGGAGCCAGTGACGCCTCCCGTGGGGAAAAAATCATGGCAATTCTGGAAGAAATAGCGCTTTCAGCCGGCAAAC<br>CGGCTGAAGCCGGATCTGCGATTCTGATAACAAACTAGCAACACCAGAACAGCCCGTTTGCGGGCAGCAAAACCCGTACCGATT<br>ATCAAAAAGGATCTTCACCTAGATCCTTTTAAATTAAAAATGAAGTTTTAAATCAATCTAAAGTATATATGAGTAAACTTGGTCTGAC<br>AGTTACCAATGCTTAATCAGTGAGGCACCTATCTCAGCGATCTGTCTATTTCGTTCATCCATAGTTGCCTGACTCCCCGTCGTGTA<br>GATAACTACGATACGGGAGGGCTTACCATCTGGCCCCAGTGCTGCAATGATACCGCGAGAACCACGCTCACCGGCTCCAGATTT<br>ATCAGCAATAAACCAGCCAGCCGGAAGGGCCGAGCGCAGAAGTGGTCCTGCAACTTTATCCGCCTCCATCCAGTCTATTAATTG<br>TTGCCGGGAAGCTAGAGTAAGTAGTTCGCCAGTTAATAGTTTGCGCAACGTTGTTGCCATTGCTACAGGCATCGTGGTGTCACG<br>CTCGTCGTTTGGTATGGCTTCATTCAGCTCCGGTTCCCAACGATCAAGGCGAGTTACATGATCCCCCATGTTGTGCAAAAAAGCG<br>GTTAGCTCCTTCGGTCCTCCGATCGTTGTCAGAAGTAAGTTGGCCGCAGTGTTATCACTCATGGTTATGGCAGCACTGCATAATT<br>CTCTTACTGTCATGCCATCCGTAAGATGCTTTTCTGTGACTGGTGAGTACTCAACCAAGTCATTCTGAGAATAGTGTATGCGGCG<br>ACCGAGTTGCTCTTGCCCGGCGTCAATACGGGATAATACCGCGCCACATAGCAGAACTTTAAAAGTGCTCATCATTGGAAAACGT<br>TCTTCGGGGCGAAAACTCTCAAGGATCTTACCGCTGTTGAGATCCAGTTCGATATAACCCACTCGTGCACCCAACTGATCTTCAG<br>CATCTTTTACTTTCACCAGCGTTTCTGGGTGAGCAAAAACAGGAAGGCAAAATGCCGCAAAAAAGGGAATAAGGGCGACACGGA<br>AATGTTGAATACTCATACTCTTCCTTTTTCAATATTATTGAAGCATTTATCAGGGTTATTGTCTCATGAGCGGATACATATTTGAATG<br>TATTTAGAAAAATAAACAAATAGGGGTTCCGCGCACATTTCCCCGAAAAGTGCCACCTGACGTCTAAGAAGAATTCGCGGCCGCT<br>TCTAGAGGGAGCCAATTATTGAAGGCCTCCCTAACGGGGGGCCTTTTTTTGTTTCTGGTCTCCCGCTTAACGATCGTTGGCTGAC<br>CTGTAGGATCGTACAGGTTTACGCAAGAAAATGGTTTGTTACAGTCGAATAAAAGCTGTCACCGGATGTGCTTTCCGGTCTGATG<br>AGTCCGTGAGGACGAAACAGCCTCTACAAATAATTTTGTTTAATCCATCTCTATGGCGGATTTTATGTCATATTACCACCATCACC<br>ATCATCACATGCTGAAGCAGATTAACGTTATTGCCGGAGTTAAAGAACCCATACGCGCGTACGTGTGTAAATATGGTGAATGGTG<br>TGAGATCGTCGAAATCCGAGGAGGTAAGGGAGGACCTGGAGGTCGGGGAGGTGTTGGAGGTGGTGGAGGAATTGGAGGTGGT<br>TTTATCGCTTCCAACTGCTGGCTGGATACCCGTCCGGCACCGGATGAACAGCTGGAAGCAAGCGCACAGAGCCGTCGTATGGC<br>ACAGGCACTGGATCAGCTGCCGGATCGTCAGCGTGAAGCAATTGTTCTGCAGTATTATCAAGAACTGAGCAATACCGAAGCAGC<br>AGCACTGATGCAAATTAGCGTTGAAGCCCTGGAAAGCCTGCTGAGCCGTGCACGTCGTAATCTGCGTAGCCATCTGGCCGAAG<br>CACCGGGTGCAGATCTGAGCGGTCGTCGCAAACCGTAAAGGTGATACTTTCAGCCAAAAAACTTAAGACCGCCGGTCTTGTCCA<br>CTACCTTGCAGTAATGCGGTGGACAGGATCGGCGGTTTTCTTTTCTCTTCTCAAAGACCGTCCAATGGCGGCGCGCCATCGAAT<br>GGCGCAAAACCTTTCGCGGTATGGCATGATAGCGCCCGGAAGAGAGTCAATTCAGGGTGGTGAATATGAAAAACATAAATGCCG<br>ACGACACATACAGAATAATTAATAAAATTAAAGCTTGTAGAAGCAATAATGATATTAATCAATGCTTATCTGATATGACTAAAATGG<br>TACATTGTGAATATTATTTACTCGCGATCATTTATCCTCATTCTATGGTTAAATCTGATATTTCAATCCTAGATAATTACCCTAAAAA<br>ATGGAGGCAATATTATGATGACGCTAATTTAATAAAATATGATCCTATAGTAGATTATTCTAACTCCAATCATTCACCAATTAATTG<br>GAATATATTTGAAAACAATGCTGTAAATAAAAAATCTCCAAATGTAATTAAAGAAGCGAAAACATCAGGTCTTATCACTGGGTTTAG<br>TTTCCCTATTCATACGGCTAACAATGGCTTCGGAATGCTTAGTTTTGCACATTCAGAAAAAGACAACTATATAGATAGTTTATTTTT<br>ACATGCGTGTATGAACATACCATTAATTGTTCCTTCTCTAGTTGATAATTATCGAAAAATAAATATAGCAAATAATAAATCAAACAAC<br>GATTTAACCAAAAGAGAAAAAGAATGTTTAGCGTGGGCATGCGAAGGAAAAAGCTCTTGGGATATTTCAAAAATATTAGGTTGCA<br>GTGAGCGTACTGTCACTTTCCATTTAACCAATGCGCAAATGAAACTCAATACAACAAACCGCTGCCAAAGTATTTCTAAAGCAATT<br>TTAACAGGAGCAATTGATTGCCCATACTTTAAAAATTGATAAGGATCCTAATTGGTAACGAATCAGACAATTGACGGCTCGAGGG<br>AGTAGCATAGGGTTTGCAGAATCCCTGCTTCGTCCATTTGACAGGCACATTATGCATCGATGATAAGCTGTCAAACATGAGCAGA<br>TCCTCTACGCCGGACGCATCGTGGCCGGCATCACCGGCGCCACAGGTGCGGTTGCTGGCGCCTATATCGCCGACATCACCGAT<br>GGGGAAGATCGGGCTCGCCACTTCGGGCTCATGAGCAAATATTTTATCTGGCTCACTCAAAGGCGGTAATGAC
pAMK-924	P_{T5LacO}_His-SUMO-Hit_seq expression plasmid	AGTAAGACGGGTAAGCCTGTTGATGATACCGCTGCCTTACTGGGTGCATTAGCCAGTCTGAATGACCTGTCACGGGATAATCCG<br>AAGTGGTCAGACTGGAAAATCAGAGGGCAGGAACTGCTGAACAGCAAAAAGTCAGATAGCACCACATAGCAGACCCGCCATAAA<br>ACGCCCTGAGAAGCCCGTGACGGGCTTTTCTTGTATTATGGGTAGTTTCCTTGCATGAATCCATAAAAGGCGCCTGTAGTGCCAT<br>TTACCCCCATTCACTGCCAGAGCCGTGAGCGCAGCGAACTGAATGTCACGAAAAAGACAGCGACTCAGGTGCCTGATGGTCGG<br>AGACAAAAGGAATATTCAGCGATTTGCCCGAGCTTGCGAGGGTGCTACTTAAGCCTTTAGGGTTTTAAGGTCTGTTTTGTAGAGG<br>AGCAAACAGCGTTTGCGACATCCTTTTGTAATACTGCGGAACTGACTAAAGTAGTGAGTTATACACAGGGCTGGGATCTATTCTT<br>TTTATCTTTTTTTATTCTTTCTTTATTCTATAAATTATAACCACTTGAATATAAACAAAAAAAACACACAAAGGTCTAGCGGAATTTAC<br>AGAGGGTCTAGCAGAATTTACAAGTTTTCCAGCAAAGGTCTAGCAGAATTTACAGATACCCACAACTCAAAGGAAAAGGACTAGT<br>AATTATCATTGACTAGCCCATCTCAATTGGTATAGTGATTAAAATCACCTAGACCAATTGAGATGTATGTCTGAATTAGTTGTTTTC<br>AAAGCAAATGAACTAGCGATTAGTCGCTATGACTTAACGGAGCATGAAACCAAGCTAATTTTATGCTGTGTGGCACTACTCAACC<br>CCACGATTGAAAACCCTACAAGGAAAGAACGGACGGTATCGTTCACTTATAACCAATACGCTCAGATGATGAACATCAGTAGGGA<br>AAATGCTTATGGTGTATTAGCTAAAGCAACCAGAGAGCTGATGACGAGAACTGTGGAAATCAGGAATCCTTTGGTTAAAGGCTTT<br>TGGATTTTCCAGTGGACAAACTATGCCAAGTTCTCAAGCGAAAAATTAGAATTAGTTTTTAGTGAAGAGATATTGCCTTATCTTTTC<br>CAGTTAAAAAAATTCATAAAATATAATCTGGAACATGTTAAGTCTTTTGAAAACAAATACTCTATGAGGATTTATGAGTGGTTATTAA<br>AAGAACTAACACAAAAGAAAACTCACAAGGCAAATATAGAGATTAGCCTTGATGAATTTAAGTTCATGTTAATGCTTGAAAATAACT<br>ACCATGAGTTTAAAAGGCTTAACCAATGGGTTTTGAAACCAATAAGTAAAGATTTAAACACTTACAGCAATATGAAATTGGTGGTT<br>GATAAGCGAGGCCGCCCGACTGATACGTTGATTTTCCAAGTTGAACTAGATAGACAAATGGATCTCGTAACCGAACTTGAGAACA<br>ACCAGATAAAAATGAATGGTGACAAAATACCAACAACCATTACATCAGATTCCTACCTACATAACGGACTAAGAAAAACACTACAC<br>GATGCTTTAACTGCAAAAATTCAGCTCACCAGTTTTGAGGCAAAATTTTTGAGTGACATGCAAAGTAAGTATGATCTCAATGGTTC<br>GTTCTCATGGCTCACGCAAAAACAACGAACCACACTAGAGAACATACTGGCTAAATACGGAAGGATCTGAGGTTCTTATGGCTCT<br>TGTATCTATCAGTGAAGCATCAAGACTAACAAACAAAAGTAGAACAACTGTTCACCGTTACATATCAAAGGGAAAACTGTCCATAT<br>GCACAGATGAAAACGGTGTAAAAAAGATAGATACATCAGAGCTTTTACGAGTTTTTGGTGCATTCAAAGCTGTTCACCATGAACA<br>GATCGACAATGTAACAGATGAACAGCATGTAACACCTAATAGAACAGGTGAAACCAGTAAAACAAAGCAACTAGAACATGAAATT<br>GAACACCTGAGACAACTTGTTACAGCTCAACAGTCACACATAGACAGCCTGAAACAGGCGATGCTGCTTATCGAATCAAAGCTGC<br>CGACAACACGGGAGCCAGTGACGCCTCCCGTGGGGAAAAAATCATGGCAATTCTGGAAGAAATAGCGCTTTCAGCCGGCAAAC<br>CGGCTGAAGCCGGATCTGCGATTCTGATAACAAACTAGCAACACCAGAACAGCCCGTTTGCGGGCAGCAAAACCCGTACCGATT<br>ATCAAAAAGGATCTTCACCTAGATCCTTTTAAATTAAAAATGAAGTTTTAAATCAATCTAAAGTATATATGAGTAAACTTGGTCTGAC<br>AGTTACCAATGCTTAATCAGTGAGGCACCTATCTCAGCGATCTGTCTATTTCGTTCATCCATAGTTGCCTGACTCCCCGTCGTGTA<br>GATAACTACGATACGGGAGGGCTTACCATCTGGCCCCAGTGCTGCAATGATACCGCGAGAACCACGCTCACCGGCTCCAGATTT<br>ATCAGCAATAAACCAGCCAGCCGGAAGGGCCGAGCGCAGAAGTGGTCCTGCAACTTTATCCGCCTCCATCCAGTCTATTAATTG<br>TTGCCGGGAAGCTAGAGTAAGTAGTTCGCCAGTTAATAGTTTGCGCAACGTTGTTGCCATTGCTACAGGCATCGTGGTGTCACG<br>CTCGTCGTTTGGTATGGCTTCATTCAGCTCCGGTTCCCAACGATCAAGGCGAGTTACATGATCCCCCATGTTGTGCAAAAAAGCG<br>GTTAGCTCCTTCGGTCCTCCGATCGTTGTCAGAAGTAAGTTGGCCGCAGTGTTATCACTCATGGTTATGGCAGCACTGCATAATT<br>CTCTTACTGTCATGCCATCCGTAAGATGCTTTTCTGTGACTGGTGAGTACTCAACCAAGTCATTCTGAGAATAGTGTATGCGGCG<br>ACCGAGTTGCTCTTGCCCGGCGTCAATACGGGATAATACCGCGCCACATAGCAGAACTTTAAAAGTGCTCATCATTGGAAAACGT<br>TCTTCGGGGCGAAAACTCTCAAGGATCTTACCGCTGTTGAGATCCAGTTCGATATAACCCACTCGTGCACCCAACTGATCTTCAG

	CATCTTTTACTTTCACCAGCGTTTCTGGGTGAGCAAAAACAGGAAGGCAAAATGCCGCAAAAAAGGGAATAAGGGCGACACGGA<br>AATGTTGAATACTCATACTCTTCCTTTTTCAATATTATTGAAGCATTTATCAGGGTTATTGTCTCATGAGCGGATACATATTTGAATG<br>TATTTAGAAAAATAAACAAATAGGGGTTCCGCGCACATTTCCCCGAAAAGTGCCACCTGACGTCTAAGAAACCATTATTATCATGA<br>CATTAACCTATAAAAATAGGCGTATCACGAGGCAGAATTTCAGATAAAAAAAATCCTTAGCTTTCGCTAAGGATGATTTCTGGAAT<br>TCGCGGCCGCTTCTAGAGGGAGAACGATCGTTGGCTGAATCATAAAAAATTTATTTGCTTTGTGAGCGGATAACAATTATAATAG<br>ATTCAATTGTGAGCGGATAACAATTAGAACCCAACACCACCAGCAAGCCTAAGGAGGAGAAATATGTCATATTACCACCATCACC<br>ATCATCACGGGTCCCTGCAGGACTCAGAAGTCAATCAAGAAGCTAAGCCAGAGGTCAAGCCAGAAGTCAAGCCTGAGACTCACA<br>TCAATTTAAAGGTGTCCGATGGATCTTCAGAGATCTTCTTCAAGATCAAAAAGACCACTCCTTTAAGAAGGCTGATGGAAGCGTTC<br>GCTAAAAGACAGGGTAAGGAAATGGACTCCTTAAGATTCTTGTACGACGGTATTAGAATTCAAGCTGATCAGGCCCCTGAAGATT<br>TGGACATGGAGGATAACGATATTATTGAGGCTCACCGCGAACAGATTGGAGGTTGCATGTCATATTACGACTCCATTCCCACAAG<br>CGAGAACTTGTACTTTCAAGGGTGCATGCTGAAGCAGATTAACGTTATTGCCGGAGTTAAAGAACCCATACGCGCGTACGAAAAC<br>CTGTACTTTCAGGGTGTGTGTAAATATGGTGAATGGTGTGAGATCGTCGAAATCTAATTCAGCCAAAAAACTTAAGACCGCCGGT<br>CTTGTCCACTACCTTGCAGTAATGCGGTGGACAGGATCGGCGGTTTTCTTTTCTCTTCTCAACCAATGGCGGCGCGCCATCGAAT<br>GGCGCAAAACCTTTCGCGGTATGGCATGATAGCGCCCGGAAGAGAGTCAATTCAGGGTGGTGAATATGAAACCAGTAACGTTAT<br>ACGATGTCGCAGAGTATGCCGGTGTCTCTTATCAGACCGTTTCCCGCGTGGTGAACCAGGCCAGCCACGTTTCTGCGAAAACGC<br>GGGAAAAAGTGGAAGCGGCGATGGCGGAGCTGAATTACATTCCCAACCGCGTGGCACAACAACTGGCGGGCAAACAGTCGTTG<br>CTTATTGGCGTTGCCACCTCCAGTCTGGCCCTGCACGCGCCGTCGCAAATTGTCGCGGCGATTAAATCTCGCGCCGATCAACTG<br>GGTGCCAGCGTGGTGGTGTCGATGGTAGAACGAAGCGGCGTCGAAGCCTGTAAAGCGGCGGTGCACAATCTTCTCGCGCAAC<br>GCGTCAGTGGGCTGATCATTAACTATCCGCTGGATGACCAGGATGCCATTGCTGTGGAAGCTGCCTGCACTAATGTTCCGGCGT<br>TATTTCTTGATGTCTCTGACCAGACACCCATCAACAGTATTATTTTCTCCCATGAGGACGGTACGCGACTGGGCGTGGAGCATCT<br>GGTCGCATTGGGTCACCAGCAAATCGCGCTGTTAGCGGGCCCATTAAGTTCTGTCTCGGCGCGTCTGCGTCTGGCTGGCTGGC<br>ATAAATATCTCACTCGCAATCAAATTCAGCCGATAGCGGAACGGGAAGGCGACTGGAGTGCCATGTCCGGTTTTCAACAAACCAT<br>GCAAATGCTGAATGAGGGCATCGTTCCCACTGCGATGCTGGTTGCCAACGATCAGATGGCGCTGGGCGCAATGCGCGCCATTA<br>CCGAGTCCGGGCTGCGCGTTGGTGCGGATATCTCGGTAGTGGGATACGACGATACCGAAGATAGCTCATGTTATATCCCGCCG<br>TTAACCACCATCAAACAGGATTTTCGCCTGCTGGGGCAAACCAGCGTGGACCGCTTGCTGCAACTCTCTCAGGGCCAGGCGGT<br>GAAGGGCAATCAGCTGTTGCCAGTCTCACTGGTGAAAAGAAAAACCACCCTGGCGCCCAATACGCAAACCGCCTCTCCCCGCG<br>CGTTGGCCGATTCATTAATGCAGCTGGCACGACAGGTTTCCCGACTGGAAAGCGGGCAGTGATAATTGGTAACGAATCAGACAA<br>TTGACGGCTCGAGGGAGTAGCATAGGGTTTGCAGAATCCCTGCTTCGTCCATTTGACAGGCACATTATGCATCGATGATAAGCT<br>GTCAAACATGAGCACGCTTACTAGTAGCGGCCGCTGCAGTCCGGCAAAAAAGGGCAAGGTGTCACCACCCTGCCCTTTTTCTTT<br>AAAACCGAAAAGATTACTTCGCGTTATGCAGGCTTCCTCGCTCACTGACTCGCTGCGCTCGGTCGTTCGGCTGCGGCGAGCGGT<br>ATCAGCTCACTCAAAGGCGGTAATGAC

Supplementary Table 3: Primers used in this study

Name	Sequence	Description
osDAA154	gtgccggacgggtatccag	SAR forward primer
osDAA155	atgctgaagcagattaacgttattgcc	SAR reverse primer

Supplementary Table 4: Pap2c library analysis

Sequence	Charge	Observed m/z	Calculated m/z	# predicted macrocycles
ECMSQEACPHDDP	4	878.4136	878.4138	2
YCNNSEQCEKLDY	4	915.7013	915.6976	1
RCEMNEPCQPPDV	4	892.9417	892.9445	1
YCDTDEACTCFER	4	902.1696	902.1742	2
SCAHCENCEPHEN	4	881.1655	881.1615	2
RCKKSDNCAVTDS	4	869.9487	869.9472	2
PCLHHDRCTTSEM	4	895.4369	895.4442	2
PCTPYEMCDNFDL	4	900.4345	900.4363	1
ACPQAEQCIRFES	4	883.4451	883.4496	2
FCYERDRCLVKDT	4	924.9744	924.9773	2
PCLGLESCCILEP	4	857.9457	857.9464	1
LCNTRDPCAYYEN	4	903.6964	903.6971	2
PCSPNESCICQEL	4	868.6736	868.6793	2
TCPDWEECNTRDE	4	912.4363	912.4342	2

Supplementary References

1 Hiranuma, N. *et al.* Improved protein structure refinement guided by deep learning based accuracy estimation. *bioRxiv*, 2020.2007.2017.209643, doi:10.1101/2020.07.17.209643 (2020).

2 Wang, S., Li, W., Liu, S. & Xu, J. RaptorX-Property: a web server for protein structure property prediction. *Nucleic Acids Res* **44**, W430-435, doi:10.1093/nar/gkw306 (2016).

3 Meyer, A. J., Segall-Shapiro, T. H., Glassey, E., Zhang, J. & Voigt, C. A. Escherichia coli "Marionette" strains with 12 highly optimized small-molecule sensors. *Nature Chemical Biology* **15**, 196-204, doi:10.1038/s41589-018-0168-3 (2019).

4 Rhodius, V. A. *et al.* Design of orthogonal genetic switches based on a crosstalk map of σs, anti-σs, and promoters. *Mol. Syst. Biol.* **9**, 702, doi:10.1038/msb.2013.58 (2013).

5 Chen, Y.-J. *et al.* Characterization of 582 natural and synthetic terminators and quantification of their design constraints. *Nat. Methods* **10**, 659-664, doi:10.1038/nmeth.2515 (2013).

6 Gruber, A. R., Lorenz, R., Bernhart, S. H., Neubock, R. & Hofacker, I. L. The Vienna RNA Websuite. *Nucleic Acids Research* **36**, W70-W74, doi:10.1093/nar/gkn188 (2008).

7 Lou, C., Stanton, B., Chen, Y.-J., Munsky, B. & Voigt, C. A. Ribozyme-based insulator parts buffer synthetic circuits from genetic context. *Nature Biotechnology* **30**, 1137-1142, doi:10.1038/nbt.2401 (2012).

8 Zhang, G. *et al.* Investigation of ACE2 N-terminal fragments binding to SARS-CoV-2 Spike RBD. doi:10.1101/2020.03.19.999318 (2020).

9 Stevens, A. J. *et al.* Design of a Split Intein with Exceptional Protein Splicing Activity. *J. Am. Chem. Soc.* **138**, 2162-2165, doi:10.1021/jacs.5b13528 (2016).

10 Li, C. *et al.* Systematic mutational analysis of peptide inhibition of the p53-MDM2/MDMX interactions. *J. Mol. Biol.* **398**, 200-213, doi:10.1016/j.jmb.2010.03.005 (2010).

11 Kussie, P. H. *et al.* Structure of the MDM2 oncoprotein bound to the p53 tumor suppressor transactivation domain. *Science* **274**, 948-953, doi:10.1126/science.274.5289.948 (1996).

12 Segall-Shapiro, T. H., Sontag, E. D. & Voigt, C. A. Engineered promoters enable constant gene expression at any copy number in bacteria. *Nature Biotechnology* **36**, 352-358, doi:10.1038/nbt.4111 (2018).
