# Supplementary material for: Selection for constrained peptides that bind to a single target protein
Source: Nat Commun. 2021 Nov 3;12:6343. doi: 10.1038/s41467-021-26350-4 (PMC8566587; doi:10.1038/s41467-021-26350-4)
Supplement: Supplementary file 2 — Reporting Summary [file 41467_2021_26350_MOESM2_ESM.pdf]

nature portfolio

Corresponding author(s): Christopher A. Voigt

Last updated by author(s): Sep 20, 2021

Reporting Summary

Nature Portfolio wishes to improve the reproducibility of the work that we publish. This form provides structure for consistency and transparency in reporting. For further information on Nature Portfolio policies, see our Editorial Policies and the Editorial Policy Checklist.

Statistics

For all statistical analyses, confirm that the following items are present in the figure legend, table legend, main text, or Methods section.

n/a	Confirmed	
☐	☒	The exact sample size (*n*) for each experimental group/condition, given as a discrete number and unit of measurement
☐	☒	A statement on whether measurements were taken from distinct samples or whether the same sample was measured repeatedly
☒	☐	The statistical test(s) used AND whether they are one- or two-sided<br>*Only common tests should be described solely by name; describe more complex techniques in the Methods section.*
☒	☐	A description of all covariates tested
☒	☐	A description of any assumptions or corrections, such as tests of normality and adjustment for multiple comparisons
☐	☒	A full description of the statistical parameters including central tendency (e.g. means) or other basic estimates (e.g. regression coefficient) AND variation (e.g. standard deviation) or associated estimates of uncertainty (e.g. confidence intervals)
☒	☐	For null hypothesis testing, the test statistic (e.g. *F*, *t*, *r*) with confidence intervals, effect sizes, degrees of freedom and *P* value noted<br>*Give P values as exact values whenever suitable.*
☒	☐	For Bayesian analysis, information on the choice of priors and Markov chain Monte Carlo settings
☒	☐	For hierarchical and complex designs, identification of the appropriate level for tests and full reporting of outcomes
☒	☐	Estimates of effect sizes (e.g. Cohen's *d*, Pearson's *r*), indicating how they were calculated

Our web collection on statistics for biologists contains articles on many of the points above.

Software and code

Policy information about availability of computer code

Data collection	LC-MS data acquisition: Masshunter version 10.0.<br>Flow cytometry data: BD FACSDiva version 8.0.3<br>NGS data acquisition: Illumina HCS version 2.2.68<br>BLI data acquisition: Octet Data Acquisition version 9.0
Data analysis	BLI data analysis: ForteBio Data Analysis version 9.0<br>LC-MS data analysis: Masshunter version 10.0<br>Cytometry data anlysis: Cytoflow version 1.0<br>Python version 3.9

For manuscripts utilizing custom algorithms or software that are central to the research but not yet described in published literature, software must be made available to editors and reviewers. We strongly encourage code deposition in a community repository (e.g. GitHub). See the Nature Portfolio guidelines for submitting code & software for further information.

Data

Policy information about availability of data

All manuscripts must include a data availability statement. This statement should provide the following information, where applicable:

- Accession codes, unique identifiers, or web links for publicly available datasets
- A description of any restrictions on data availability
- For clinical datasets or third party data, please ensure that the statement adheres to our policy

Source data are provided with this paper for the following figures: Fig1b, Fig1c, Fig1d, Fig2f, Fig3a, Fig3b, Fig3c, Fig3d, Fig4d, Fig4f, Fig4i, Fig4j, Fig4k, SI Fig 1. Genetic part sequences are available in Supplementary Information. Key plasmids and strains are available from Addgene.

Field-specific reporting

Please select the one below that is the best fit for your research. If you are not sure, read the appropriate sections before making your selection.

☒ Life sciences ☐ Behavioural & social sciences ☐ Ecological, evolutionary & environmental sciences

For a reference copy of the document with all sections, see nature.com/documents/nr-reporting-summary-flat.pdf

Life sciences study design

All studies must disclose on these points even when the disclosure is negative.

Sample size	Biological experiments were performed in triplicate on separate days. Statistical methods were not used to predetermine sample size. We found 3 replicates gave highly reproducible results for cytometry and BLI.
Data exclusions	For cytometry measurements, gating was completed by fitting a 2D Gaussian function to the FSC and SSC distributions and excluding all events greater than three standard deviations from the mean.
Replication	All experimental claims have been tested three times and on different days for biological replicates. All attempts at replication were successful.
Randomization	Grouping was not relevant to this study as all strains were analyzed individually and compared with others.
Blinding	Blinding was not relevant to this study as all strains were analyzed individually and compared with others.

Reporting for specific materials, systems and methods

We require information from authors about some types of materials, experimental systems and methods used in many studies. Here, indicate whether each material, system or method listed is relevant to your study. If you are not sure if a list item applies to your research, read the appropriate section before selecting a response.

Materials & experimental systems

n/a	Involved in the study	
☒	☐	Antibodies
☒	☐	Eukaryotic cell lines
☒	☐	Palaeontology and archaeology
☒	☐	Animals and other organisms
☒	☐	Human research participants
☒	☐	Clinical data
☒	☐	Dual use research of concern

Methods

n/a	Involved in the study	
☒	☐	ChIP-seq
☐	☒	Flow cytometry
☒	☐	MRI-based neuroimaging

Flow Cytometry

Plots

Confirm that:

☒ The axis labels state the marker and fluorochrome used (e.g. CD4-FITC).

☒ The axis scales are clearly visible. Include numbers along axes only for bottom left plot of group (a 'group' is an analysis of identical markers).

☒ All plots are contour plots with outliers or pseudocolor plots.

☒ A numerical value for number of cells or percentage (with statistics) is provided.

Methodology

Sample preparation	Strains were grown in 1 ml of LB+ antibiotics for 20 hr in a deep well 96-well plate (1896-2000, USA Scientific, FL, USA) at 30 "C, 900 rpm in an lnfors HT Multitron Pro (lnfors USA, MD, USA). Cultures were then diluted 1:100 into fresh 1 ml of LB+ antibiotics and serial 1:10 dilutions of inducers (IPTG, 10-3-103 µM; 3O6-AHL, 10-3-103 nM) for 20 hr in a deep well 96-well plate at 30 "C, 900 rpm in the Multitron Pro. 0.5 µl of saturated cell culture were then diluted into 200 µl of PBS containing 1 mg/ml kan for cytometry analysis.
Instrument	BD LSR Fortessa flow cytometer with the HTS attachment (BD, Franklin Lakes, NJ, USA).
Software	BD FACSDiva version 8.0.3.<br>Cytoflow version 1.0
Cell population abundance	At least 30,000 events were collected for each sample .
Gating strategy	Gating was not performed on cells. Representative forward and side scatter plot is available in the SI.

☒ Tick this box to confirm that a figure exemplifying the gating strategy is provided in the Supplementary Information.
